# IPET and FETR: Experimental Approach for Studying Molecular Structure Dynamics by Cryo-Electron Tomography of a Single-Molecule Structure

**DOI:** 10.1371/journal.pone.0030249

**Published:** 2012-01-24

**Authors:** Lei Zhang, Gang Ren

**Affiliations:** Molecular Foundry, Lawrence Berkeley National Laboratory, Berkeley, California, United States of America; University of Washington, United States of America

## Abstract

The dynamic personalities and structural heterogeneity of proteins are essential for proper functioning. Structural determination of dynamic/heterogeneous proteins is limited by conventional approaches of X-ray and electron microscopy (EM) of single-particle reconstruction that require an average from thousands to millions different molecules. Cryo-electron tomography (cryoET) is an approach to determine three-dimensional (3D) reconstruction of a single and unique biological object such as bacteria and cells, by imaging the object from a series of tilting angles. However, cconventional reconstruction methods use large-size whole-micrographs that are limited by reconstruction resolution (lower than 20 Å), especially for small and low-symmetric molecule (<400 kDa). In this study, we demonstrated the adverse effects from image distortion and the measuring tilt-errors (including tilt-axis and tilt-angle errors) both play a major role in limiting the reconstruction resolution. Therefore, we developed a “focused electron tomography reconstruction” (FETR) algorithm to improve the resolution by decreasing the reconstructing image size so that it contains only a single-instance protein. FETR can tolerate certain levels of image-distortion and measuring tilt-errors, and can also precisely determine the translational parameters via an iterative refinement process that contains a series of automatically generated dynamic filters and masks. To describe this method, a set of simulated cryoET images was employed; to validate this approach, the real experimental images from negative-staining and cryoET were used. Since this approach can obtain the structure of a single-instance molecule/particle, we named it individual-particle electron tomography (IPET) as a new robust strategy/approach that does not require a pre-given initial model, class averaging of multiple molecules or an extended ordered lattice, but can tolerate small tilt-errors for high-resolution single “snapshot” molecule structure determination. Thus, FETR/IPET provides a completely new opportunity for a single-molecule structure determination, and could be used to study the dynamic character and equilibrium fluctuation of macromolecules.

## Introduction

The dynamic character of proteins dictates their functions and ultimately represents an accurate portrayal of their many “personalities” [1], [2]. A snapshot of proteins frozen in crystals reveals a single, unique structure that is often used as a blueprint for studies in structure–function relationships. However, these structures fail to encompass the dynamic nature of proteins in solution. Protein dynamics involves both equilibrium fluctuations that regulate biological function and other non-equilibrium effects of biological motors, which convert chemical energy to mechanical energy.

Although the experimental approach to determine the dynamic structure at atomic-resolution level is not available, molecular dynamics (MD) simulations have been widely used to link structure and dynamics by enabling the exploration of the conformational energy landscape accessible to protein molecules [1], [2]. MD simulations could provide detailed dynamics and function in structure, but one of the major obstacles of MD is a potential energy barrier to determine global protein conformations and equilibrium fluctuations.

Cryo-electron tomography (cryoET) is an experimental approach to provide a structural snapshot of a single-instance biological object from a series of tilted viewing angles [3], [4]. This method has been rapidly adopted and applied to reveal the three-dimensional (3D) structure of cells, bacteria, and even proteins. Unfortunately, the resolution of 3D density maps rarely goes beyond 30 Å using conventional ET reconstruction methods [5], and is generally insufficient to determine domain information of single-instance protein. An alternate cryoET approach to improve the resolution of protein structure is a 3D classification and averaging method in which hundreds to thousands of 3D subvolumes are selected from a large-volume, low-resolution 3D reconstruction [6]. This highly used method can reduce noise and improve the 3D subvolume reconstruction resolution up to 20 Å when the protein has a high symmetry, such as GroEL and nuclear pores [7]. However, when the protein has no symmetry, but with multiple-conformational structures, such as a human IgG antibody and the high-density lipoprotein (HDL), the classification and orientation determination of subvolumes are challenging. Moreover, the average of hundreds of different conformational structures could be detrimental to elucidate the structural equilibrium fluctuations of a protein.

We believe, there are adverse effects from image distortion and measuring tilt-errors in conventional tomography reconstruction methods, which play a major role in limiting the resolution of the large-size whole micrograph reconstruction. The image distortions (introduced by lens astigmatism [8], energy filter [9], [10], radiation-induced deformations [11] and defocus-related distortion) can generate the displacement (translational errors) and measuring tilt-errors (including tilt-axis and tilt-angle errors). Since measuring tilt-angle is usually performed by tracking the movements of gold fiducial markers between the tilt micrographs, the inconsistency of displacement introduced by image distortion results in the inconsistency of movement of markers during tilting, therefore resulting in an inconsistency of tilt-angles. The final determined tilt-angle is actually an average of tilt-angles suggested from different areas of the micrograph. Greater distortion results in greater displacement, thus producing a larger tilt-angle error. Image distortion is generally a large-scale deformation/displacement, in which different areas within a micrograph present a different amount of displacement. Mathematically, large-scale deformation/distortion can be represented by a combination of local displacement (translational error), rotation (tilt-axis error) and tilting (tilt-angle error). For example, in two-dimensional (2D) cryo-crystallography, the uneven supporting substrate can frequently introduce a large-scale distortion of 2D crystal. The distortion introduces a displacement and rotation against space. The distortion can be determined and corrected by a so-called “unbending” image-processing method [12], [13]. In brief, the whole micrograph image is broken into a series of small-size images to calculate the translation/displacement and rotation of each small image via a comparison of each small image to the average image/reference. Shifting and rotating back each small image according to its determined displacement and rotational parameters can correct the distortion of large-size crystal/micrograph by merging these shifted/rotated small-images together. However, in cryoET reconstruction, either the lattice or the averaged reference is not available to correct the distortion of the large-size micrograph. The measuring tilt-errors (including the displacement-introduced tilt-error and the distortion-introduced small rotation and tilting) can limit the resolution of the large-size whole micrograph reconstruction. A simple model to estimate the tilt-errors is to treat them as a random vibration within a small range, such as ±0.5°. This requires a new strategy that can tolerate these tilt-errors and can precisely determine/correct the translational errors/displacement for high-resolution cryoET reconstruction.

Here, we report a new strategy and robust algorithm, focused ET reconstruction (FETR) that can tolerate small tilt-errors and accurately determine two translational parameters of each image. FETR is an iterative refinement procedure that includes a series of automatically generated dynamic filters and masks for enhancing the convergence of the reconstruction. To limit the adverse effects from the tilt-errors, we reduced the size of reconstruction ET images by containing only a single-instance of a protein particle, rather than large whole-micrograph images that contain dozens of proteins from conventional methods. Hence, we named this approach individual-particle electron tomography (IPET) [14]. We believe that, with the same uncorrectable tilt-errors, the maximum translational error (displacement) within a small image is much smaller than within a large whole-micrograph. The small image-size can limit the translational error from measuring tilt-errors, which can further limit the adverse effects from tilt-errors in the 3D reconstruction.

To describe the IPET method and FETR algorithm, a set of simulated cryoET images were generated and used. To validate this reconstruction method, four sets of real experimental ET data consisting of two sets of an antibody (molecular weight: ∼150 kDa) imaged by negative-staining ET, and two sets of nascent HDL (molecular weight: 140–240 kDa, protein portion: 56–84 kDa) imaged by cryoET [15] were used for 3D reconstruction of each targeted single-instance protein. All four 3D reconstructions displayed abundant structural details, such as the domains. The most interesting feature of this method is that the 3D reconstruction of each individually targeted molecule instance is free of conformational dynamics and heterogeneity, proving that the 3D structure can be treated as a snapshot of the dynamic structure of the macromolecule. By comparing these “snapshot” structures, this method could allow the study of macromolecular structural dynamics [16], [17], [18]. The global protein conformations and equilibrium fluctuations can be used as a constraint for MD simulation to reveal the structural detail in protein function and mechanism.

## Methods

### 1 Generating simulated cryoET data

Build-up of a targeted object: To introduce the IPET method and FETR algorithm, a set of simulated cryoET data was generated from a known-answer object. The targeted object is a single instance of protein, a fragment (A–D chains, molecular weight: ∼108 kDa) of molybdate transporter (ModB_2_C_2_) from *Archaeoglobus fulgidus* (PDB entry 2ONK [19]). The 3D density map of the object was generated at a resolution of 2 Å within a box of 160×160×160 voxels by the “*pdb2mrc*” command (EMAN software package) [20], in which each pixel corresponds to 1 Å in the specimen (a magnification of ∼100 kX when using a 4 k×4 k CCD camera). The feature of using this targeted object is that it contains various structural features that can be used to evaluate the reconstruction at various resolutions, such as a donut-like overall shape (∼100 Å in longest diameter), central hole (a diameter of ∼30 Å), 12 transmembrane α-helix domains (∼10–20 Å), and short α-helices and β-strands (∼8 Å and beyond).

**Figure 1:**
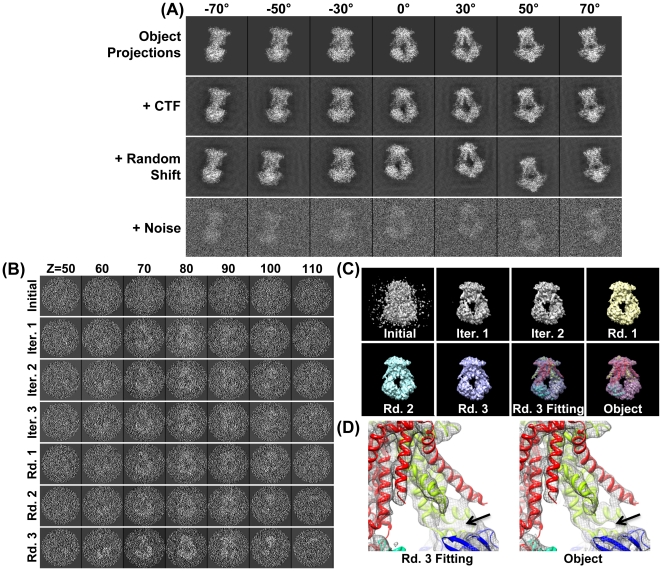
Simulating cryoET images and 3D reconstruction by IPET/FETR. (A) An object, a 3D density map of a single-instance protein (molybdate, portion of PDB entry 2ONK) was projected into a total of 141 tilt series of 2D images by following the simulated cryoET tilt-angles (containing both tilt-axis and tilt-angle errors in a range of ±0.5°), in which seven projections were presented (first row); to simulate contrast transfer function (CTF), CTF at a defocus of 1.5 µm was applied to each image and then deconvoluted (second row); to simulate the translation errors, each image was randomly shifted with a maximal radius range of 30 pixels (third row); to simulate the noise, Gaussian noise (SNR = 0.2) was added to each image (last row). These images were used as the cryoET “raw” images for 3D reconstruction. (B) By FETR, the reference-free initial model was generated by directly back-projecting the “raw” images according to the measuring tilt-angles (containing immeasurable tilt-errors). The convergence of 3D reconstruction was approached by iterations through three major rounds. Selected ET slices of the 3D reconstruction of initial model, iteration one to three, round one to three, were displayed. (C) The isosurfaces of the corresponding 3D reconstructions were also displayed after they were low-pass filtered to 8 Å. The initial model displayed as a globular noisy blob, while the quality of reconstructions had been greatly improved after the 1–2 iterations. The reconstructions of each round contained many structural details, such as the α-helices. Docking the crystal structure into the isosurface from the final reconstruction displayed a near perfect match and no distinguishing differences from the object. (D) Zoom-in views of docked final reconstruction and object displayed slight difference as indicated by the arrows.

Simulating cryoET images: CryoET images are the snapshots of the structure of a single-instance biological specimen viewed from a series of tilt-angles. To simulate the cryoET angles (including tilt-axis angles and tilt-angles), the following simple model is used. We assumed that the “measured/reconstruction” tilt-axis angle is 0° since the tilt-axis is pre-aligned and parallel to the Y-axis of CCD, and assumed the “measured/reconstruction” tilt-angle used for 3D reconstruction is a set of integral numbers from −70° to +70° in step of 1° (**Figure S1**). The “real/experimental” tilt-axis and tilt-angle are unknown, but must contain errors that cannot be further determined or corrected by current experimental techniques. A simple model to simulate the tilt-errors (including tilt-axis errors and tilt-angle errors) is treating them as a vibration and random error within a range of ±0.5°, thus, the “real/experimental” tilt-axis angle is a random number within a range of ±0.5°, while the “real/experimental” tilt-angle is the “measured” tilt-angle (integral numbers from −70 to +70) plus a random number within a range of ±0.5° as the tilt-angle (**Figure S1**). These “real/experimental” tilt-axis and tilt-angles are only used to tilt the object for a set of 141 projections (noise-free simulated ET images) by the “*PJ 3Q*” command (SPIDER software package) [21]. Notably, each projection contains an indefinable/uncorrectable random tilt-error (**Figure 1A**). The tilt-axis error may also contain a systematic tilt-axis error (1°–5°) because not all tilt-axis can be pre-aligned to Y-axis of CCD, and the effect of this systematic tilt-axis error is discussed in subsection 1 of Discussion Section.

EM images always contain the effects of defocus-related contrast transfer function (CTF). To simulate CTF effects on the cryoET images, a common CTF curve is convoluted to each projection as a simple approach to simulate the CTF effect to demonstrate the cryoEM reconstruction methodologies [20], [22]. The CTF curve parameters were chosen based on the parameters of our real cryoET experimental images of nascent HDL [15]. Nascent HDL is a ∼140–240 kDa small particle embedded in a physiological buffer and imaged under the defocus of ∼1.5 µm and total dose of ∼140 e^−^/Å^2^ by a high-tension of 120 kV (2.2 mm spherical aberration) FEI T12 cryoEM [15]. Notably, using a low defocus value (<1.5 µm) instead of a commonly used high defocus value (∼4 µm) is a part of our optimizing strategy, because we believe, at a lower defocus, CTF reversed the phases less frequently in reciprocal space than at a high defocus, and can provide more complete structural information for 3D reconstruction. For CTF correction, the CTF curve is deconvoluted from each simulated cryoET image by the “*TF CTS*” command in SPIDER software (**Figure 1A**) [21]. Although, the real cryoET experimental images at tilt angles contain a focus gradient across the image perpendicular to the tilt-axis, considering the image size we used is relatively small (160 Å in size) and the maximum defocus difference across the image is no more than ∼0.015 µm under a tilt angle of 70° (the maximum defocus variation is less than ∼1%) it is reasonable to ignore the defocus gradient across the image by using a common CTF curve in simulating cryoET images.

The particle centers in cryoET images normally do not overlap with the image centers. The difference between the particle center and image center is the translational error. To simulate translational errors, we randomly shifted particle centers within a radius of 30 pixels (**Figure 1A**). Considering that the image size was only 160 pixels, translational errors of up to 30 pixels are rather significant in the simulated cryoET images.

To simulate the noise in the cryoET images, Gaussian type noise was used, despite the limitations representing the noise as real cryoEM images. For instance, the variation in tilting angles produce different ice thicknesses that can result in a variation of signal-to-noise ratio (SNR) among the tilting images, while the different parts of the particle along the projection direction include different amounts of water molecules, resulting in an uneven distribution of SNR within an image. Although the variation of SNR among the tilting images can be minimized by an “exponential” exposure time scheme on a constant ice thickness area [23], the constant thickness of ice cannot be obtained in the real cryoET experiment. The uneven distribution of SNR within the image cannot be simulated by addition of Gaussian noise. Considering the sole purpose of using the simulation cryoET data is only for reporting a new reconstruction method, the Gaussian noise, a simple model, is chosen to roughly represent the noise in the cryoET images as that conventionally used in reporting the single-particle cryoEM reconstruction methods [20], [22].

A Gaussian noise SNR of ∼0.2 is chosen and applied to all of the above simulated images, and since SNR = 0.2 it is within the range of our real cryoET experimental image measurement of 0.1–0.2 (the standard deviation (SD) of density measured by the SPIDER “*fs*” command, is ∼58.3–65.1 in the HDL particle area and is ∼53.7 in the background area) (**Figure S2**). To simulate this SNR = 0.2 noise, a Gaussian noise with SD 5 times higher than the particle was added to each image (**Figure 1A**) using the “*MO*” and “*AD*” commands in the SPIDER software package [21], [24].

The final simulated cryoET images (**Figure 1A**) contain geometric tilt-errors (including tilt-axis and tilt-angle errors) within ±0.5°, translational errors within ±30 pixels, high-level noise (SNR = 0.2), a missing wedge (tilt up to ±70°, the details of the missing-wedge effect to 3D reconstruction is discussed in **Information S1**) and CTF effects (defocus 1.5 µm). Although the simulation has limitations in simulating real cryoET images, such as the defocus gradient, variation of SNR, the envelope function related to the particular EM instrument, the error in determination the CTF, and the real noise, the simulation data still has its value for verifying the program and evaluating the variation between the reconstruction and the known object considering that the simulation data will be used to demonstrate the reconstruction methodology only.

### 2. Basic tools for image processing basic tools

**Figure 2:**
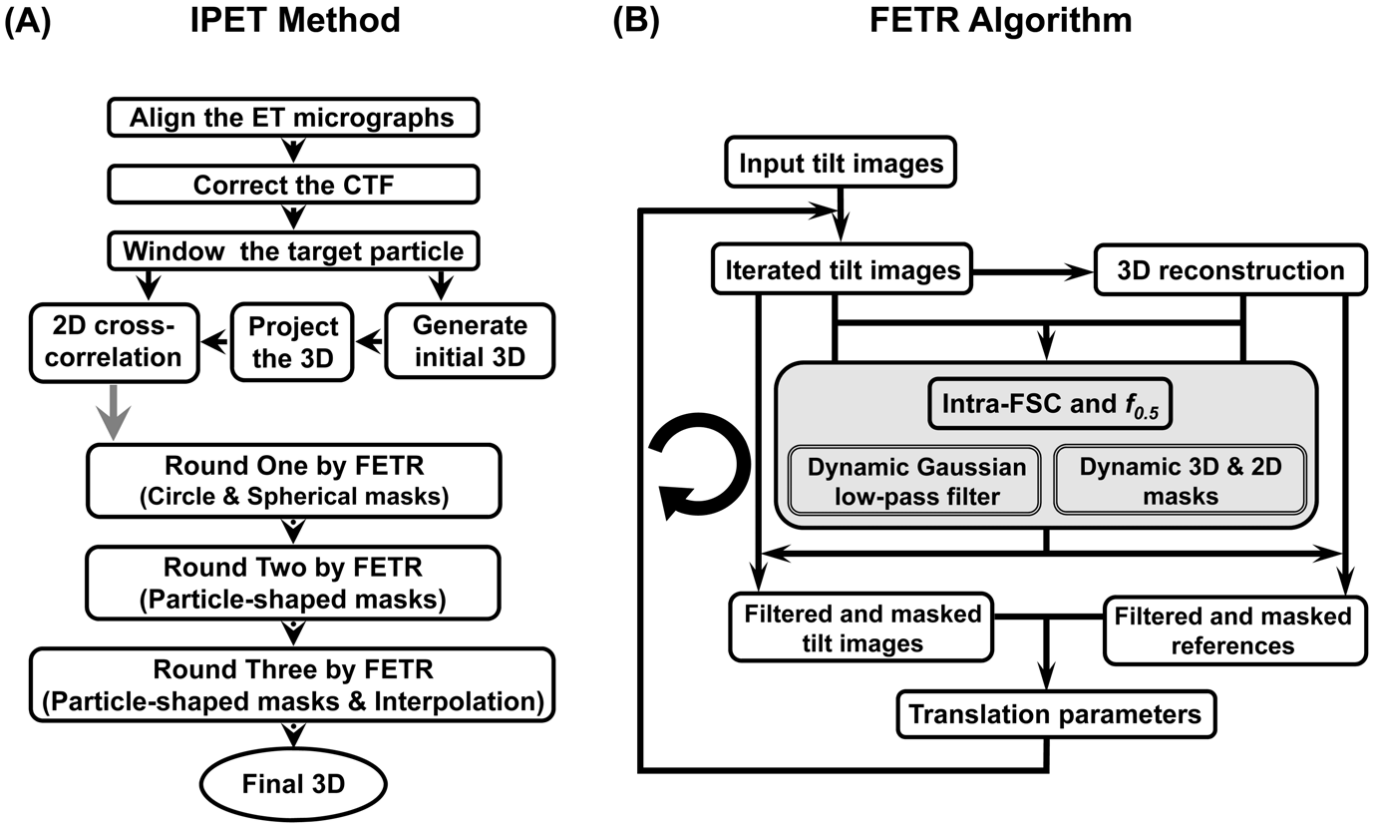
Flow diagram of individual-particle electron tomography (IPET) method and focused electron tomography reconstruction (FETR) algorithm. (A) The IPET method contains two phases: the electron tomography (ET) data collection with image preprocessing, and a focused electron tomography reconstruction (FETR) algorithm. In the first phase, the single-instance of particle was imaged by ET. The contrast transfer function (CTF) of the whole-micrograph-size tilt images was determined, and then corrected after tilt images were aligned. The small images containing only a targeted particle were selected and windowed from each of tilt whole-micrograph, and then directly back-projected into 3D as the initial/starting model for refinement. The 3D reconstruction refinement procedure contains three rounds of refinement loops in the FETR algorithm. Each round was essentially the same, except different masks were applied. In the first round, a series of automatically generated circular Gaussian-edge masks was used; in the second round, the automatically generated particle-shaped masks were used and while, in the third round, the last mask in second round was used with association of an additional interpolation method during determining the translation parameters. (B) Each round of refinement loops contains the same iteration algorithm, FETR. 3D reconstruction from the previous iteration (or initial model for the first iteration) was projected, and the projections were used as references for the next iteration. Before translational parameter searching, a dynamic Gaussian low-pass filter and automatically generated mask were applied to both the references and tilt images.

The IPET method/FETR algorithm includes an iterative refinement computational process. Each iteration contains several image-processing steps (**Figure 2**), such as generating the filter and mask to reduce noise, computing the new parameters for next iteration, and analyzing the 3D reconstruction variation/convergence. Some of the most important tools used are described below.

Fourier shell correlation (FSC): To quantitatively analyze the variation and convergence of 3D reconstruction generated in each iteration, FSC analyses were used [25], [26], [27] in conventional single-particle reconstruction [20], [21], [28]. Instead of splitting the class averages into two groups in single-particle reconstruction, the raw ET images are split into two groups based on having an odd- or even-numbered index in the order of tilt angle. Each group is used to generate a 3D reconstruction and then the two 3D reconstructions are used to compute the FSC curve over the corresponding spatial frequency shells in Fourier space (“*RF 3*” command in SPIDER). Since this FSC computation uses a single set of ET images, we call it intra-FSC to distinguish it from the regular FSC computed between the 3D reconstruction and object. The frequency at which the intra-FSC curve falls to a value of 0.5 (called intra-*f_0.5_*) was used to represent the resolution of an iterated 3D reconstruction [20], [21], [29]. The intra-*f_0.5_* value will be used to generate related parameters in the next iteration (details described below).

Dynamic Gaussian low-pass filter: Our strategy to determine the translational errors/parameters is to use low-resolution information for initial searching, and then gradually use increasingly higher resolution information for further searching. To control the resolution information contributed by each iteration, a set of automatically generated Gaussian low-pass filters were used to reduce unnecessary structural details (**Figure 2B**). A series of 21 filters was used sequentially. A pair of boundary frequencies, the low-pass frequency (*f_low_*) and cut-off frequency (*f_cut_*), are defined as below, as
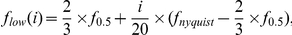


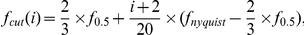

*where i = 0, 1, …, 20. f_0.5_* is the intra-*f_0.5_* defined in the previous iteration. Filters with increasingly higher pairs of boundary frequencies (*i = 0,1,…,20*) were used in subsequent iterations for progressively increasing high-frequency information and accuracy to determine the translational parameters. Notably, the filter is dynamic, as the filter boundary frequencies are a function of the intra-*f_0.5_* value that is a reflection of the quality of 3D reconstruction in the previous iteration. If the reconstruction of the previous iteration converged well (i.e., intra-*f_0.5_* moved to a higher frequency), the filter automatically moves to including higher-frequency image information during translational searching. In contrast, if the reconstruction converged poorly (i.e., intra-*f_0.5_* moved to lower frequency), the filter automatically moves to include lower frequencies to increase the weight of low-resolution information during translational searching. As a result, the filter automatically corrects for poorly defined translation parameters in the previous iteration.

Circular mask: The masking technique has been widely used in X-ray crystallography [30] and single-particle reconstruction (such as the “*amask*” options in the “*refine*” command and the “*automask*” options in the “*proc3d*” command of the EMAN software package) [20], [31], [32]. In the first round of our reconstruction iterations, a circular mask with a Gaussian edge was applied to each tilt image to reduce the effects of noise and remove excess background areas (**Figure 2**). The mask is generated based on the rough size and shape of the object. The mask should keep the intensity/density of raw image within object area, but reduce (not cut off) the density outside the object (mostly background noise). For example, a protein molecule with a size of roughly 100 pixels can generate a circular mask with a density value of 1.0 within a 120-pixel-diameter circle and density of 0.5 outside a 160-pixel-diameter circle (the largest circle that can fit in the image). The density value between these two circles is defined by the gradient of a Gaussian function.

Dynamic particle-shaped mask: A particle-shaped 3D mask and its projections (2D masks) were used to further reduce the noise and unnecessary background in iterations (**Figure 2**). In contrast to circular masks, the particle-shaped masks are generated based on the low-resolution structure of 3D reconstruction. The 3D reconstruction is filtered by a Gaussian low-pass filter based on the intra-*f_0.5_* value of the previous iteration. The density is modified as follows: a) the densities outside an isosurface (described below) are reset to 0.0, while densities inside the isosurface are reset to 1.0; b) the modified density map is low-pass filtered to very low resolution (such as ∼60–80 Å) to generate the particle-shaped mask (thus, the densities near the mask boundary are modified as a gradient boundary by this low-pass filter). A total of six isosurfaces is used to generate the 3D masks that are sequentially used in the following iterations. The major difference among the masks is their isosurface containing space volume. The largest volume is defined as half the volume of the largest sphere that can fit within a box of 160×160×160 voxels. The smallest volume is defined as three times the mass volume of the targeted protein molecule (the volume calculation is based on an estimated weight-to-volume ratio of 1.35 g/ml, i.e. 0.81 Da/Å^3^ [20]). Our experience was that a tight/small mask can reduce the noise efficiently, but an overly tight mask may result in truncation of the edges of targeted object. Thus, the smallest mask we used should be safe enough to avoid this truncation. The remaining four isosurface volumes are interpolated between those of the largest and smallest masks by the following rule: The inverse values of these four volumes are evenly distributed between the inverse values of the maximum and minimum mask volumes. The 2D mask that will be applied to a tilt image is generated by projecting the 3D mask according to a corresponding tilt angle of the tilt image, with one 2D mask applied to each tilt image. Since the 3D masks are generated by a Gaussian low-pass filter and the filter parameters are dependent on the intra-*f_0.5_* value of the previous iteration and the 2D and 3D masks depend on the convergence of the previous iteration (evaluated by the intra-*f_0.5_* value), we call these dynamic masks.

### 3. Focused electron tomography reconstruction algorithm

#### 3.1 Image distortion and measurement tilt-errors are major obstacles in achieving high resolution in conventional electron tomographic reconstruction methods

Image distortion limits cryoET reconstruction resolution [33]. Image distortion has numerous causes, such as astigmatism [8], projector lens [34], pincushion and spiral [35], [36], energy filter [9], [10], and even radiation-induced deformations [11] along with defocus of non-parallel beam conditions. The distortion can result in measurement errors of tilt-axis and a tilt-angle that can never be eliminated.

Taking the example of defocus-introduced distortion, images under different defocus have slightly different magnification under non-parallel-beam EM operation conditions. To quantitatively demonstrate the change in magnification resulting from change in defocus, we imaged nanogold particles with a Gatan UltraScan 4 k×4 k CCD camera equipped on a Tecnai 20 transmission electron microscope (Philips Electron Optics/FEI) operating at 200 kV. The 5 nm gold particles were deposited on the carbon-film coating on an EM grid. Tracking the coordinate changes of ∼70 nanogold particles under defocus changes from 0.0 µm to 10 µm in steps of 0.5 µm we were able to determine the changing ratio. In brief details, the micrographs under different defocus were aligned together based on their cross-correlation calculation (**Video S1**), and then the coordinates of ∼70 nanogold particles in each aligned micrograph were fitted with a second-degree polynomial function by a least squares fitting method using MATLAB. The fitted parameters shown in **Table S1** suggest that, other than the constant numbers, *a_0_* and *b_0_*, only two linear parameters, *a_1_* and *b_2_* have substantial change against defocus. By plotting *a_1_* and *b_2_* against defocus, the distributions of *a_1_* and *b_2_* were similar to each other and flowing in a line, suggesting the particle coordinate change is nearly a linear relationship to focus change. Thus, a linear equation was used to fit those *a_1_* and *b_2_* data and shown in **Figure S3.** The analysis showed that magnification changed ∼8% as the defocus changed by 10 µm (**Figure S3**). Within a tilt image, the defocus is different only along the direction perpendicular to the tilt axis. The defocus difference can be as large as ∼1.1 µm from image edge to image center (4096 pix×5.6 Å/pix×tan(45°)/2) for a 4 k CCD image at a tilt-angle of 45° (imaged under a magnification of 20 kX with a pixel ratio of 5.6 Å/pix). The ∼1.1 µm defocus could result in a maximum of ∼0.85% distortion that corresponds to ∼0.5° measuring tilt-angle error. The calculation is based on the equation: Δ*θ* = cos^−1^[(1–0.85%)×cos(45°)]−45°≈0.5° (details discussed in **Figure S4**). Similarly, other distortions can also introduce the tilt angle error in measuring tilt angles, and these tilt-errors can limit the reconstruction resolution when using whole micrographs.

The magnification changed by ∼8% when the defocus changed ∼10 µm as measured by our Tecnai T20 microscope under a specific illuminating α-angle condition (spot size = 7, illumination area is just covering the CCD size for a desired brightness and coherence beam). Note that the magnification change can be different depending on the microscope and illumination condition (including non-parallel beam, and even misalignment). For instance, using the T20 microscope under the parallel beam condition, the magnification change can be less than 1%. However, the parallel beam condition does not fit to our desired condition in cryoET image acquisition because the beam is either too large in size, too weak in brightness or too poor in coherence (too large spot-size for a desired brightness). If the parallel beam is a necessary condition for cryoET imaging or the 8% defocus-related magnification change is related to misalignment of microscope, the experimental observation (**Video S1, Figure S3** and **Table S1**) suggests the defocus-related magnification changes needs to be addressed during reconstruction, especially for high-resolution cryoET reconstruction. Our purpose in discussion of the defocus-introduced image distortion is to provide an example for many other image distortions (such as astigmatism, projector lens, pincushion and spiral, energy filter and radiation-induced deformations) to demonstrate how the image distortion can lead to a tilt-angle measurement error.

Conventional cryoET reconstruction methods call for using the whole micrograph for reconstruction [37], [38], [39], [40], [41], [42] because it is believed that more signal in whole micrographs can provide more information for more accurate determination of the tilt-angle of each micrograph. We believe that image distortion limits the accuracy in determining the tilt-angles. The tilt-errors can directly affect the accuracy of the particle centers in whole micrograph reconstruction. Given the same tilt-errors, much less displacements occur near the reconstructing center than near the corner, and the reduction of displacement within the reconstructing subvolume, which yields a better quality 3D reconstruction. For example, a 0.5° error in tilt-axis will result in ∼25-pixel (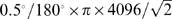) displacement error in the particle center at the corner of a 4 k×4 k image, which will definitely induce errors in the reconstruction. In contrast, the 0.5° error in tilt-axis will result in less than 1.6-pixel (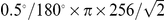) displacement error in the particle center at the corner of a 256×256 pixel small-image, which will induce much less error in the 3D reconstruction. A similar experience in the cryoEM single-particle reconstruction is the reconstructed subvolume near the central area has generally better quality than far from the center (i.e. corner and edge areas). More detailed discussion about how tilt-error affects the cryoEM 3D reconstruction is given in subsection 1 of Discussion Section. According to the above statements, we developed this IPET/FETR to limit the effects from tilt-error by reducing reconstruction image-size to a small size that includes only a single-instance protein particle for 3D reconstruction.

#### 3.2 Overall view of focused electron tomography reconstruction algorithm

The principle behind our focused refinement reconstruction algorithm is essentially similar to the single-particle reconstruction method. In single-particle reconstruction, five parameters of each image need to be determined: three Euler angles (i.e., φ, ψ and θ) and two translation parameters (Δx and Δy) [20], [21], [28], [43], [44]. In our reconstruction method, the first Euler angle φ (particle angle), which represents the in-plane rotation angle of object to the tilt-axis, is equal to 0° because all tilt images share one targeted object. The second Euler angle ψ (tilt-axis angle), which corresponds to the angle between the tilt-axis and micrograph Y-axis in SPIDER definition [21], is approximately 0° (**Figure S1**) because the CCD is pre-aligned to be parallel to the tilt-axis in our microscope (in the case of the CCD being closely pre-aligned to tilt-axis, but with a small systematic error will be discussed in subsection 1 of Discussion Section). Although tilt-axis is aligned to 0°, the mechanical vibration during tilting can result in a small random error in tilt-axis. A conservative estimate of the tilt-axis error is within ±0.5°. The third Euler angle θ (tilt-angle), which corresponds to the tilting angle, is the only angle that varies majorly among tilt images. Although the tilt-angle θ can be read from the goniometer of the microscope or measured by tracking the movement of fiducial markers, neither of the methods can provide an accurate value for θ, as θ must contain an error resulting from the image distortions described above. A conservative estimate of the tilt-angle error is similar to tilt-axis error, i.e. within a range of ±0.5°, so we used these ranges to generate simulated cryoET images. Our tomography reconstruction strategy in reducing the adverse effects from the tilt-errors (tilt-axis and tilt-angle errors) is to reduce the reconstruction image size by including only the targeted particle, hence the name: focused ET reconstruction (FETR) process, where only two translation parameters (Δx and Δy) need to be defined.

The key procedure in FETR is to accurately determine the translational parameters (Δx and Δy) of each particle in each image that is precisely aligned to a global center (**Figure 2**). The 3D reconstruction obtained from the previous iteration (or initial model) is tilted and projected by the measured tilt-angles (that contain immeasurable errors). The generated projections are used as the references to search for the translation parameters (Δx and Δy) by a reciprocal space cross-correlation calculation (“*CC N*” command in SPIDER). Notably, each projection is used only once as a reference for the tilt image that shares the same tilt-angle. Prior to searching, a dynamic filter and a mask were applied to both the references and the tilt images.

Three rounds of refinement (the coarse refinement round, the fine refinement round, and the oversampling refinement round) were used to determine two translation parameters (Δx and Δy) (**Figure 2**). Each round contains multiple iterative refinement procedures as described below.

#### 3.3 Focused electron tomography reconstruction

**Figure 3:**
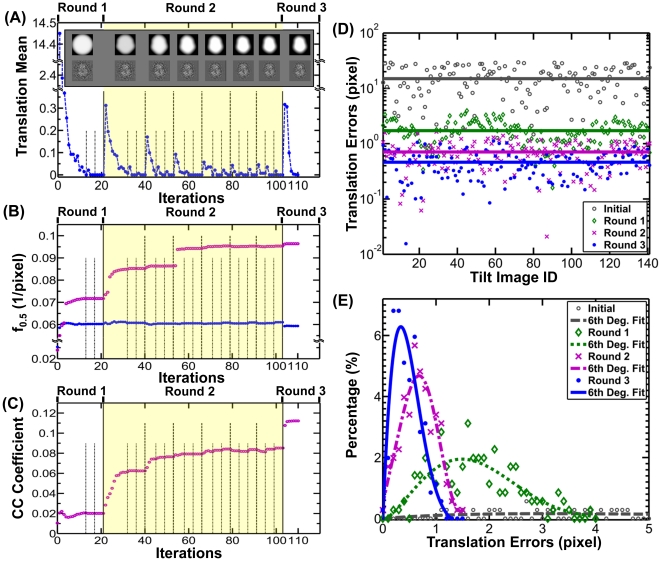
Monitoring the convergence of the iterations. Four parameters were used to monitor the progress of the iterations, translation mean, *f_0.5_* values, cross-correlation coefficients (CC C), and translation errors. (A) The translation mean was the sum of the translation distances of all the images divided by the total number of images. The result showed that the translation means approached zero gradually. During the iteration, various sizes and shapes of masks were used and displayed. (B) Two types of the *f_0.5_* values were used to monitor the progress. One type (blue points), called intra-*f_0.5_*, was calculated based on the iterated tilt images during the iteration and used to monitor the progress of the iterations. The result showed the intra-*f_0.5_* values quickly improved in the first several iterations, but had no significant improvement afterward. Another type (purple circles) was a conventional *f_0.5_* value that was calculated based on the iterated 3D reconstruction and the object. The result showed that the *f_0.5_* values gradually improved through the iterations. (C) The CC C between each iteration 3D reconstruction and the object was computed, which also showed that the quality of reconstructions was gradually improved. (D) The distribution of the translation errors and (E) the histogram of the translation errors were used to analyze the translation parameters determined in each round. In the simulated raw images, the translation errors were evenly distributed with a mean of 14.96 pixels (gray line). After the first round, the translation errors were quickly reduced to a mean of 1.72 pixels with a peak population at 1.48 pixels (blue line). After the second round, the errors continued to be reduced to a mean of 0.71 pixels with a peak at 0.68 pixels (purple line). The errors were further minimized to a mean of 0.46 pixels with a peak at 0.34 pixels after the third round (blue line).

Generating a reference-free initial model is necessary to start the iterative refinement. A reference-free initial model was generated from the raw (original) ET images themselves by a reciprocal space back-projection algorithm (“*BP 3F*” command in SPIDER). The reference-free initial model in ET slices displayed a noisy reconstruction with no obvious structural details, except a slightly higher density region near the center (**Figure 1B**). Its isosurface showed a central globular blob with a noisy surface and suggested no useful structural information other than the rough size of the object (**Figure 1C**). The intra-FSC analysis of the initial model showed that the value of intra-*f_0.5_* was ∼1/40 Å^−1^ (**Figures 3B** **and S5A**).

The first round is a coarse refinement with circular masks and dynamic filters. In this round, a total of 21 dynamic Gaussian low-pass filters and a set of circular Gaussian-edge masks were automatically generated and applied to both the reference and tilted image.

In the first iteration, we generated the references by projection from the initial model, and then we applied the first Gaussian low-pass filter (*i = 0*) and the circular mask to both the references and raw images to compute their cross-correlation peaks to determine the translation parameters of each raw image (**Figure 3A**, top left corner). The determined translation parameters (after rounding to integers in order to avoid the error from interpolation) were applied to the corresponding raw images to generate the first-iteration 3D reconstruction (**Figure 1B and 1C**). The translation-shifted raw images were split into two groups and used to generate two independent 3D reconstructions to compute the variation and determine the first-iteration intra-*f_0.5_* value (**Figure 3B**, blue points). The intra-*f_0.5_* value was used for the related parameters to generate the second-iteration filter (*i = 0*). A further analysis of the convergence was to calculate the mean of the translation parameters (before rounding) in the current iteration. The translation mean, 14.45 pixels/image, suggested that the first iteration had dramatically recovered error in the particle centers (**Figure 3A**). Thus, it was not surprising that the first-iteration 3D reconstruction revealed the overall shape of the object (**Figure 1B and 1C**).

The second iteration was essentially the same as the first iteration, except for i) using the first-iteration 3D reconstruction rather than reference-free initial model to generate the references; and ii) using the first-iteration intra-*f_0.5_* to automatically generate the second-iteration filter parameters by the equations shown in the subsection 2 of Methods Section. Although the second-iteration filter (*i = 0*) equation was identical to that of the first iteration, the second-filter parameter intra-*f_0.5_* was modified by the first-iteration intra-*f_0.5_* instead of the initial intra-*f_0.5_*, which normally allows for better resolution. The second-iteration filter would actually include more high-resolution information during the second iteration. The determined translation parameters (after rounding to integers) were used to generate the second-iteration images and 3D reconstruction (**Figure 1B and 1C**). The second-iteration 3D reconstruction had no obvious visual difference from the first-iteration 3D reconstruction after both 3D reconstructions were filtered to 8 Å. However, analysis of the intra-*f_0.5_* value suggested the second-iteration reconstruction had better resolution and significantly less variation than the first-iteration reconstruction (**Figure 3B**, blue points).

We repeated the above iteration procedure a minimum of four times until the change of translation mean was below 0.01 pixels per image (termination criteria). After the 13^th^ iteration, the change had decreased dramatically from 14.45 pixels to below 0.01 pixels for the first time (**Figure 3A**). The iteration process was restarted with more high-resolution information by automatically replacing the first filter (*i = 0*) with the second filter (*i = 1*) and using a finer Gaussian low-pass filter. The iterations hit the termination criteria again after 17^th^ iteration, during which the change dropped to below 0.01 pixels (**Figure 3A**).

Iteration continued through low-pass filters, including higher and higher resolution structural information. However, past a certain point, higher-resolution information does not necessarily contribute to greater accuracy in determining the translational parameters. The same termination criteria (<0.01 pixel per image) were used after the first three filters were used. In this round, a total of 21 iterations were conducted before hitting the termination criteria (**Figure 3A**).

The ET slices of the 21^st^-iteration 3D reconstruction displayed a clear density in the center slices (**Figure 1B**). The isosurface of the 3D reconstruction (after filtered to 8 Å), at the contour level that contains a volume equal to the initial model volume (70% space volume of the protein molecule, at which level the secondary structure could clearly be seen), showed significant structural detail, such as a hole and some α-helices (**Figure 1C**). Intra-FSC analysis showed that intra-*f_0.5_* improved significantly (from 1/40 Å^−1^ to 1/16.6 Å^−1^) (**Figure 3B**, blue points). This analysis suggests that the 3D reconstruction gradually converged and the translation parameters were determined precisely.

The second round is a fine refinement with particle-shaped masks and dynamic filters. The second round was essentially the same as the first round except for the use of a particle-shaped 2D mask instead of a circular mask (**Figures 3A** top panel and **S6**). Unlike the circular mask, the particle-shaped 2D masks applied to each tilt image were different from one another. Each mask was only applied to the one reference and one tilt image that shared the same reconstruction angle prior to calculating the cross-correlation peak to determine the translational parameters.

In this round, a total of six particle-shaped 3D masks were generated and applied sequentially from largest volume to smallest volume (**Figures 3A** top panel and **S6**). Other than the difference in shape among the 3D masks, the major difference among the masks was their space volumes. Their mask volumes corresponded to protein molecular weights of 800 kDa, 600 kDa, 480 kDa, 400 kDa, 340 kDa, and 300 kDa (**Figure S6**). These mask volumes were chosen such that the inverse numbers of these volumes were evenly distributed.

Using each particle-shaped 3D mask, we repeated the same procedure as described in the first round. A total of 82 iterations were conducted (**Figure 3A, 3B, 3C**). The analysis of the changes of translation mean showed the maximum change was 0.32 pixels/image while using the largest mask and then dropping to 0.05 pixels/image and using the smallest mask (**Figure 3A**). Although the intra-FSC analysis showed no obvious improvement in the intra-*f_0.5_* value (**Figure 3B**, blue points), the center slices of the reconstruction showed an obvious improvement in contrast (**Figure 1B**). The 3D reconstruction showed more structural detail than the reconstruction from the first round (**Figure 1C**).

The translation mean changes demonstrated that these masks were very effective for translation parameter determination. However, the masks should be monitored and confirmed that no portion of the particle was truncated (**Figure S6**). Although the last 3D mask contained the smallest volume (3 times the object volume) (**Figures 3A** **and S6**), considering the iteration nearly converged (the average change was significantly below 1.0 pixel/image), this mask is still safe to be used.

The third round is an oversampling refinement. In the above two rounds, the translation parameters were converted to integers before being applied to the images. This was important in order to avoid the error introduced from the multiple interpolations. However, the accuracy of determining these translation parameters was also limited by the use of integer values. In this round, we released the integer restriction by using an oversampling technique to further improve the reconstruction (**Figure 2**). Expansion of the image size by 10 times in each dimension was performed using the triangular interpolation technique (“*IP T*” command in SPIDER), and then continued through the iterations following the last iteration of the second round (**Figure 3A**, top right corner). In this process, the corresponding 2D masks were also interpolated by 10 times before being applied to the expanded references and the images prior to determine the translational parameters. To avoid computer memory overflow, the images and masks were shrunk by a factor of 5 in each dimension before back-projection. We repeated this iteration seven times until the change of translation mean between two successive iterations was below 0.001 pixels/image.

The center ET slices of the final 3D reconstruction showed significantly better contrast than the last reconstruction from the second round (**Figure 1B**), despite intra-FSC analysis showing no obvious improvement in the intra-*f_0.5_* value (**Figure 3B**). The isosurface of the last 3D reconstruction (after filtered to 8 Å) displayed many more important structural details (**Figure 1C and 1D**) than that of the last reconstruction of the second round, suggesting that the reconstruction had achieved further improvement with the oversampling technique.

## Results

### 1. Variation analysis of the FETR reconstruction of simulated cryoET data

Variation analysis was conducted from two aspects: translational parameters and 3D reconstruction. To analyze the deviation of translation parameters from their “ideal” centers, we calculated the difference between the ideal center (**Figure 1A**) and the center defined from IPET/FETR. A variation analysis of these “absolute translation errors” was performed by displaying each absolute translation error of each image against its image index. The distribution showed the absolute translation error in the initial raw images was distributed evenly in the range of 30 pixels with a mean of 14.96 pixels/image (**Figure 3D**, gray line). However, after the first round, the absolute translation error was significantly reduced to a mean of 1.72 pixels/image (**Figure 3D**, green line). After the second round, the absolute translation error continued to be reduced to a mean of 0.71 pixels (**Figure 3D**, purple line), and was reduced further to a mean of 0.46 pixels after the third round (**Figure 3D**, blue line). This analysis suggests that the absolute translation error was gradually minimized.

A further variation analysis of absolute translation error was performed by computing the histogram. The histogram showed that ∼95% of the images had a translation error below ∼3.5 pixels/image after the first round, reduced to ∼1.4 pixels/image, and further decreased to below ∼1.0 pixels/image after the second and third round, respectively. In the process, the peak population is ∼4.87% with an error of ∼1.48 pixels/image after the first round (**Figure 3E**, green dotted line), which increased to ∼11.70% with a smaller error of ∼0.68 pixels/image (**Figure 3E**, purple dash-dot line), and further rose to ∼15.72% with an even smaller error, ∼0.34 pixels/image (**Figure 3E**, blue solid line) after the second and third round. Both the above analyses suggested that the variation of the translation parameters was relatively low after the three rounds of iterations.

Variation analysis of 3D reconstruction was also conducted by methods: Fourier space FSC analysis and real-space cross-correlation analysis. In Fourier space analysis, the FSC curve between each 3D reconstruction (**Figure 1C**) and the ideal object was computed, and was further used to determine *f_0.5_* value. The *f_0.5_* distribution showed that: i) in the first iteration, even though the *f_0.5_* value was ∼1/40 Å^−1^ (consistent with the intra-*f_0.5_* value), the initial model had little similarity with the object (**Figure S5B**, gray circle-dash line) except for roughly correct dimensions (**Figure 1C**); ii) in the last iteration of the first round, the *f_0.5_* value had quickly improved to beyond ∼1/14 Å^−1^ (**Figure 3B**, purple circles) and the 3D reconstruction showed significantly more similarity to the object (**Figure S5B**, green diamond-dash line) and contained many structural details, such as the central hole and some α-helices (**Figure 1C**); iii) in the second and third rounds, the *f_0.5_* value further improved to beyond ∼1/11 Å^−1^. 3D reconstructions of the object also showed further improvement (**Figure S5B**, purple cross-dash line and blue point-dash line). The 3D reconstruction contained more structural details than that of the first round, and remarkably showed almost all α-helices that had emerged, suggesting that the 3D reconstruction had converged to the object (**Figures 1C and 1D**). The intra-*f_0.5_* value showed no significant improvement after the first round (**Figures 3B** blue points and **S5A**), but the *f_0.5_* value calculated between the 3D reconstruction and the ideal object showed significant improvement after the first round. This suggests that the intra-*f_0.5_* value was not sensitive enough to be used for defining the 3D resolution or used as an iteration termination criterion. For this reason, we used the change in translation mean error as the termination criterion.

To minimize the mask effect on FSC calculation in the variation analysis, we used the circular-shaped mask with density-gradient boundary instead of the particle-shaped mask. The outer diameter of the circular mask equaled the size of image. The special particle-shaped mask was only used for searching for the translational parameters. Despite the mask enhancing the intra-FSC correlation, the resolution calculated from the circular-mask-applied images was significantly lower than the resolution calculated from the FSC between the reconstructed 3D map and object. Namely, even the circular mask generated an overly optimistic correlation; the resolution is still much poorer than the real resolution of the 3D map should be.

As a further variation analysis of the 3D reconstruction, the real-space cross-correlation coefficient (CC C) between each iterated 3D reconstruction and the object was calculated (“*CC C*” command in SPIDER). The CC C distribution showed that, although the initial model had little similarity to the object (CC C value is 0.01) (**Figure 3C**), the similarity had improved to 0.02 after the first round, and further improved to 0.08 and 0.11 after the second and third rounds, respectively (**Figure 3C**). Unfortunately, the CC C value was generally lower than we expected, perhaps due to the influence of a high-noise background. By applying a circular mask (radius of 70 pixels) to the reconstruction before calculating CC C, these CC C values were increased significantly (**Figure 3C**). In summary, both variation analyses showed that the iteration robustly corrected the translational errors and improved the 3D quality during iterations and refinements even when the immeasurable tilt-errors exist.

### 2. Validation of the focused ET reconstruction method by real experimental data

#### 2.1 3D reconstruction of an antibody by negative-staining ET

To validate IPET method and FETR algorithm, we applied this method to a set of real experimental data, with antibody negative-staining (NS) images. The antibody is naturally dynamic, fluctuates frequently and is structurally heterogeneous. The structural heterogeneity makes the study of the structure and function difficult using current technologies such as x-ray crystallography, nuclear magnetic resonance (NMR), and even EM single-particle reconstruction because all of these techniques need the average signal from thousands to millions of individual particles. One of the features of our IPET method is that the 3D reconstruction is from an individually targeted, single-instance of molecule/protein that is free of conformational dynamics and heterogeneity. The 3D structure can be treated as a “snapshot” of the dynamic structure.

**Figure 4:**
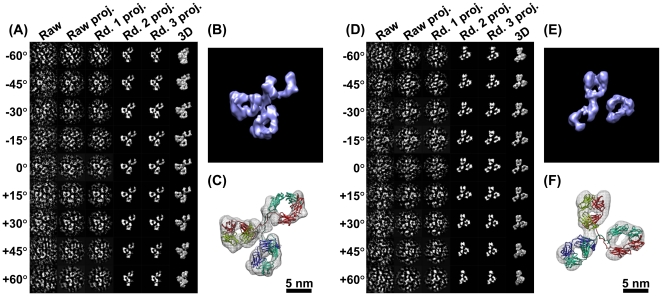
3D reconstructions with negative staining of an IgG antibody by IPET. (A) A single-instance of an IgG antibody was imaged by negative staining (NS) ET. Nine tilted views of the same single IgG particle were selected from 81 tilt micrographs that were CTF corrected by TOMOCTF, and then displayed in leftmost column. By FETR algorithm, the 3D reconstruction was iterated and converged. The corresponding tilting projections of the 3D reconstruction from major iterations were displayed beside the raw images in six columns. (B) The final 3D reconstruction of a targeted individual antibody particle was displayed in a contour level that contained a volume corresponding to 160 kDa. The reconstruction displayed three ring-shaped domains that corresponding to three domain of IgG antibody. (C) Docking the crystal structure (PDB entry 1IGT) of each domain of the IgG antibody into each ring-shaped density of IgG showed a good fit. The docking was performed by a rigid-body docking option in Chimera program [79]. The loops between the domains were simply connected by Chimera. (D) Another example of the IPET method in a 3D reconstruction of another single-instance of a targeted individual IgG antibody particle. The NS-ET images of the raw images and projections of the related reconstruction were also displayed. (E) The 3D reconstruction of this antibody particle displayed a similar structural feature to the first one, such as the three domains. (F) Docking the IgG antibody crystal structure into the density map showed a good fit as well.

The human IgG antibody (molecular weight: ∼150 kDa) sample was prepared with an optimized NS protocol [45], [46], and imaged using the ET technique on an FEI T20 microscope under 80 kX magnification and defocus of less than 2 µm [18]. By tracking and windowing a targeted single-instance of an antibody particle from each tilt micrograph after CTF correction by TOMOCTF [47], we reconstructed its 3D density map (**Figures 4** and **S7, S8, S9, S10**) with our IPET method/FETR algorithm. To further validate the method, we windowed the images of another targeted single-instance of antibody particle and reconstructed it into 3D (**Figures 4** and **S7, S8, S9, S10**). The 3D density maps contained rich structural details (**Figures 4B and 4E**), including the shape of each domain, and even the holes inside each domain. Intra-FSC analysis showed that the resolution was ∼15 Å (**Figure S9**). Note, the actual resolutions were generally much better than that defined from the intra-FSC described in subsection 1 of Results Section. Although the resolution from intra-FSC is not as high as what we achieved in the simulated cryoET image, the resolution is, as far as we know, the highest resolution map ever obtained by tomography reconstruction.

Since a NS image involves a coating stain and the shape of the coating stain represents the surface structure of protein, we asked if the resolution measurement represented information on the protein rather than just good resolution of the stain structure. The NS protocol used in preparation of the antibody sample is from our recently published optimized protocol [45], [46], and we demonstrated that with our protocol, we could generate lipoprotein particles that have similar size and shape to that of cryoEM [45], [46]. All three domains of antibody show the same sort of hole in the middle—two holes are similar in size, but one hole is obviously bigger than other holes. The holes have been revealed by the crystal structure of the IgG antibody. Displaying the crystal structure of the IgG antibody (PDB entry 1IGT) by two methods—the ribbon and Van der Waals surface—both views show holes in the F_ab_ domain (**Figure S11**, the top two domains) and the F_c_ domain (**Figure S11**, the bottom domain). Remarkably, the F_c_ domain hole is much bigger than the holes in F_ab_ domain, consistent with our 3D reconstruction.

To determine further if the holes were artifacts of defocus-related CTF, we imaged the antibody sample under Scherzer focus condition. The survey NS image (after filtered) and selected particles show the holes in all three domains can obviously be visualized directly (**Figures S12A** and **S12B**), and the corresponding holes can also be visualized from the crystal structure under similar orientation (**Figure S12C**). Thus, the holes were less likely caused by CTF artifacts. The NS image suggested our stain of uranyl formate (UF) can penetrate the protein surface and display the internal structure. This result was consistent with our observation in lipoproteins [45], [46], but challenged the conventional wisdom and concept that negative staining can only show the surface structure information of coated proteins.

Despite the detailed structure of the holes, the spatial and orientation relationships among the domains are reliable. The 3D reconstruction from single-instance antibody that is free of conformational dynamics and heterogeneity can be treated as a “snapshot” of the dynamic structure of antibody. By comparing these “snapshot” IgG antibody structures, this method could allow us to study antibody dynamics. For example, by aligning two docked PDB files by aligning their F_c_ domain, the F_ab_ domains are different from each other in location and orientation, suggesting the equilibrium fluctuation and structural dynamic character of IgG antibody (**Figures 4C and 4F**, **Video S2**).

#### 2.2 3D reconstruction of high-density lipoprotein by cryoET

To further validate our method on high-noise real cryoET experimental data, we applied this method to the structure determination of nascent high-density lipoprotein (HDL) [16], [17]. A nascent HDL sample was prepared as described [15], [45], [48], [49]. HDL particles *in vivo* vary in size, shape, components, and biological functions [15], [45], [48], [49], [50]. A particular component of these, called nascent discoidal HDL (molecular weight: 140–240 kDa) is the disk-shaped precursor of mature spherical HDL and contains phospholipids, and 2 to 3 apolipoprotein A-I (apoA-I, molecular weight: 28 kDa) molecules per particle. This kind of discoidal HDL is a critical intermediate between lipid-poor apoA-I and mature spherical HDL during HDL assembly. However, HDL structure determination is complicated by the dynamic nature and heterogeneity of HDL [15], [45].

**Figure 5:**
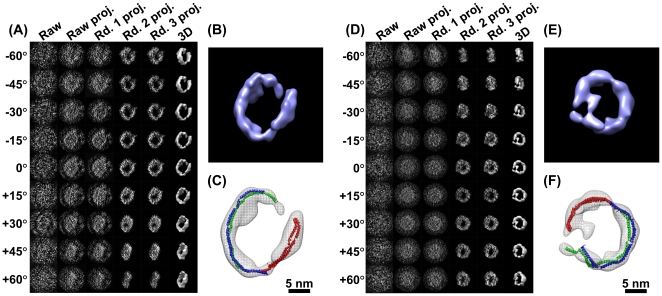
3D reconstructions of a cryoET 17 nm nascent HDL particle by IPET. (A) An individual targeted nascent HDL particle embedded in vitreous ice was imaged by the cryoET technique. The tilt images of a targeted particle were windowed from the tilt series of cryoET micrographs after the CTF was corrected by TOMOCTF. Nine represented tilted views of a targeted 17 nm HDL particle were shown in leftmost column. These selected tilt images were from 81 tilt micrographs that were taken at the specified tilt angles. The other six columns in each grid showed the results of progressive refinement from certain iterations. (B) Reconstructed to 3D density map. The high-density portion corresponding to proteins, apolipoprotein A-I (apoA-I), was formed in a ring shape. (C) By docking the double-helices into the ring-shaped density, the size suggests three apoA-I molecules exist in a 17 nm HDL particle. (D) Another targeted 17 nm HDL particle was imaged and reconstructed for 3D density map. (E–F) The 3D reconstruction showed the similar structural feature to the first HDL particle, suggesting our cryoET reconstructions are consistent as reported by cryoEM observation [51].

By IPET method, we used the cryoET technique to image the nascent HDL particles that were embedded in vitreous physiological buffer (**Figure S2**) under defocus of ∼1.5 µm as described [15]. This sample contained 17 nm nascent HDL particles. To demonstrate the effectiveness of the FETR algorithm, we targeted two 17 nm nascent HDL particles for 3D reconstruction (**Figures 5** and **S13, S14, S15, S16**). We focused on the 17 nm HDL particle because the 17 nm HDL is the only HDL fraction that has been investigated by cryoEM [51]. Dr. van Antwerpen's group examined the 17 nm HDL particles embedded in vitreous ice from orthogonal tilt views and found that the 17 nm HDL particle has a discoidal shape (**Figure S17**) [51].

The cryoET images of the targeted 17 nm rHDL particle were manually windowed from CTF-corrected (by TOMOCTF) cryoET micrographs [47]. The particle images were noisy, but the particles could be visualized (**Figure 5A and 5D**). Using FETR, the particle centers were precisely aligned to their “global center” by associating them with a series of automatically generated masks. To ensure the particle-shaped masks in the second round of FETR did not cut off any portion of the particle, the raw particle images were monitored after masks were applied (**Figure S16**). The slices of the 3D reconstruction were also monitored before and after we applied low-pass filters and/or masks (**Figures S13A, S13B, S13C, S13D**, **S14A, S14B, S15,S14D**). Although the slice views are very noisy, such as with the central slice (Z = 50 shown in **Figure S13A**, and Z = 100 shown in **Figure S14A**), by simply applying a low-pass filter (50–60 Å), the particle immediately stood out from the background while the noise was immediately reduced (shown in Z = 50 of **Figure S13B**, and Z = 100 of **Figure S14B**). This was due to the noise presented in the slice is high-frequency noise. This low-pass filtered 3D was then used to generate the particle-shaped mask with a volume of ∼3–8 times greater than the HDL molecular volume by following same procedure as described in subsection 2 of Methods Section. By applying this mask on the 3D reconstruction, the projections of the 3D reconstruction had significantly higher SNR (the 4^th^ panel shown in **Figure 5A and 5D**). The improvement of SNR in projections was due to the cut-off of these noises that were originally overlapping with the particle along the projection direction, but not overlapping in 3D space. To ensure the final mask did not cut off the particle, the final 3D reconstructions before and after applying the final mask were displayed (**Figures S13E** and **S13F**, and **S14E** and **S14F**). Only a few small isolated densities were excluded by the final mask (shown in gray, **Figures S13E** and **S14E**).

By FETR, the noise in the final 3D projections was rapidly eliminated and the particle signal was quickly enhanced (**Figure 5A and 5D**). Both final 3D reconstructions (**Figure 5B and 5E**) displayed many common features, such as a discoidal shape and a high-density portion in the form of a ring. The high-density portion in the density map corresponds to the high-density component of HDL, i.e., apolipoprotein A-Is. It is commonly believed that apoA-I forms a double-helical bundle that wraps around the hydrophobic portion of the lipid bilayer in HDL [15], [52]. Two helices in the ring-shaped density cannot be distinguished from the reconstructions because their center distance is only ∼10 Å.

Although the intra-*f_0.5_* defined resolution is usually lower than the actual resolution (discussed in the subsection 1 of Results Section), the intra-*f_0.5_* defined ∼36–42 Å resolutions of nascent HDL maps (**Figure S15**) are not different from the resolution obtained by conventional cryoET methods. However, considering that nascent HDL particles have molecular mass ∼200 kDa and ∼200 kDa protein particles are rarely successfully reconstructed by even the single-particle reconstruction method, the successful reconstruction of a 3D density map of a single-instance ∼200 kDa HDL particle is remarkable. The anti-parallel α-helices formed into a ring-shape from cryoET is amazingly consistent with the structures i) shown in the raw cryoET images of nascent HDL particle (**Figure S2**) [15], ii) shown in conventional cryoEM images that acquired from two orthogonal tilted angles reported by van Antwerpen (**Figure S17**) [51], and iii) the structural model derived from the energy minimization by molecular dynamic simulations [15], [52], suggesting that our IPET method/FETR algorithm is a powerful tool for cryoET reconstruction. We believe that, by further optimizing the cryoET imaging conditions to more closely match the simulated cryoET conditions, a higher resolution structure of a single-instance nascent HDL can be expected.

Despite the resolution, the 3D reconstruction from a single-instance HDL particle that is free of conformational dynamics and heterogeneity can be treated as a “snapshot” of the dynamic structure of protein. By comparing these “snapshot” HDL structures, this method could allow the study of HDL structural dynamics. By aligning two docked PDB files, the differences between two structures in location and orientation suggested the equilibrium fluctuation and the structurally dynamic character of the HDL particle (**Figure 5C and 5F**, **Video S3**).

## Discussion

### 1. Image distortion limits high-resolution 3D reconstruction by conventional tomography reconstruction methods

The conventional method for ET reconstruction is to use the large micrograph-size images for more accurate determination of geometric tilt-angles and translation parameters [37], [38], [39], [40], [41], [42]. Unfortunately, the resolution of 3D reconstruction from these methods has rarely been better than 30 Å. It has been reported that image distortion limits reconstruction resolution [33]. As mentioned above, the image distortion could have numerous causes, such as defocus (details in subsection 3.1 of Methods Section), astigmatism [8], projector lens [34], pincushion and spiral [35], [36], energy filter [9], [10], and even radiation-induced deformations [11]. We suspected that image distortion prevents the accurate determination of tilt-angle, further limiting high-resolution reconstruction from large micrograph-size tilt images by conventional cryoET reconstruction. As described in subsection 3.1 of Methods Section, defocus-related distortion could result in ∼0.5° tilt-angle measuring error on a 4 k CCD under 20 kX magnification.

To demonstrate how tilt-error effects 3D reconstruction, we built up a large object that contains evenly distributed identical protein (**Figure S18**) and used it to project a set of 141 simulated micrograph-size noise-free images (∼4 k×4 k pixels) containing tilt-errors (both tilt-axis and tilt-angle errors) in a range of ±0.5° (**Figure S1**). By back-projecting the set of projection to 3D reconstruction, the 3D reconstruction contained evenly distributed particles (**Figure S18**), but, the quality of the particle reconstructions varied dramatically in term of the distance of a particle from the center of the full-size image (**Figure S18** and **S19A**). The particles near the center of the 3D reconstruction closely resembled the actual object, while those at the corners were least similar (**Figure S18** and **S19A**). To quantitatively evaluate the quality of each reconstructed particle against its spatial location, an FSC curve and CC C value between each reconstructed particle and object was computed. By plotting the *f_0.5_* and CC C values of the particles against their in-plane locations, both distributions showed a sharp peak at the center area, suggesting that only the particles/subvolumes near the center of reconstruction area were most similar to the object (**Figure S19B**).

To further examine the effects of smaller angle errors on a reconstruction, we repeated the above test using a smaller angle error (within a range of ±0.1° instead of ±0.5°). The particles near the central area were still the most similar to the object (**Figure S19C**), while the particles near the edges and corners consistently showed reduced similarity to the object (**Figure S19C**). *F_0.5_* and CC distributions had center peak areas significantly larger than those from the previous test (**Figure S19D**), suggesting a relatively larger area of high-resolution reconstruction. Both tests demonstrated that the geometric angle error grows with increasing displacement from the particle center and has uneven influence on the reconstruction quality, with the area near the reconstruction center having the highest reconstruction resolution.

The above results include the effect from both tilt-axis and tilt-angle errors. For a better understanding of the effect from tilt-axis error alone, we repeated the above test with only tilt-axis random errors in a range of ±0.5° (**Figure S20A**) and ±0.1° (**Figure S20B**). Both tests showed that the particles/subvolumes near the central area still had the best similarity to the object, while the particles/subvolumes near the corners consistently showed reduced similarity to the object based on *f_0.5_* and CC analyses. The distributions have a much narrower peak, but with a similar high correlation value under the larger tilt-axis errors (±0.5°) than under the smaller errors (±0.1°), suggesting that the central subvolumes/particles can tolerate a higher level of tilt-axis measurement error.

For a better understanding of the effect from tilt-angle error alone, we also repeated above tests with only the tilt-angle random errors in a range of ±0.5° (**Figure S21A**) and ±0.1° (**Figure S21B**). Both tests also showed that the particles/subvolumes along the tilt-axis had the best similarity to the object, while the particles/subvolumes far from the tilt-axis had the least similarity to the object based on *f_0.5_* and CC analyses. The distribution had a much narrower mountain ridge with a similar high correlation value in the larger tilt-angle errors test (±0.5°) than the smaller error test (±0.1°) (**Figure S21**), suggesting that the subvolumes/particles along the tilt-axis can tolerate a higher level of tilt-angle measurement error.

Although one of reasons to add the measurement errors of tilt-angle is the image distortion by different defocus values under non-parallel illumination condition, parallel illumination could be achieved by using a FEI Titan Krios microscope and a Zeiss Libra microscope, which could eliminate the defocus-related distortion. However, one cannot declare that there are no tilt-angle measurement errors for the tomographic data set collected from these microscopes.

Generally believed, the accurate tilt-axis orientation determination is very important for good ET reconstruction by conventional whole-micrograph tomography reconstruction methods. Among the three Euler angles in tomography reconstruction, the first Euler angle φ (particle angle) is equal to zero and not important. The offset value for the third Euler angle θ (tilt angle), which is important for the whole specimen section reconstruction by using ART [53] or the SIRT method [54], is also not important for conventional single-particle reconstruction because it only needs relevant angles. Does the second Euler angle ψ (tilt-axis angle) take effect with individual particle tomography? The tilt-axis errors include two types of error, the random error (we have discussed in above) and systemic error (because not all the microscopes could be pre-aligned well and will yield a none-zero tilt-axis angle within a range of ∼1°–5°). For a better understanding of the effect from the systemic angle-error of tilt-axis alone, we repeated the above test by only introducing a fixed systemic tilt-axis error of 1.0° (no any other errors included). By same analysis, the *f_0.5_* distribution showed a center peak (**Figure S22A**), suggesting that only the central subvolume had the highest similarity to the model, while the subvolumes along the tilt-axis direction are generally better than those against tilt-axis direction. The FSC curve (**Figure S22B**, blue line) between the model and the center subvolume showed the center subvolume (**Figure S22C**) retains its high similarity to the model with a resolution much better than 10 Å. By increasing the tilt-axis systemic error to 5.0° and even 10.0° respectively, the FSC curves (**Figure S22B**, purple and green lines) showed the center subvolume (**Figure S22D** and **S22E**) still retains its high similarity to the model at a resolution up to 10 Å. After low-pass filtering to 8 Å, all three central subvolumes are highly similar to each other in shape, but at a different tilt (**Figure S22C–S22E**). These tests suggest the tilt-axis orientation determination is very important for good ET reconstruction by conventional tomography reconstruction methods, but not important for our FETR algorithm. In other words, our FETR algorithm can tolerate a high tilt-axis systemic error (10°), while conventional whole-micrograph reconstruction cannot.

Whether the ∼0.5° angle measurement error is realistic or not, the existence of image distortion and tilt-error drove us to develop this FETR algorithm to tolerate these image distortions and measuring angle errors for high-resolution cryoET reconstruction.

### 2. Roughly 100 high-noise images are sufficient for a 3D reconstruction at intermediate resolution

In our cryoET reconstruction, a mere 100 high-noise images were sufficient to achieve intermediate resolution (1–2 nm); in contrast, single-particle reconstruction requires thousands of images to achieve the same resolution. As such, we asked whether ∼100 high-noise images are sufficient for a 3D reconstruction at an intermediate resolution. To address this question, we conducted the following simulation. We generated a set of 84 projections by rotating and projecting an object from a set of 84 geometric Euler angles generated from the single-particle reconstruction method under a space-sampling angle of 15° (“*VO EA*” command in SPIDER). Then, we added eight different levels of Gaussian noise with SNR ranging from noise-free to 0.1 to each set of projections (**Figure S23A, S23B, S23C**). This noise range covers the noise levels often present in cryoEM images [55], [56], [57]. To demonstrate the effect of noise level on 3D reconstruction, we back-projected eight reconstructions from each set of images with the assumption that all five parameters in each image were perfectly defined (**Figure S23D, S23E, S23F, S23G**). Although perfectly defining all parameters is impossible in practice, it is a necessary test to distinguish whether the resolution of a 3D reconstruction is limited by the noise level or other reasons. All eight density maps reconstructed from eight different noise levels (SNR = 0.1, 0.2, 0.3, 0.4, 0.6, 0.8, 1.0 and noise-free) were analyzed by two methods: FSC and CC analysis, which both analyses showed that the noise was reduced, and the reconstructions were similar to each other at a resolution close to or better than 10 Å (indicated by *f_0.5_* values) (**Figure S23H**). These structural details suggest that a set of 84 high-noise images (SNR = 0.1) would be sufficient for a reconstruction at intermediate resolution if all five parameters in each image can be perfectly defined.

Similar discussion about the minimum number of projections needed for a 3D reconstruction with a specific resolution has already been discussed by Crowther et al. [58], [59], [60]. Based on their equation, for the diameter of the molybdate transporter of ∼100 Å, the minimum number of projections for a reconstruction with ∼10 Å resolution is given by π×100 Å/10 Å = 31. Considering the calculation is based on noise-free projections, the minimum number of the noise-including projections should be higher than 31. The above simulation suggested that a set of 84 high-noise images (SNR = 0.1) would be sufficient for a reconstruction at intermediate resolution if all five parameters in each image can be perfectly defined. However, considering the accuracy of five parameters is critical plus the Gaussian noise has limitation in simulating the noise in cryoEM (discussed in subsection 1 of Methods Section), the minimum number of real experimental noise images is difficult to determine.

Another question posed is what results in real single-particle reconstruction often requiring thousands of particle images to achieve intermediate resolution [61]. We believe it is because the class-average process was used in single-particle reconstruction. To determine all five parameters, class-averaging procedure is often used to generate high-contrast class averages. With a typical single-particle reconstruction, a class average that contains ∼10–20 images and a sampling angle is usually between 5.0° and 10.0°, 173–711 class averages corresponds to a total of ∼1,730–14,220 raw particle images that are normally required for an asymmetric object. This number is consistent with an experimental image number in single-particle reconstruction.

In EM reconstruction, ∼100 high-noise images have never been used to generate 3D structure due to two reasons: the noise level is too high or the alignment is too poor. By excluding one possibility temporarily, our assumption that the alignment (five parameters) had been determined perfectly by an unknown method, ∼100 high-noise images with SNR = 0.1 can generate a 3D map at an intermediate reconstruction. The result showed that the high noise level (low SNR) is not the fundamental obstacle blocking achievement of high resolution. Thus, we believe the strategy of increasing SNR by high defocus value for cryoET data acquisition is not necessary in our IPET/FETR approach. Moreover, using high defocus value, CTF reversed amplitude more frequently results in more percentage of information permanently lost. In our real experiment, we chose low defocus for data acquisition. To determine how the five parameters under a high noise level, we expect other new strategies (such as tomography) can help us to either determine parameters precisely (tomography with markers) or limit the effect from measuring errors of parameters (such as our IPET method/FETR algorithm). In ET reconstruction, the three Euler angles can be naturally determined or estimated from the goniometer tilt angle or by tracking the movement of fiducial markers. Thus, only two translational parameters need to be defined in theory. Therefore, to achieve the same final reconstruction resolution, far fewer images are required for ET reconstruction compared with single-particle reconstruction because fewer parameters need to be determined. An additional feature of ET reconstruction is the images were all obtained from a single-instance object rather than from thousands of different objects. The intrinsic consistency among images can be more efficient to reduce the noise to achieve the same resolution using a less number of images in comparison to single-particle reconstruction by which the images from different objects may have conformational heterogeneity. A weakness of ET reconstruction is the effect of tilt limitation related missing wedge. Since the specimen holder can be tilted up to ∼70°, the tilt limitation leads to a wedge-shaped area of data absent during reconstruction. It is often believed that the effects from the missing wedge could be significant. However, we believe that the effect of this is limited with small and thin objects such as proteins (for details, see **Information** S1 and **Figure S24)**.

### 3. Resolution limitation of electron tomography

In the simulated cryoET images, the achieved resolution is better than 10 Å. However, the same resolution cannot be guaranteed to be obtained from the real cryoET data. Considering that the main purpose of the simulated cryoET data is to demonstrate the IPET method/FETR algorithm, the parameters/conditions applied in the simulated data, such as informational/signal completeness, defocus and SNR, are all “perfect”. However the parameters in the real cryoET data are not perfect due to all of the challenges involved in cryoET data acquisition. Taking the signal/informational completeness as an example, in the antibody NS images SNR is much higher than in the simulated cryoET images, and the reconstruction resolution (∼14–16 Å) measured using the intra-*f_0.5_* method is worse than is achieved in the simulated cryoET images. Regardless, the intra-*f_0.5_-*defined resolution is usually poorer than the real resolution (discussed in subsection 2 of Results Section, **Figure 3B**), and lower resolution is most likely due to more incomplete structural information of the real object resulted by using a higher defocus value with a larger defocus variation range (defocus varied in the range of ∼1.0–2.0 µm during titling) in NS imaging. The higher defocus can eliminate more structural detail because the amplitude of CTF more frequently crosses zero. The larger defocus variation range could result in less percentage of common structural details that were used for translational parameter searching. Thus, the “imperfect” NS images in terms of inconsistent structural details caused by different defocus could contribute to more error in translational parameter searching. Nevertheless, considering the highest NS-EM resolution from any EM technique (including single-particle, 2D crystallography and tomography) is rarely better than 12 Å [62], the resolution of our NS-EM reconstruction of an antibody is likely limited by the experimental condition itself, rather than the capability of FETR algorithm. However, in the real HDL cryoET images in which the proteins were imaged in near-native state and contained no interference from NS, the resolution defined by intra-f_0.5_ is usually worse than the real resolution (discussed in subsection 2 of Results Section, **Figure 3B**), the reconstruction resolution (∼36 Å) is also worse than achieved by simulated cryoET images. We believe that the lower resolution is due to the challenges in collecting the real cryoET experimental data under the “perfect” parameters. The parameters in real cryoET images, such as defocus value, SNR and radiation damage, cannot be as well controlled as those used in the simulated cryoET images. Taking the defocus parameter as an example, the defocus values are all the same as in the simulation cryoET images, but not in the real cryoET experimental images. Measuring the defocus of each tilt image is rather challenging because of the low SNR in each image that is acquired under a low-dose condition (<∼3 e^−^/Å ^2^), and controlling defocus is also rather challenging because defocus is significantly related to the mechanical height (Z-height) and drift of specimen holder, the tilt-axis stability in terms of the distance to the image center, the distance between the targeted particle and the tilt-axis, and the flatness of the sample supporting substrate. However, by further optimizing the experimental operation strategy, the defocus variation can be minimized. The optimizing strategy includes only targeting the particles that are located only near the tilt-axis. Those particles that have a relatively smaller variation of defocus change during tilting. By manually adjusting the defocus value of each tilt image following a method commonly used in 2D crystal cryoEM data acquisition [55], [56], the defocus variation can be minimized. A brief description of this method is that the defocus value of the targeted area (close to tilt-axis) could be calculated based on the average defocus of two high-dose imaged areas that share the same tilt-axis within the targeted area, but are opposite distances away from the central targeted area in some distance (such as 1–2 µm at the magnification of 50 kX). Taking the SNR parameter as an example, SNRs are all the same in the simulation cryoET images, but not in the real experimental images. Although, theoretically, the SNR should be a constant number when following the “exponential” exposure time scheme on a constant ice thickness area [23], in practical terms, the same thickness of ice is difficult to achieve, resulting in the variation of SNR of real cryoET images under different tilts. However, by further optimizing the cryoEM sample preparation method, the SNR variation can be minimized. For example, our experience is that the variation in ice thickness supported by a relatively large-hole (>5 µm in diameter) is relatively small. The large-hole in the home-made holey thin carbon film can provide a relatively flat and ultra-thin ice (<∼700 Å) that can be used to reduce the variation of ice thickness/SNR and to provide a slightly better contrast image. From experience, an additional benefit of using a larger-size ice is that the larger ice can tolerate relatively higher doses before observation of bubbling. For the radiation damage parameter, there is no radiation damage in the simulated cryoET data, but a certain level of radiation damage that must exist in the real cryoET image could also affect the reconstruction resolution. In short, the “perfect” parameters are challenging to be obtained or controlled in the real cryoET data acquisition, and this is what limits the reconstruction resolution rather than being limited by our FETR algorithm.

Our motivation in developing this IPET method/FETR algorithm is that we believe that a resolution beyond ∼20 Å is possible to be obtained. The 20 Å resolution limitation of cryoET has been frequently quoted based on a theoretical calculation [63]. However, one should notice two key parameters/assumptions used in this calculation, i.e. the total dose of 5–20 e^−^/Å ^2^ and the solvent contrast factor of 0.28. Total tolerable dose for cryoET is a key parameter to determining the final resolution [23]. The dose limitation of cryoEM has been discussed for decades [64], [65], [66], [67], [68], [69], [70], [71], and it is generally believed that a conventional dose limitation is 5–20 e^−^/Å ^2^ to obtain an atomic resolution level [55], [56], [64], [72]. To target a low to intermediate resolution reconstruction, the tolerated dose can usually be 2–5 times higher than the conventional dose [64], [67], [71], and an even higher dose (50–150 e^−^/Å ^2^) has been frequently used in real cryoET data acquisition [73], [74]. The total tolerated dose also differs from specimen to specimen [23]. In our HDL cryoET images [15], the total dose is ∼140 e^−^/Å ^2^ (7–30 times higher than the conventional dose limitation), in which we did not observe bubbling in the ice-crossed-hole area. Given a large dose (up to 120 e^−^/Å ^2^) and an ultra-thin ice (<1,000 Å) condition, a 10–20 Å resolution could be achieved in theory, by cryoET under the ideal imaging conditions reported by Baumeister et al. [23]. This resolution is consistent to the Rosenthal and Henderson's calculation by assuming a total dose of 120 e^−^/Å ^2^ [63]. The contrast factor of 0.28 is another key parameter in defining the 20 Å resolution limitation [23]. Considering the contrast factor of 0.28 is for x-ray scattering instead of electron scattering [63], while the contrast factor for neutral scattering is 0.42 [63], the contrast factor for electron scattering should be within the range of 0.28–0.42 because the electron scattering factors are higher than x-ray scattering factors [75], [76], [77], [78]. A higher contrast factor (>0.28) should result in a resolution limitation higher than 20 Å using the same equation reported by Rosenthal and Henderson [63]. Thus, we believe a better than 20 Å resolution cryoET reconstruction can reasonably be expected.

Whether the resolution obtained from the simulated cryoET image is realistic or not, the major focus of this report is to demonstrate a cryoET reconstruction methodology that can tolerate the tilt-errors, which conventional methods cannot.

### Conclusion

We have proposed a focused ET reconstruction (FETR) method that allows us to determine the structure of a single instance of a protein at intermediate resolution. This method could be used as a novel approach to study equilibrium fluctuations and dynamic characteristics of protein via a comparison of “snapshot” structures from different objects of proteins.

## Supporting Information

Figure S1 **Diagram of the geometric angles (tilt-angle) in electron tomography reconstruction.** The tilt axis was pre-aligned parallel to the Y-axis of CCD frame. We assumed the “measured” tilt-axis, ψ = 0, which will be used for reconstruction during the iteration. However, this “measured” tilt-axis must contain an angle-error, i.e., Δψ. Thus, the “real” tilt axis should be equal to Δψ. Similarly, the tilt-angle, called “measured” tilt angle, is θ, and the angle-error of tilt angle is Δθ.
(TIF)

Figure S2 **Real nascent HDL particles imaged by cryo-electron tomography (CryoET).** Selected tilted views of nascent apoA-I/HDL particles embedded in vitreous physiological buffer and imaged by cryo-electron tomography. In each view, the axis of tilt is vertical to the images. Selected titled images are linked by dotted arrows, while relative tilt angles are indicated in each image. Scale bars, 200 Å. (This research was originally published in the Journal of Biological Chemistry. Jones MK, Zhang L, Catte A, Li L, Oda MN, et al. Assessment of the validity of the double superhelix model for reconstituted high density lipoproteins: a combined computational-experimental approach. *J. Biol. Chem.* 2010; 285: 41161–41171. © the American Society for Biochemistry and Molecular Biology).
(TIF)

Figure S3 **Defocus-introduced image distortion.** To quantitatively demonstrate the change in magnification resulting from change in defocus, 70 particles of the 5 nm nanogolds were tracked from the micrographs that were taken under the defocus changes from 0.0 µm to 10 µm in steps of 0.5 µm. The particle coordinates were aligned to each other and fitted with a second-degree polynomial function, i.e., *u = a_0_+a_1_x+a_2_y+a_3_x^2^+a_4_y^2^+a_5_xy, v = b_0_+b_1_x+b_2_y+b_3_x^2^+b_4_y^2^+b_5_xy*, by Matlab. The analysis showed that defocus could result in a near-linear change of magnification, i.e., other than *a_1_* and *b_2_*, all other parameters are close to 0. Thus, a near linear change of ∼8% of magnification could be introduced as the defocus changed by 10 µm under a non-parallel-beam EM operation condition.
(TIF)

Figure S4
**Equivalent tilt-angle error caused by defocus-induced distortion.** Given the tilt-axis is along image central Y-axis and a defocus-induced shrink ratio (magnification change divided by defocus change) is *S* (here *S* = 8%/10 µm = 0.008 µm^−1^), for an ideal projection at position (*x, y*) in the micrograph/projection plane, the defocus is 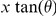 and the object distance between the real object to the tilt-axis in specimen plane is 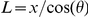. Since the defocus-introduced shrinkage is along *x*-axis, the *x*-axis coordinate of the observed projection (after shrinkage) is 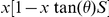. Since the object distance *L* is same during measuring the tilt-angle, a measured tilt-angle *θ*+Δ*θ* should satisfy to the distance *L* constraint, i.e. 
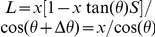
. Thus, 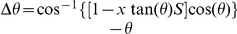. Considering Δ*θ* is usually small (<∼1–2°), the equation can be simplified as 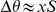. As noticed, the different *x*-coordinates of the object can generate different tilt-angle errors and the tilt-angle error is independent of tilt-angle *θ*. Considering the maximum *x* is half of micrograph size, i.e. the maximum Δ*θ* can be expressed as 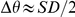, where *D* is the full image size in µm. For a 4 k CCD image at a magnification of 15 kX (7.76 Å/pixel), the maximal tilt-angle error Δ*θ* is 0.73°; for a magnification of 20 kX (5.6 Å/pix), the maximal Δ*θ* is 0.53°; for a magnification of 50 kX (2.25 Å/pix), the maximal Δ*θ* is 0.21°; and for a magnification of 80 kX (1.40 Å/pix), the maximal Δ*θ* is 0.13°.(TIF)

Figure S5 **FSC analyses of the 3D reconstructions from the simulated cryoET images by FETR.** (A) Intra-FSC curves were calculated based on separate reconstructions from two halves of the tilt series initially (gray circle-dash line), after round one (green diamond-dash line), round two (purple cross-dash line), and round three (blue point-dash line). The curves showed the 3D reconstructions improved significantly after the refinement with iterations. (B) FSC curves were calculated between the object and the 3D reconstructions of the initial model (gray circle-dash line), round one (green diamond-dash line), round two (purple cross-dash line), and round three (blue point-dash line). The curves showed the 3D reconstructions continually improved after the first round.
(TIF)

Figure S6 **Monitoring the particle-shaped masks in FETR.** A total of six automatically generated particle-shaped masks (A–L) were used to further reduce the noise and unnecessary background in the second round of iterations. To confirm that the signal of the targeted particle has not been eliminated or truncated, the masks had to be monitored during the iterations. The selected masked particles showed that no obvious portions of the particle were truncated by using these masks.
(TIF)

Figure S7 **3D reconstruction of first single-instance of IgG antibody by IPET/FETR.** The antibody sample was prepared by an optimized NS protocol [45], [46] and imaged by ET. Selected slice views of 3D reconstruction before applying particle-shaped masks (A) before applying low-pass filters and (B) after applying low-pass filters; after the particle-shaped masks were applied, the slice views were shown (C) before applying low-pass filters and (D) after applying low-pass filter. (E) Selected tilted view of the 3D density map displayed at a high-contour level, (F) a low-contour level before applying the mask, and (G) the 3D density map after applying the mask.
(TIF)

Figure S8 **3D reconstruction of second single-instance of IgG antibody by IPET/FETR.** The antibody sample was prepared by an optimized NS protocol [45], [46] and imaged by ET. Selected slice views of 3D reconstruction before applying particle-shaped masks (A) before applying low-pass filters and (B) after applying low-pass filters; after the particle-shaped masks were applied, the slice views were shown (C) before applying low-pass filters and (D) after applying low-pass filter. (E) Selected tilted view of the 3D density map displayed at a high-contour level, (F) a low-contour level before applying the mask, and (G) the 3D density map after applying the mask.
(TIF)

Figure S9 **The intra-FSC analyses of two IgG antibody density maps reconstructed by IPET/FETR.** By intra-*f_0.5_* criterion, the intra-FSC showed that the resolution achieved by FETR are (A) ∼14.1 Å for antibody #1 and (B) ∼14.6 Å for antibody #2 respectively.
(TIF)

Figure S10 **Monitoring the particle-shaped masks in two IgG antibodies reconstruction by IPET/FETR.** During the second round of iterations, a total of six automatically generated particle-shaped masks were used to further reduce the noise and unnecessary background while generating the 3D reconstructions of two IgG antibodies. To confirm that the signal of the targeted particle was not been eliminated or truncated, the masks were monitored during the iterations. The selected masked particles showed that no obvious portions of the particle were truncated by using these masks for antibody number one (A) and two (B).
(TIF)

Figure S11 **The crystal structure of IgG antibody (PDB entry 1IGT) displays a hole within each domain.** (A) By displaying the crystal structure in ribbon, and (B) van der Waals surface, the holes were displayed clearly within the F_ab_ domains. By 90° rotation along the Y-axis, both (C) the ribbon and (D) van der Waals surface images display an even bigger hole within the F_c_ domain, suggesting the hole in each domain is the intrinsic structure feature in the IgG antibody.
(TIF)

Figure S12 **Human IgG antibody particles prepared by optimized negative-staining protocol and imaged at near Scherzer focus.** (A) Survey view of human IgG antibody imaged. The white-circled particles clearly displayed three domains within each particle. (B) Selected three particles display low-density regions (holes indicated by dash arrows) within domains. (C) Their corresponding orientations of the crystal structure (PDB entry 1IGT) displayed in their corresponding holes within the corresponding domains can also be visualized, suggesting the holes are the intrinsic structure features instead of the artifact from neither negative-staining nor defocus-related contrast transfer function (CTF).
(TIF)

Figure S13 **3D reconstruction of first single-instance of nascent HDL particle from the cryoET images by IPET method.** (A) Selected slice views of 3D reconstruction before applying low-pass filtering and particle-shaped masks; and (B) their corresponding slice views of 3D reconstruction after low-pass filtering, but before masking; and (C) the corresponding slice views before filtering, but after masking; and (D) views after both filtering and masking. (E) Selected tilted view of the 3D density map before applying the mask, and (F) the corresponding views of 3D density map after applying the mask, showing only few isolated small densities (in gray in E) were truncated by the mask.
(TIF)

Figure S14 **3D reconstruction of second single-instance of nascent HDL particle from the cryoET images by IPET method.** (A) Selected slice views of 3D reconstruction before applying low-pass filtering and particle-shaped masks; and (B) their corresponding slice views of 3D reconstruction after low-pass filtering, but before masking; and (C) the corresponding slice views before filtering, but after masking; and (D) views after both filtering and masking. (E) Selected tilted view of the 3D density map before applying the mask, and (F) the corresponding views of 3D density map after applying the mask.
(TIF)

Figure S15 **The intra-FSC analyses of two nascent HDL density maps reconstructed by IPET method.** By the intra-*f_0.5_* criterion, intra-FSC curves showed that the resolution achieved by FETR algorithm are (A) ∼42.5 Å for nascent HDL particle #1 and (B) ∼36.1 Å for HDL particle #2.
(TIF)

Figure S16 **Monitoring the particle-shaped masks in the nascent HDL reconstruction by IPET method.** To confirm that the signal of the targeted nascent HDL particle has not been eliminated or truncated during the second round of iterations, where a set particle-shaped masks were applied on the raw particle images to eliminate the noise contribution to the translational searching. The masks were monitored during the iterations. Six masks and masked particles were displayed, suggesting that no obvious portions of the particle were truncated in the IPET method for 3D reconstruction of nascent HDL number one (A) and two (B).
(TIF)

Figure S17 **Discoidal shape of 17 nm nascent HDL particle reported by the conventional cryoEM imaged from two orthogonal tilt views.** (A) van Antwerpen *et al.* investigated the 17 nm HDL particle shape by cryoEM. HDL particles were embedded in vitreous ice and imaged from two orthogonal tilt-viewing angles. Four selected particles (top panel) are represented with rod-shape (B), while their corresponding 90° tilted views present a circular shape (C). Thus, van Antwerpen *et al.* proposed a discoidal shape model for 17 nm HDL particles. (This research was originally published in Journal of Lipid Research. van Antwerpen R, Chen GC, Pullinger CR, Kane JP, LaBelle M, et al. Cryo-electron microscopy of low density lipoprotein and reconstituted discoidal high density lipoprotein: imaging of the apolipoprotein moiety. *J. Lipid Res.* 1997; 38: 659–669. © the American Society for Biochemistry and Molecular Biology).
(TIF)

Figure S18 **3D reconstruction from a whole-micrograph-size tilt images that contain the tilt-errors.** To demonstrate the effect of tilt-errors on the 3D reconstruction, we back-projected a set of 141 simulated micrograph-size noise-free images (4120×4120 pixels) containing tilt-axis and/or tilt-angle errors in a defined range such as ±0.5°. The particle-density maps (subvolumes) were windowed from different spatial location from the large micrograph reconstruction (4120×4120×160 voxels).
(TIF)

Figure S19 **Effect of tilt-error (including both tilt-axis and tilt-angle errors) in whole-micrograph-size reconstruction**. (A) To demonstrate the effects of tilt-error in the 3D reconstruction, both tilt-axis and tilt-angle were introduced with a random error within a range of ±0.5°. The particle density maps (subvolumes) were windowed from different spatial locations from the large-micrograph reconstruction (4120×4120×160 voxels, **Figure S18**). The quality of the 3D reconstructions of the objects were dependent on the positions of the objects. The selected particles/subvolumes showed that the reconstruction from the center contains more similarity to that from the edge. (B) To quantitatively evaluate the quality of each reconstructed particle against its spatial location, a Fourier shell correlation (FSC) curve and cross-correlation coefficient (CC C) value between each reconstructed particle and object was computed as shown. By plotting the *f_0.5_* (left) and CC C (right) values of the particles against their in-plane locations, the topography of the *f_0.5_* and CC C showed a peak near the center of the specimen, suggesting the highest quality of reconstruction was at the center. (C) To demonstrate the effect of ±0.1° tilt-errors on the 3D reconstruction, the particle-density maps were windowed from different spatial location from the large micrograph reconstruction. The selected particles/subvolumes showed that the reconstruction from the center contains the highest quality. (D) By plotting the *f_0.5_* (left) and CC C (right) values of the particles against their in-plane locations, the topography of the *f_0.5_* and CC C showed a peak near the center of the specimen, suggesting the highest quality of reconstruction was at the center. Both distributions showed a sharp peak at the center area, suggesting that only the particles near the center of reconstruction area had the highest degree of similarity to the object, and further suggesting the center subvolume can tolerate high degree of tilt-error.
(TIF)

Figure S20 **Effect of random tilt-axis error in the whole-micrograph-size reconstruction.** To better understand the effect from random error of tilt-axis, we repeated above test by tilt-axis only containing a random errors in a range of (A) ±0.5° and (B) ±0.1°. The whole-micrograph reconstruction was analyzed by comparing each subvolume to the object to compute the FSC curves and CC values. The topographies of the *f_0.5_* (left) and CC value (right) were displayed against their position in micrograph. Both tests showed that the particles/subvolumes near the micrograph central area still retained their best similarities to the object, while the particles/subvolumes near the corners consistently retained their least similarities to the object based on *f_0.5_* and CC analyses. The distribution had a much narrow peak, but with similar height, in the larger tilt-axis errors (±0.5°) than the smaller errors (±0.1°), suggesting that the reconstruction near image center can tolerate a higher level of tilt-axis measurement error.
(TIF)

Figure S21 **Effect of tilt-angle error in the whole-micrograph-size reconstruction.** Containing only the tilt-angle random errors in a range of (A) ±0.5° and (B) ±0.1°, the whole-micrograph reconstruction was analyzed by comparing each subvolume to the object for computing the FSC curves and CC values. The topographies of the *f_0.5_* (left) and CC value (right) were displayed against their position in micrograph. Both analyses showed that the particles/subvolumes near the tilt-axis area have the best similarity to the object, while the particles/subvolumes far from tilt-axis area have the least similarity to the object. The distribution had a much narrow ridge, but with similar height in the larger tilt-angle errors (±0.5°) than the smaller error (±0.1°), suggesting that the reconstruction near tilt-axis can tolerate a higher level of tilt-angle measurement error.
(TIF)

Figure S22 **Effect of systematic tilt-axis error in the whole-micrograph-size reconstruction.** A similar test was repeated by only introducing a fixed systemic tilt-axis error of 1.0° (no any other error). (A) The *f_0.5_* distribution showed a center peak, suggesting the central subvolume retained its highest similarity to the model. The subvolumes near the tilt-axis are generally better than that far from tilt-axis. (B) By increasing the systemic tilt-axis error from 1.0° to 5.0° and 10.0°, the FSC curves calculated between the object and each central subvolume displayed the center subvolume retains its similarity to the object up to resolution of 10 Å. (C–E) After it was low-pass filtered for each central subvolume up to 8 Å, the three subvolumes displayed near identical similarity, except tilting, suggesting the central subvolume can tolerate a relative large tilt-axis symmetric error.
(TIF)

Figure S23 **Noise effects in the single-particle 3D reconstructions.** (A) 84 projections were generated by projecting the object based on a set of single-particle Euler angles, i.e. a sampling angle of 15°. Five represented projections were displayed. By adding eight different levels of noise to the projections, such as (B) SNR = 0.2 and (C) SNR = 0.1 that are similar to the noise level presented in cryoEM images, we back-projected each set of noisy images by following same Euler angles as for projection. The 3D reconstructions were then low-pass filtered to 8 Å. Three 3D reconstructions, (D) noise-free, (E) SNR = 0.2, and (F) SNR = 0.1 were displayed, in which, (G) the 3D reconstruction of SNR = 0.1 was docked with the model of crystal structure. The reconstructions showed high similarity to each other in the term of the detailed structure, such as α-helices. (H) Using the quantitative analyses of the 3D reconstructions, the FSC curves were computed by comparing each 3D reconstruction to the object. The FSC curves showed that all *f_0.5_* values were close to or beyond 1/10 Å^−1^. (I) By the real space analyses, the cross-correlation coefficients (CC C) between the object and each reconstruction showed a similar trend of *f_0.5_* values.
(TIF)

Figure S24 **Missing-wedge effect in the 3D reconstruction of a thin and small protein.** Three density maps, named single-particle map, ET map, and “ideal” ET map, were back-projected from three sets of 84 images that were projected from the same object, but using different sets of projection angles. (A) The single-particle map was reconstructed from single-particle projections based on a set of single-particle Euler angles (sampling angle of 15°). (B) The ET map was reconstructed from the ET projections and based on a set of ET Euler angles, tilt-angles were evenly distributed in the range from −70° to +70°. (C) The “ideal” ET map was same as the ET map, except the tilt-angles were evenly distributed in a range from −90° to +90°. After they were low-pass filtered to 8 Å, these three maps displayed no obvious differences. (D) To quantitatively analyze the quality of each map, FSC curves between the object and each map were calculated and plotted. All three curves were above 0.5 at the nyquist frequency (0.5 Å^−1^). In the relatively low-resolution zone (<∼0.28 Å^−1^), FSC curves showed the ET map had lower or reduced similarity to the object than single-particle map. However, in the relatively high-resolution zone (>∼0.28 Å^−1^), FSC analyses showed the ET map had increased similarity to the object than single-particle map. Overall, the “ideal” ET map remains as the best quality at any frequency. (E) Quantitative analyses of the quality of three maps were also performed in real space. By calculating the cross-correlation coefficient (CC C) between each map to the object, the “ideal” ET map retains its most similarity to the object, while the single-particle and ET maps have about equal similarity to the object, but EM map is slightly better than the single-particle map.
(TIF)

Table S1 **Defocus-introduced image distortion.** Tracking the movements of 70 particles (5 nm nanogold particles) that were imaged under the defocus changes from 0.0 µm to 10 µm in steps of 0.5 µm was used for quantitative determination of the defocus-introduced distortion under a non-parallel beam conditions. The coordinates of each particle imaged under the different defocus were fitted into a 2^nd^ degree polynomial function, i.e. *u = a_0_+a_1_x+a_2_y+a_3_x^2^+a_4_y^2^+a_5_xy, v = b_0_+b_1_x+b_2_y+b_3_x^2^+b_4_y^2^+b_5_xy*. The fitting parameters were defined by a least squares fitting method using Matlab.
(DOC)

Video S1 **Image distortion/magnification change introduced by defocus change under a non-parallel beam condition.** To quantitatively measure the magnification change against the defocus change, 70 particles (5 nm nanogold) were imaged under defocus from 0.0 µm to 10 µm in steps of 0.5 µm. By aligning the micrographs together based on their cross correlation coefficient, the aligned images were then combined into a movie to display the particles shrinking to the micrograph center against the defocus increasing.
(MPEG)

Video S2 **Equilibrium fluctuation of IgG antibody.** The structural difference between two single-instances of IgG antibodies is a way to evaluate the structural flexibility and equilibrium fluctuation. By aligning the two docked antibody PDB files by their F_c_ domains, the trajectory that morphs between them is created and shown in a video, suggesting the equilibrium fluctuation and structural dynamic character of antibodies.
(MPG)

Video S3 **Equilibrium fluctuation of nascent HDL.** Comparing the structures between two single-instances of nascent HDL particles is a way to evaluate structural flexibility and equilibrium fluctuation. By aligning two docked HDL PDB files, the trajectory between two docked structures shows the equilibrium fluctuation of containing apolipoproteins in nascent HDL.
(MPG)

Information S1 **Effect of missing wedge on 3D reconstruction is limited to high-resolution ET reconstruction of small and thin objects.**
(DOC)
