# Supplementary figures and images for: IPET and FETR: Experimental Approach for Studying Molecular Structure Dynamics by Cryo-Electron Tomography of a Single-Molecule Structure
Source: PLoS One. 2012 Jan 24;7(1):e30249. doi: 10.1371/journal.pone.0030249 (PMC3265479; doi:10.1371/journal.pone.0030249)

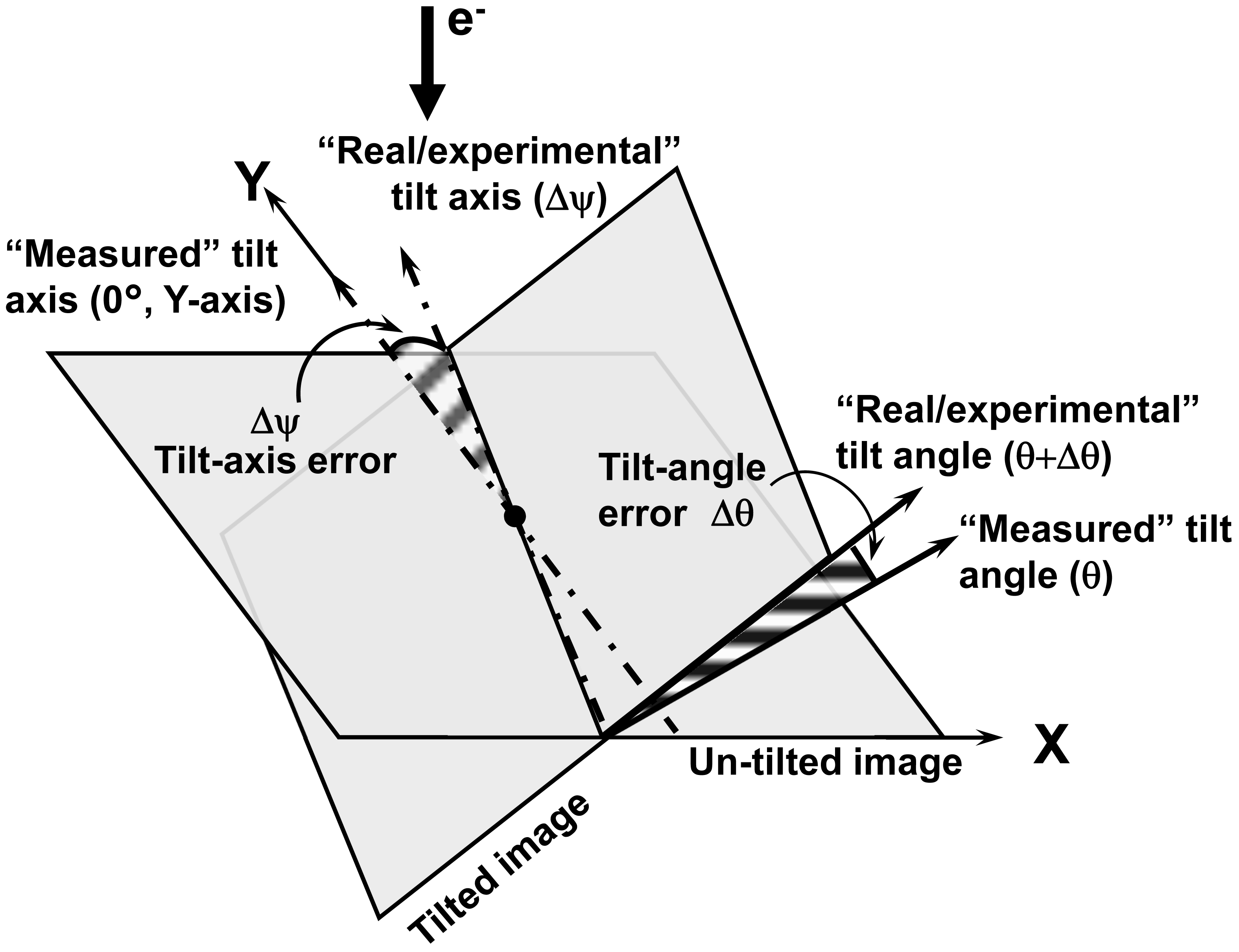

Supplement: Figure S1 — Diagram of the geometric angles (tilt-angle) in electron tomography reconstruction. The tilt axis was pre-aligned parallel to the Y-axis of CCD frame. We assumed the “measured” tilt-axis, ψ = 0, which will be used for reconstruction during the iteration. However, this “measured” tilt-axis must contain an angle-error, i.e., Δψ. Thus, the “real” tilt axis should be equal to Δψ. Similarly, the tilt-angle, called “measured” tilt angle, is θ, and the angle-error of tilt angle is Δθ. (TIF) [file pone.0030249.s001.tif]

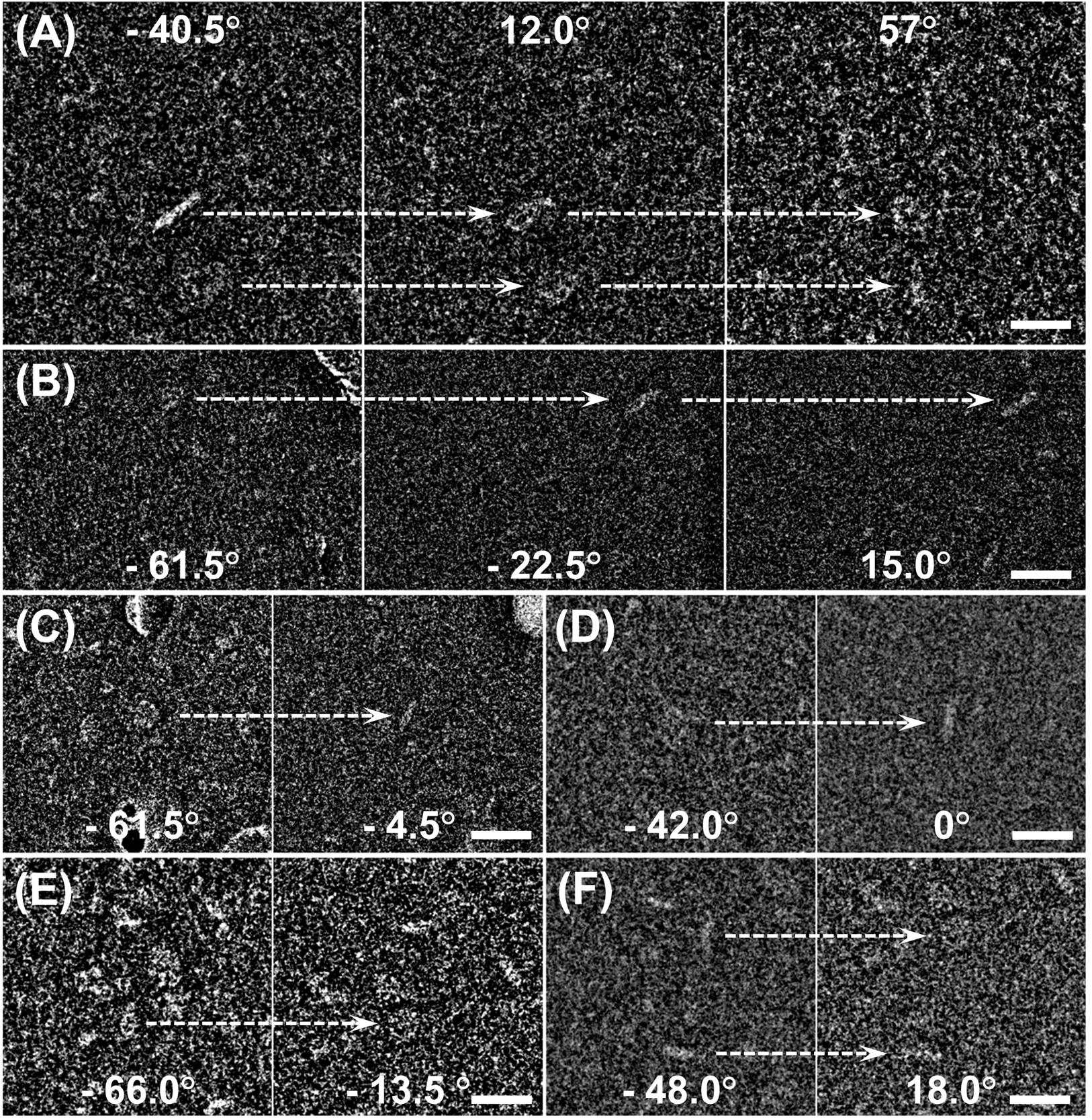

Supplement: Figure S2 — Real nascent HDL particles imaged by cryo-electron tomography (CryoET). Selected tilted views of nascent apoA-I/HDL particles embedded in vitreous physiological buffer and imaged by cryo-electron tomography. In each view, the axis of tilt is vertical to the images. Selected titled images are linked by dotted arrows, while relative tilt angles are indicated in each image. Scale bars, 200 Å. (This research was originally published in the Journal of Biological Chemistry. Jones MK, Zhang L, Catte A, Li L, Oda MN, et al. Assessment of the validity of the double superhelix model for reconstituted high density lipoproteins: a combined computational-experimental approach. J. Biol. Chem. 2010; 285: 41161–41171. © the American Society for Biochemistry and Molecular Biology). (TIF) [file pone.0030249.s002.tif]

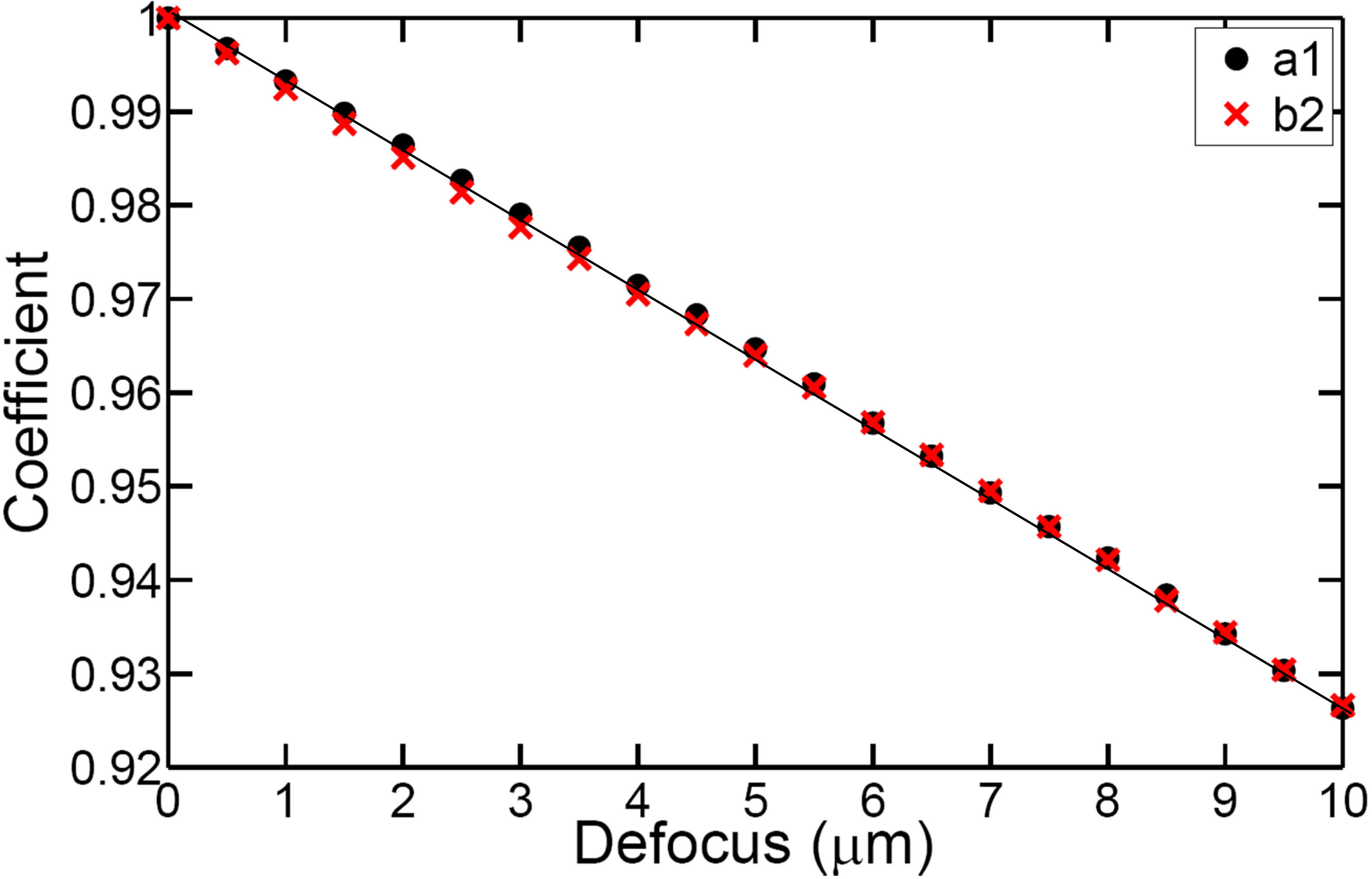

Supplement: Figure S3 — Defocus-introduced image distortion. To quantitatively demonstrate the change in magnification resulting from change in defocus, 70 particles of the 5 nm nanogolds were tracked from the micrographs that were taken under the defocus changes from 0.0 µm to 10 µm in steps of 0.5 µm. The particle coordinates were aligned to each other and fitted with a second-degree polynomial function, i.e., u = a0+a1x+a2y+a3x2+a4y2+a5xy, v = b0+b1x+b2y+b3x2+b4y2+b5xy, by Matlab. The analysis showed that defocus could result in a near-linear change of magnification, i.e., other than a1 and b2, all other parameters are close to 0. Thus, a near linear change of ∼8% of magnification could be introduced as the defocus changed by 10 µm under a non-parallel-beam EM operation condition. (TIF) [file pone.0030249.s003.tif]

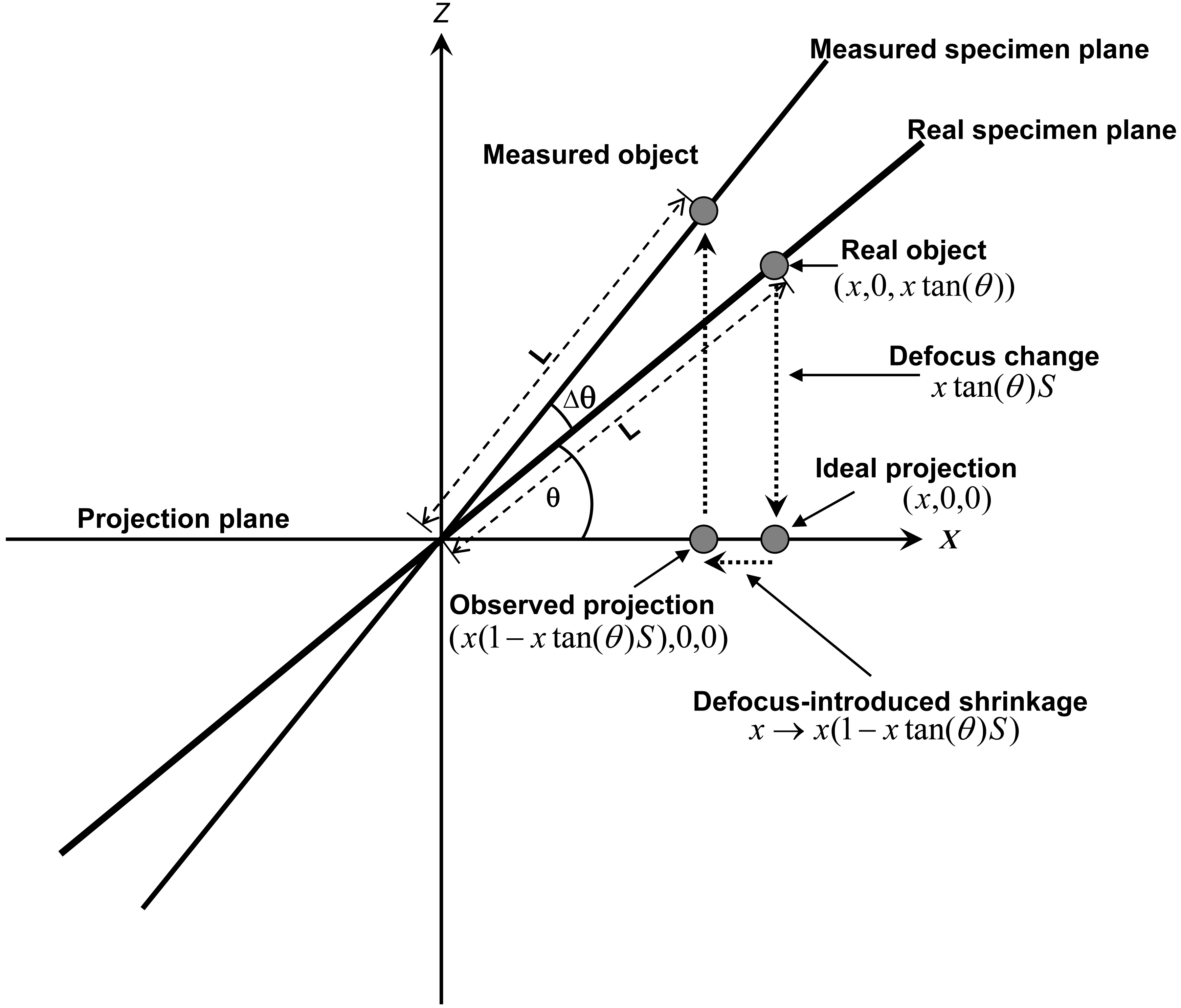

Supplement: Figure S4 — Equivalent tilt-angle error caused by defocus-induced distortion. Given the tilt-axis is along image central Y-axis and a defocus-induced shrink ratio (magnification change divided by defocus change) is S (here S = 8%/10 µm = 0.008 µm−1), for an ideal projection at position (x, y) in the micrograph/projection plane, the defocus is and the object distance between the real object to the tilt-axis in specimen plane is . Since the defocus-introduced shrinkage is along x-axis, the x-axis coordinate of the observed projection (after shrinkage) is . Since the object distance L is same during measuring the tilt-angle, a measured tilt-angle θ+Δθ should satisfy to the distance L constraint, i.e. . Thus, . Considering Δθ is usually small (<∼1–2°), the equation can be simplified as . As noticed, the different x-coordinates of the object can generate different tilt-angle errors and the tilt-angle error is independent of tilt-angle θ. Considering the maximum x is half of micrograph size, i.e. the maximum Δθ can be expressed as , where D is the full image size in µm. For a 4 k CCD image at a magnification of 15 kX (7.76 Å/pixel), the maximal tilt-angle error Δθ is 0.73°; for a magnification of 20 kX (5.6 Å/pix), the maximal Δθ is 0.53°; for a magnification of 50 kX (2.25 Å/pix), the maximal Δθ is 0.21°; and for a magnification of 80 kX (1.40 Å/pix), the maximal Δθ is 0.13°. (TIF) [file pone.0030249.s004.tif]

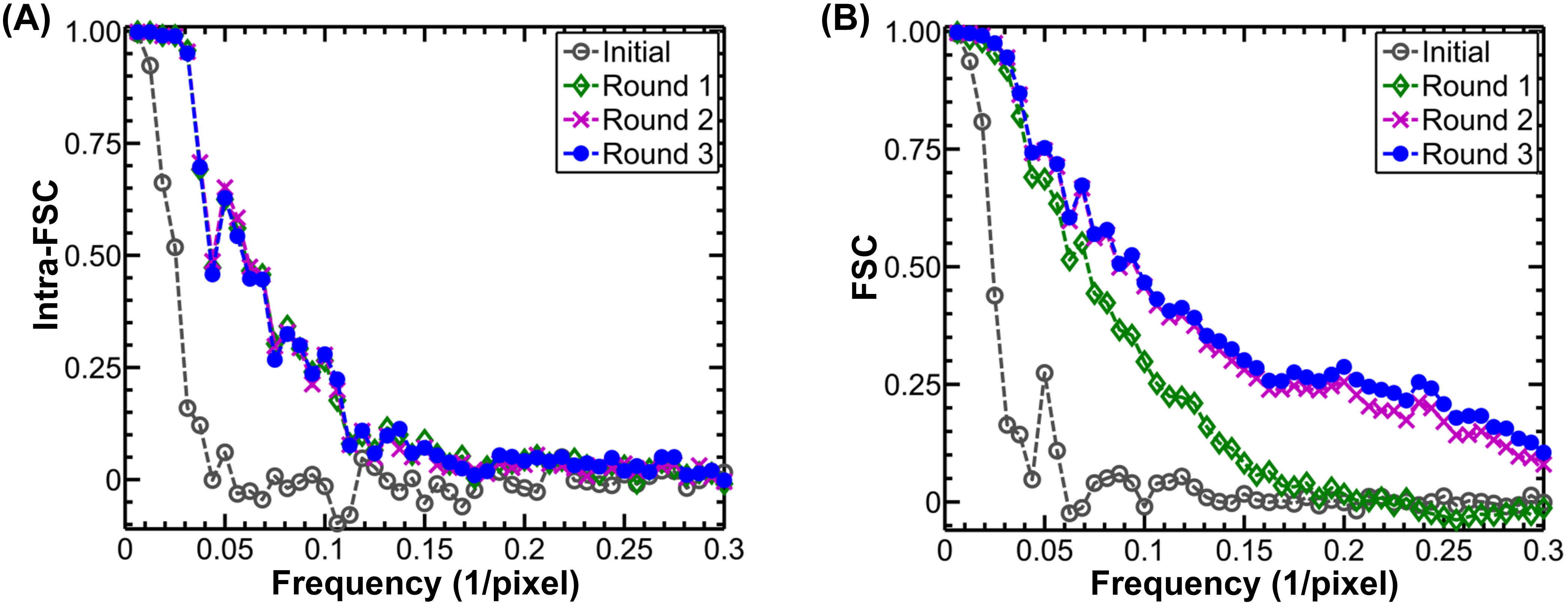

Supplement: Figure S5 — FSC analyses of the 3D reconstructions from the simulated cryoET images by FETR. (A) Intra-FSC curves were calculated based on separate reconstructions from two halves of the tilt series initially (gray circle-dash line), after round one (green diamond-dash line), round two (purple cross-dash line), and round three (blue point-dash line). The curves showed the 3D reconstructions improved significantly after the refinement with iterations. (B) FSC curves were calculated between the object and the 3D reconstructions of the initial model (gray circle-dash line), round one (green diamond-dash line), round two (purple cross-dash line), and round three (blue point-dash line). The curves showed the 3D reconstructions continually improved after the first round. (TIF) [file pone.0030249.s005.tif]

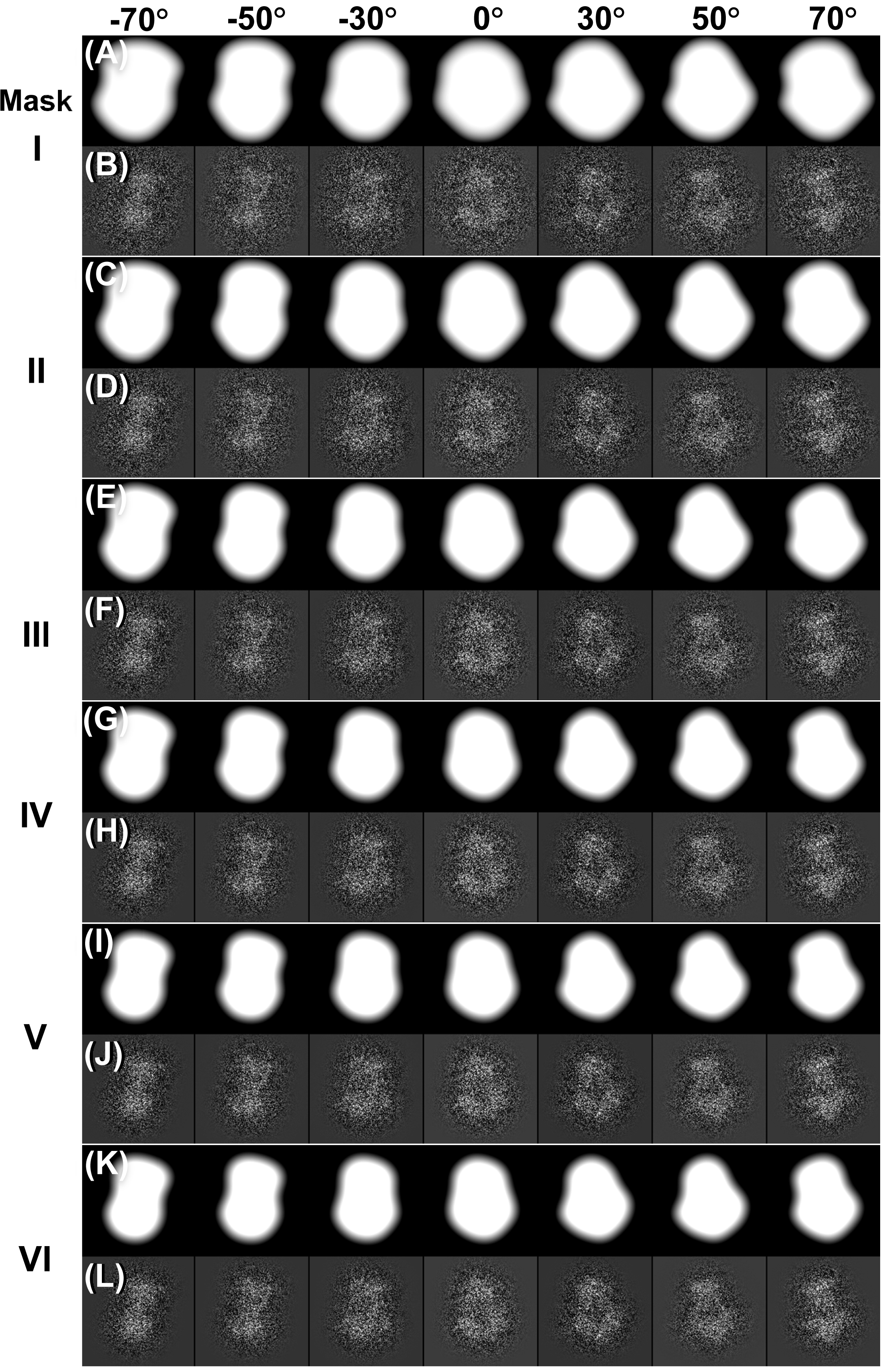

Supplement: Figure S6 — Monitoring the particle-shaped masks in FETR. A total of six automatically generated particle-shaped masks (A–L) were used to further reduce the noise and unnecessary background in the second round of iterations. To confirm that the signal of the targeted particle has not been eliminated or truncated, the masks had to be monitored during the iterations. The selected masked particles showed that no obvious portions of the particle were truncated by using these masks. (TIF) [file pone.0030249.s006.tif]

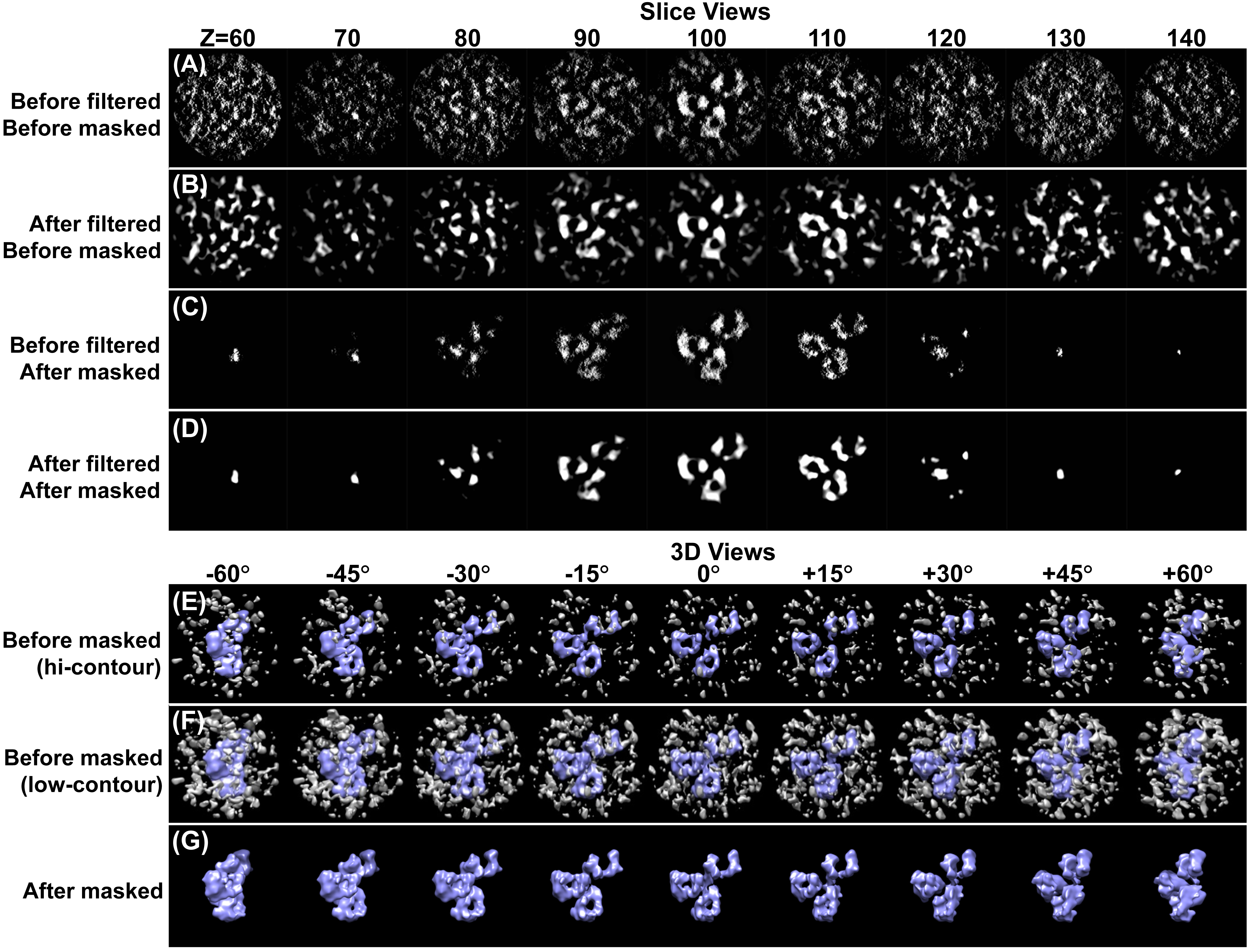

Supplement: Figure S7 — 3D reconstruction of first single-instance of IgG antibody by IPET/FETR. The antibody sample was prepared by an optimized NS protocol [45], [46] and imaged by ET. Selected slice views of 3D reconstruction before applying particle-shaped masks (A) before applying low-pass filters and (B) after applying low-pass filters; after the particle-shaped masks were applied, the slice views were shown (C) before applying low-pass filters and (D) after applying low-pass filter. (E) Selected tilted view of the 3D density map displayed at a high-contour level, (F) a low-contour level before applying the mask, and (G) the 3D density map after applying the mask. (TIF) [file pone.0030249.s007.tif]

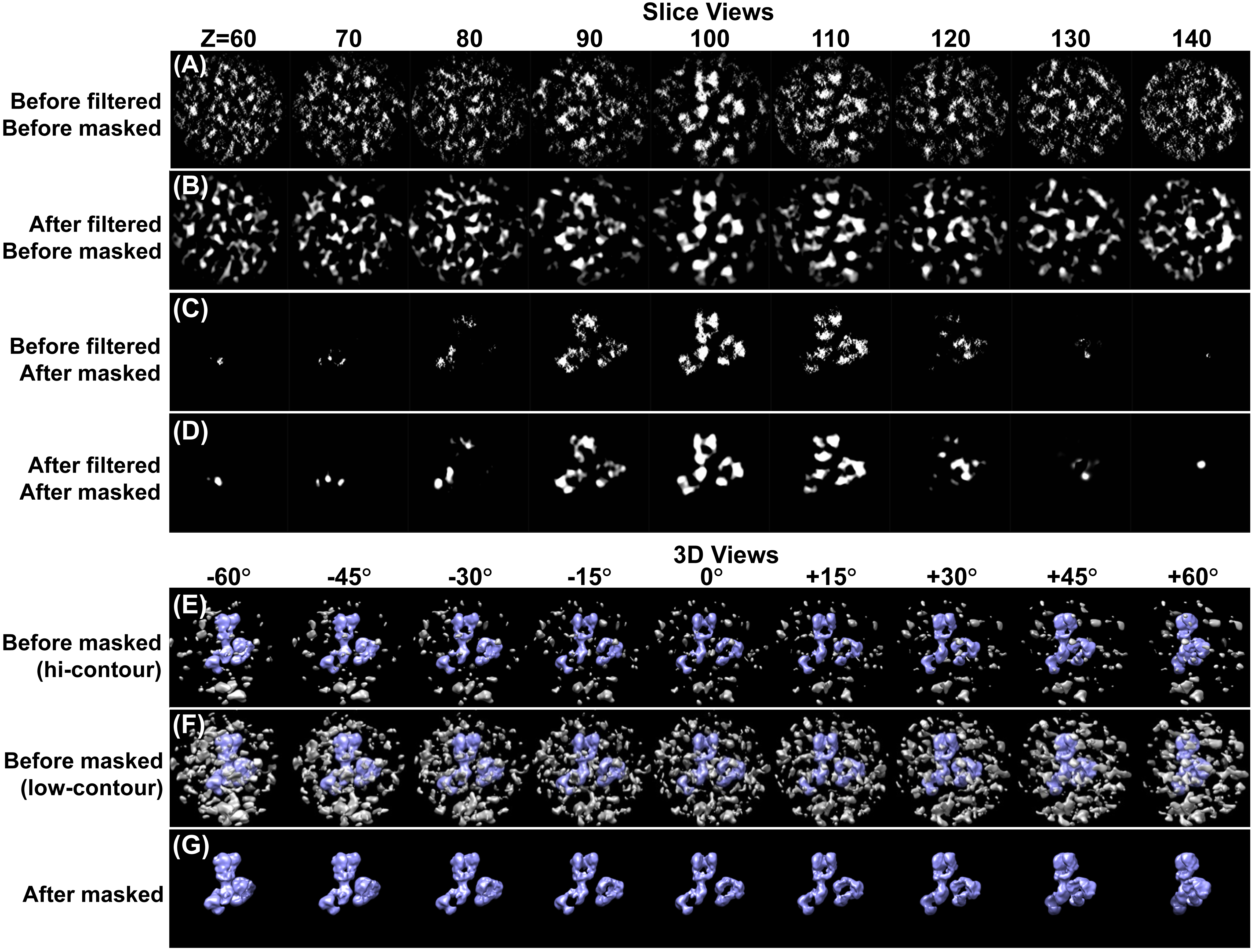

Supplement: Figure S8 — 3D reconstruction of second single-instance of IgG antibody by IPET/FETR. The antibody sample was prepared by an optimized NS protocol [45], [46] and imaged by ET. Selected slice views of 3D reconstruction before applying particle-shaped masks (A) before applying low-pass filters and (B) after applying low-pass filters; after the particle-shaped masks were applied, the slice views were shown (C) before applying low-pass filters and (D) after applying low-pass filter. (E) Selected tilted view of the 3D density map displayed at a high-contour level, (F) a low-contour level before applying the mask, and (G) the 3D density map after applying the mask. (TIF) [file pone.0030249.s008.tif]

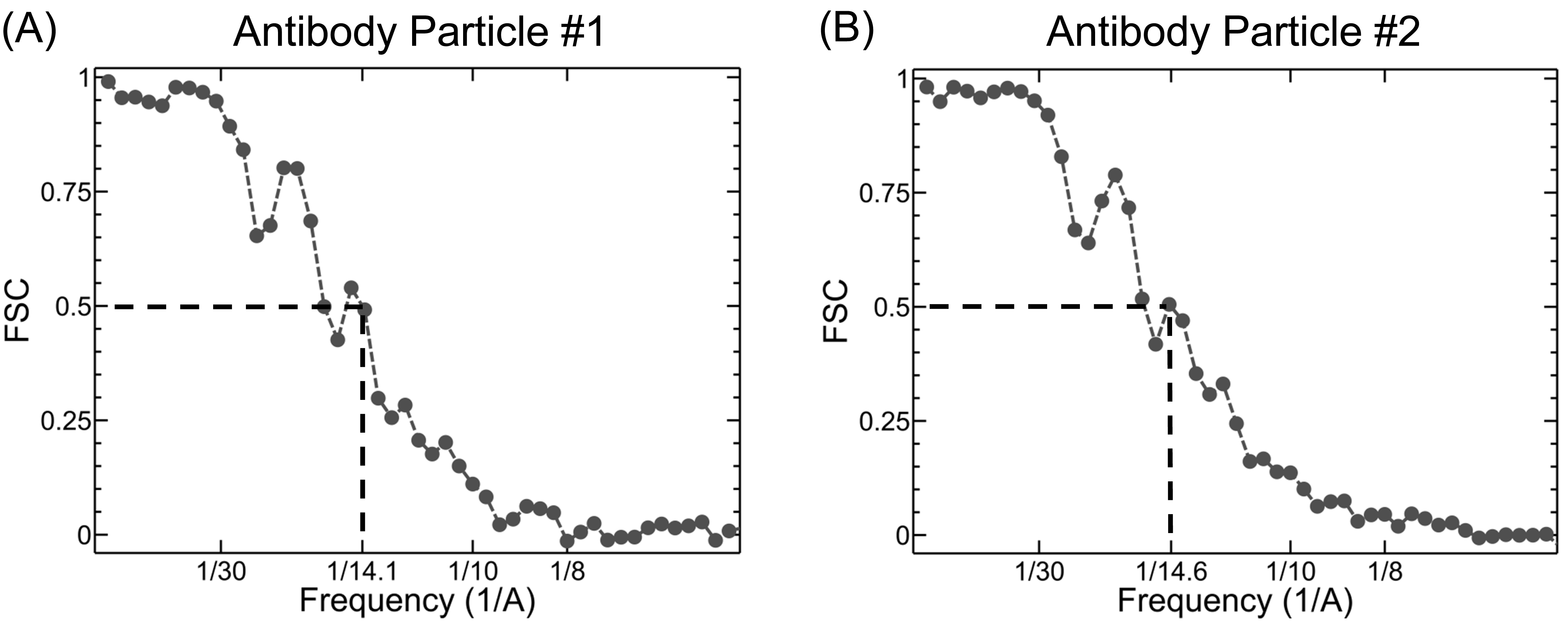

Supplement: Figure S9 — The intra-FSC analyses of two IgG antibody density maps reconstructed by IPET/FETR. By intra-f0.5 criterion, the intra-FSC showed that the resolution achieved by FETR are (A) ∼14.1 Å for antibody #1 and (B) ∼14.6 Å for antibody #2 respectively. (TIF) [file pone.0030249.s009.tif]

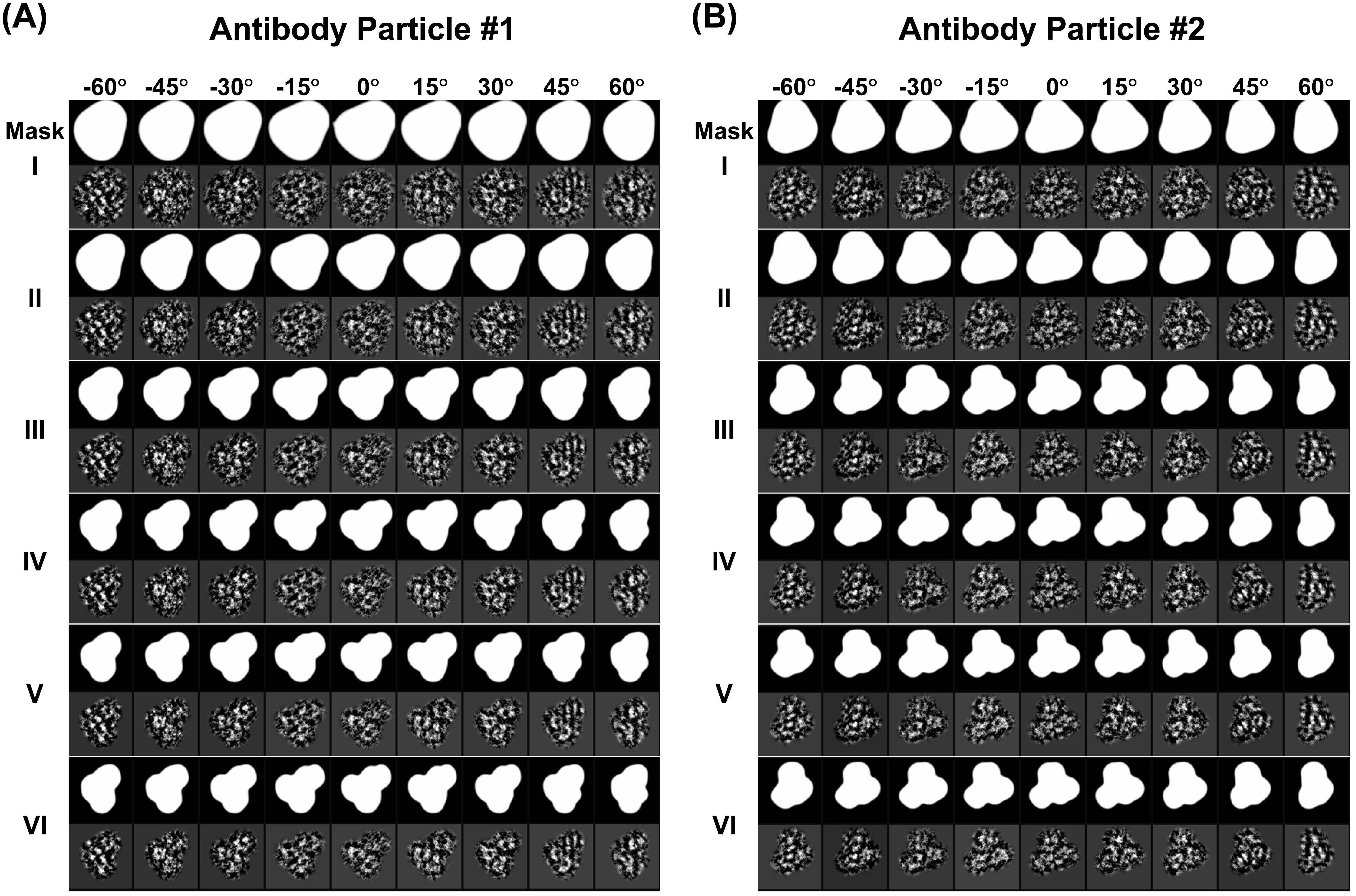

Supplement: Figure S10 — Monitoring the particle-shaped masks in two IgG antibodies reconstruction by IPET/FETR. During the second round of iterations, a total of six automatically generated particle-shaped masks were used to further reduce the noise and unnecessary background while generating the 3D reconstructions of two IgG antibodies. To confirm that the signal of the targeted particle was not been eliminated or truncated, the masks were monitored during the iterations. The selected masked particles showed that no obvious portions of the particle were truncated by using these masks for antibody number one (A) and two (B). (TIF) [file pone.0030249.s010.tif]

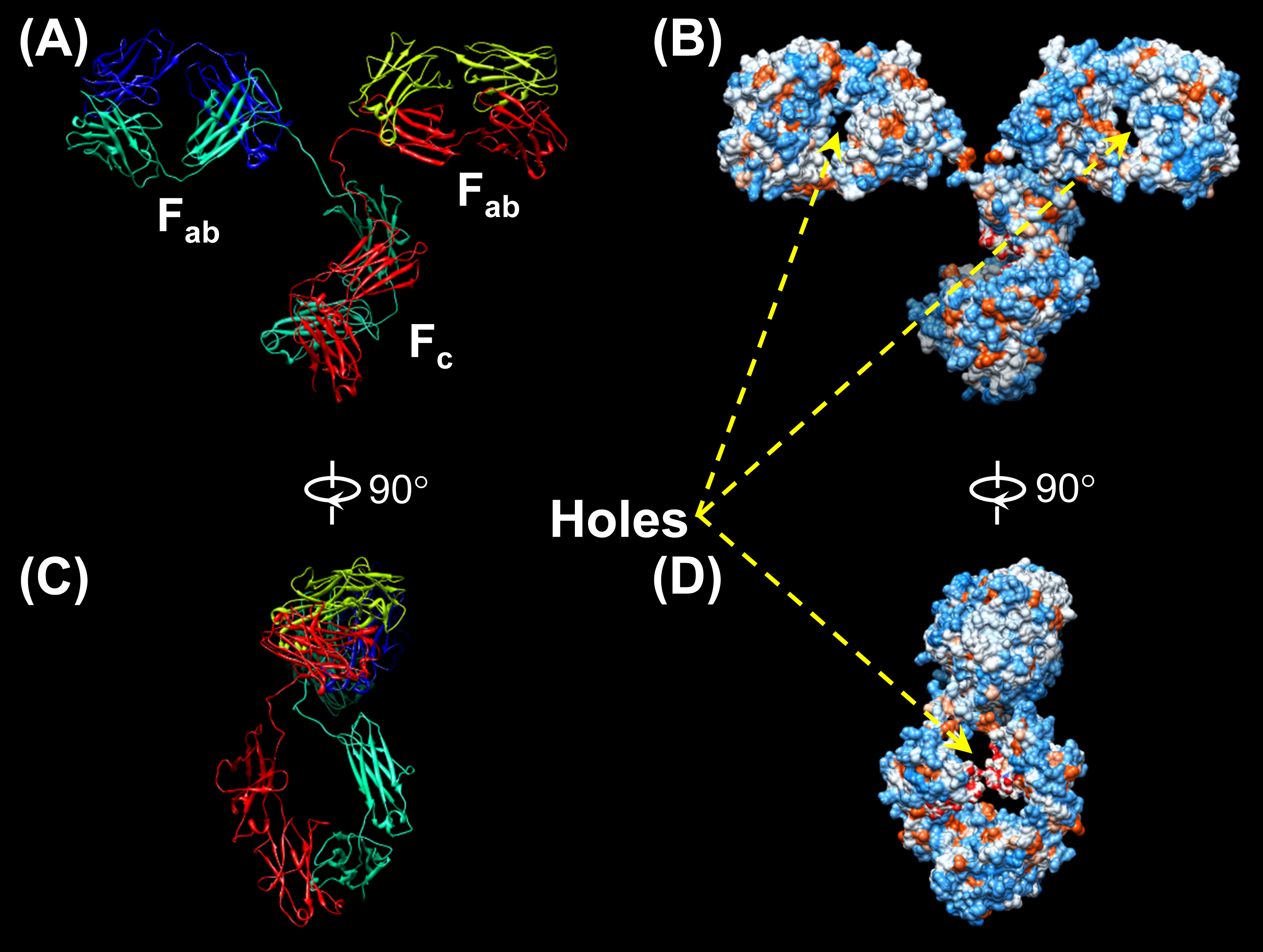

Supplement: Figure S11 — The crystal structure of IgG antibody (PDB entry 1IGT) displays a hole within each domain. (A) By displaying the crystal structure in ribbon, and (B) van der Waals surface, the holes were displayed clearly within the Fab domains. By 90° rotation along the Y-axis, both (C) the ribbon and (D) van der Waals surface images display an even bigger hole within the Fc domain, suggesting the hole in each domain is the intrinsic structure feature in the IgG antibody. (TIF) [file pone.0030249.s011.tif]

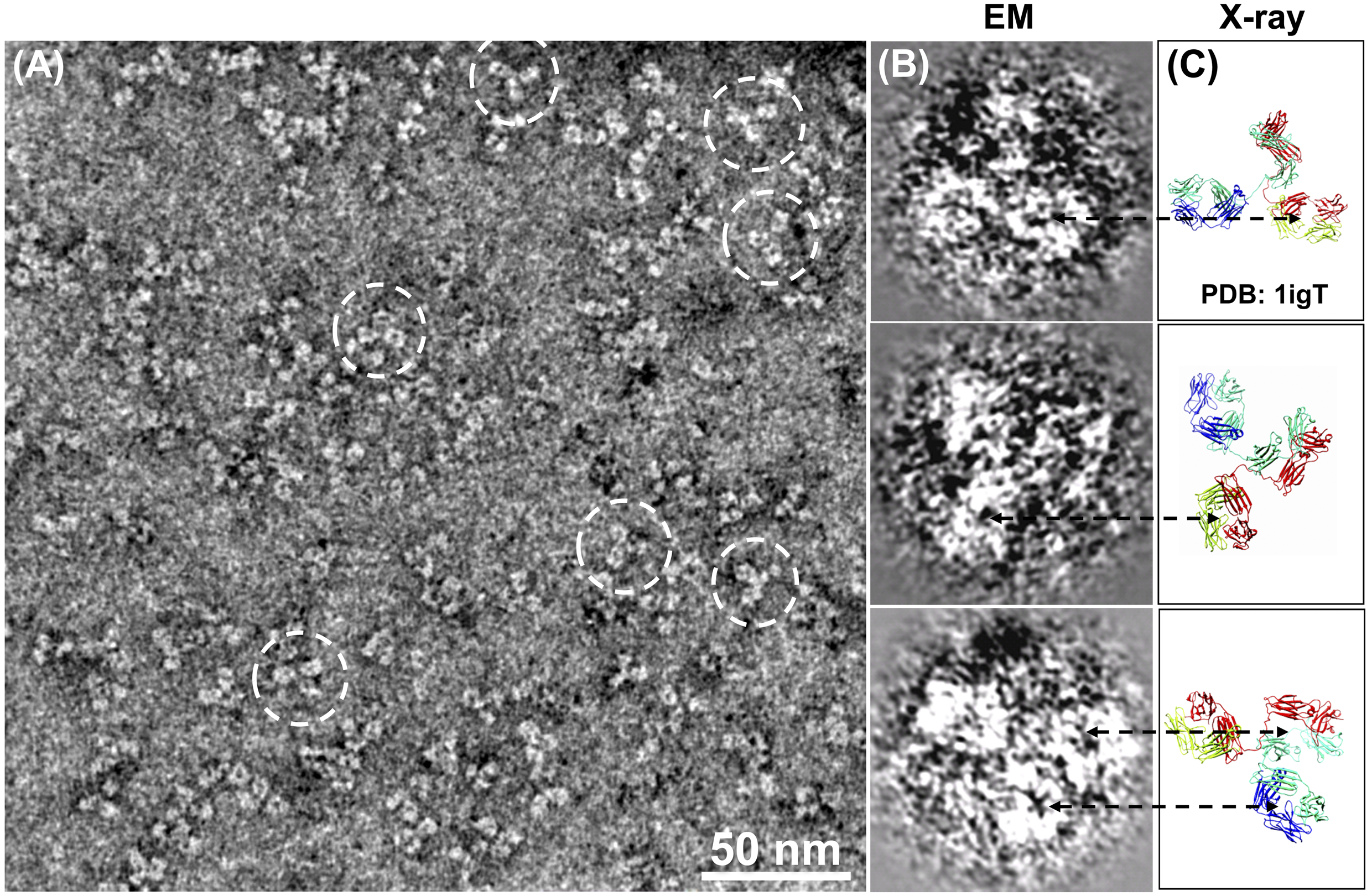

Supplement: Figure S12 — Human IgG antibody particles prepared by optimized negative-staining protocol and imaged at near Scherzer focus. (A) Survey view of human IgG antibody imaged. The white-circled particles clearly displayed three domains within each particle. (B) Selected three particles display low-density regions (holes indicated by dash arrows) within domains. (C) Their corresponding orientations of the crystal structure (PDB entry 1IGT) displayed in their corresponding holes within the corresponding domains can also be visualized, suggesting the holes are the intrinsic structure features instead of the artifact from neither negative-staining nor defocus-related contrast transfer function (CTF). (TIF) [file pone.0030249.s012.tif]

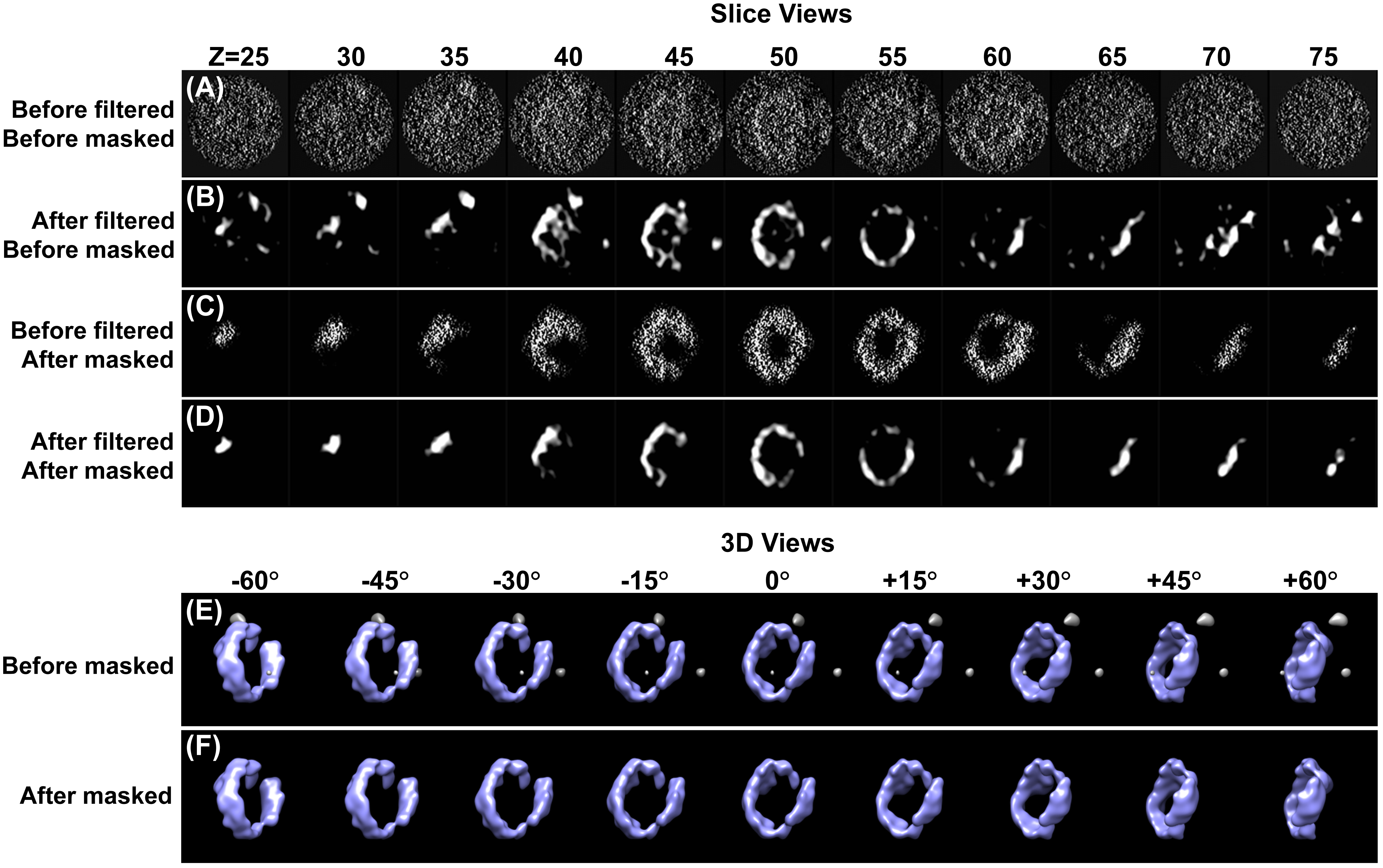

Supplement: Figure S13 — 3D reconstruction of first single-instance of nascent HDL particle from the cryoET images by IPET method. (A) Selected slice views of 3D reconstruction before applying low-pass filtering and particle-shaped masks; and (B) their corresponding slice views of 3D reconstruction after low-pass filtering, but before masking; and (C) the corresponding slice views before filtering, but after masking; and (D) views after both filtering and masking. (E) Selected tilted view of the 3D density map before applying the mask, and (F) the corresponding views of 3D density map after applying the mask, showing only few isolated small densities (in gray in E) were truncated by the mask. (TIF) [file pone.0030249.s013.tif]

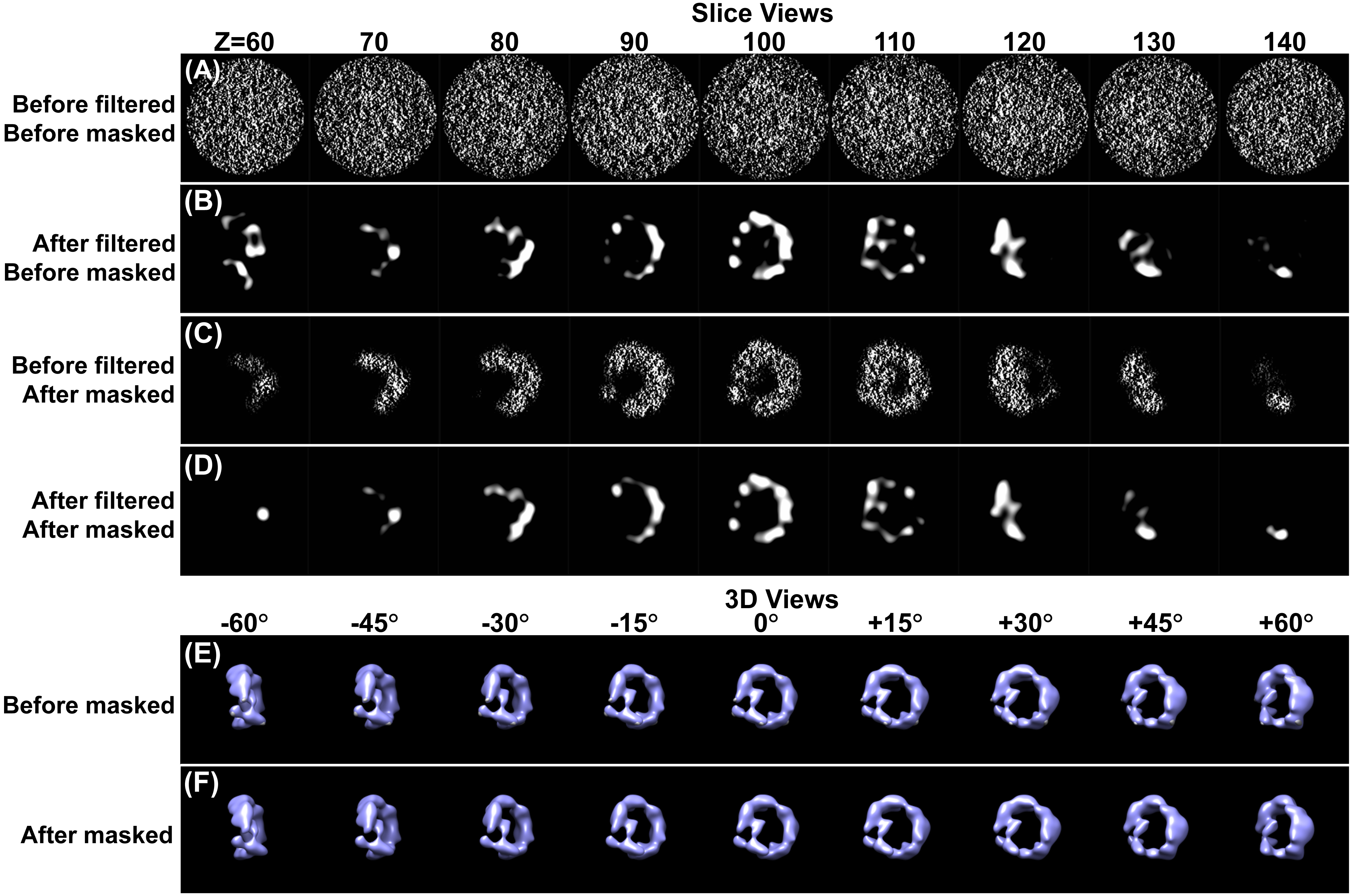

Supplement: Figure S14 — 3D reconstruction of second single-instance of nascent HDL particle from the cryoET images by IPET method. (A) Selected slice views of 3D reconstruction before applying low-pass filtering and particle-shaped masks; and (B) their corresponding slice views of 3D reconstruction after low-pass filtering, but before masking; and (C) the corresponding slice views before filtering, but after masking; and (D) views after both filtering and masking. (E) Selected tilted view of the 3D density map before applying the mask, and (F) the corresponding views of 3D density map after applying the mask. (TIF) [file pone.0030249.s014.tif]

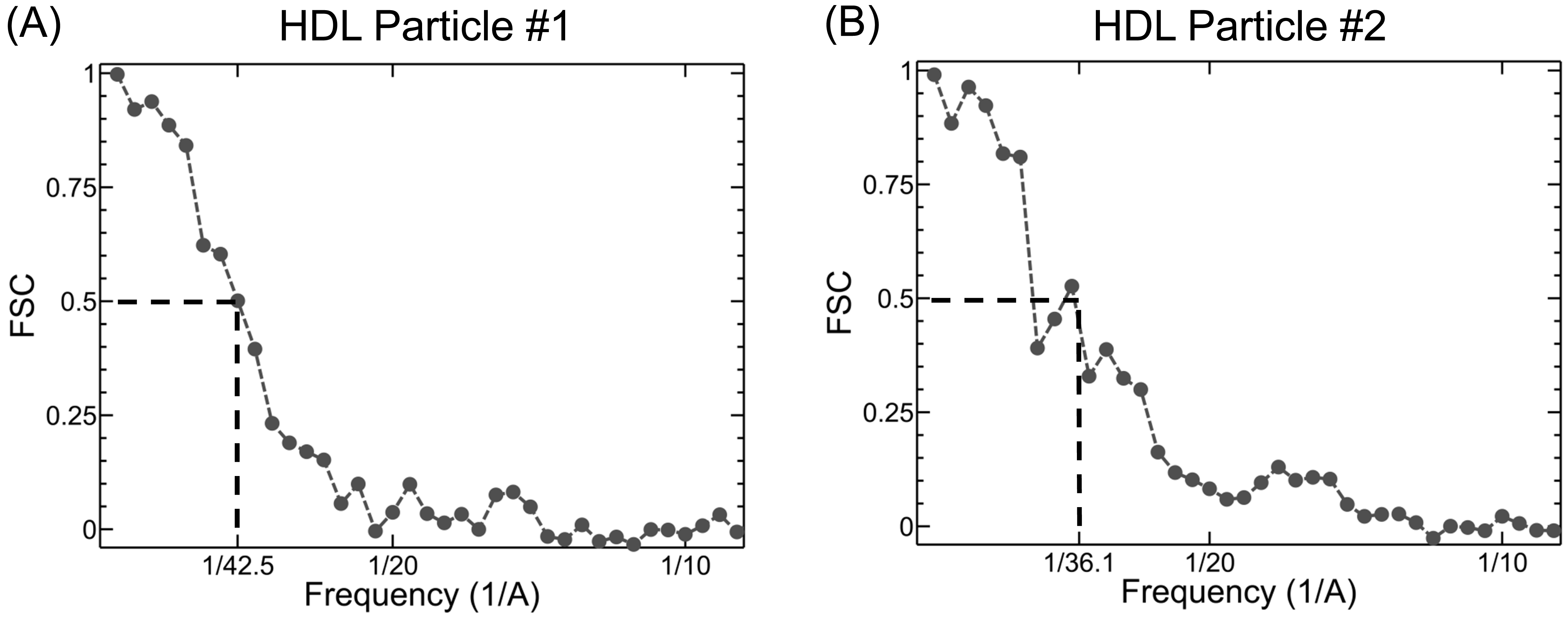

Supplement: Figure S15 — The intra-FSC analyses of two nascent HDL density maps reconstructed by IPET method. By the intra-f0.5 criterion, intra-FSC curves showed that the resolution achieved by FETR algorithm are (A) ∼42.5 Å for nascent HDL particle #1 and (B) ∼36.1 Å for HDL particle #2. (TIF) [file pone.0030249.s015.tif]

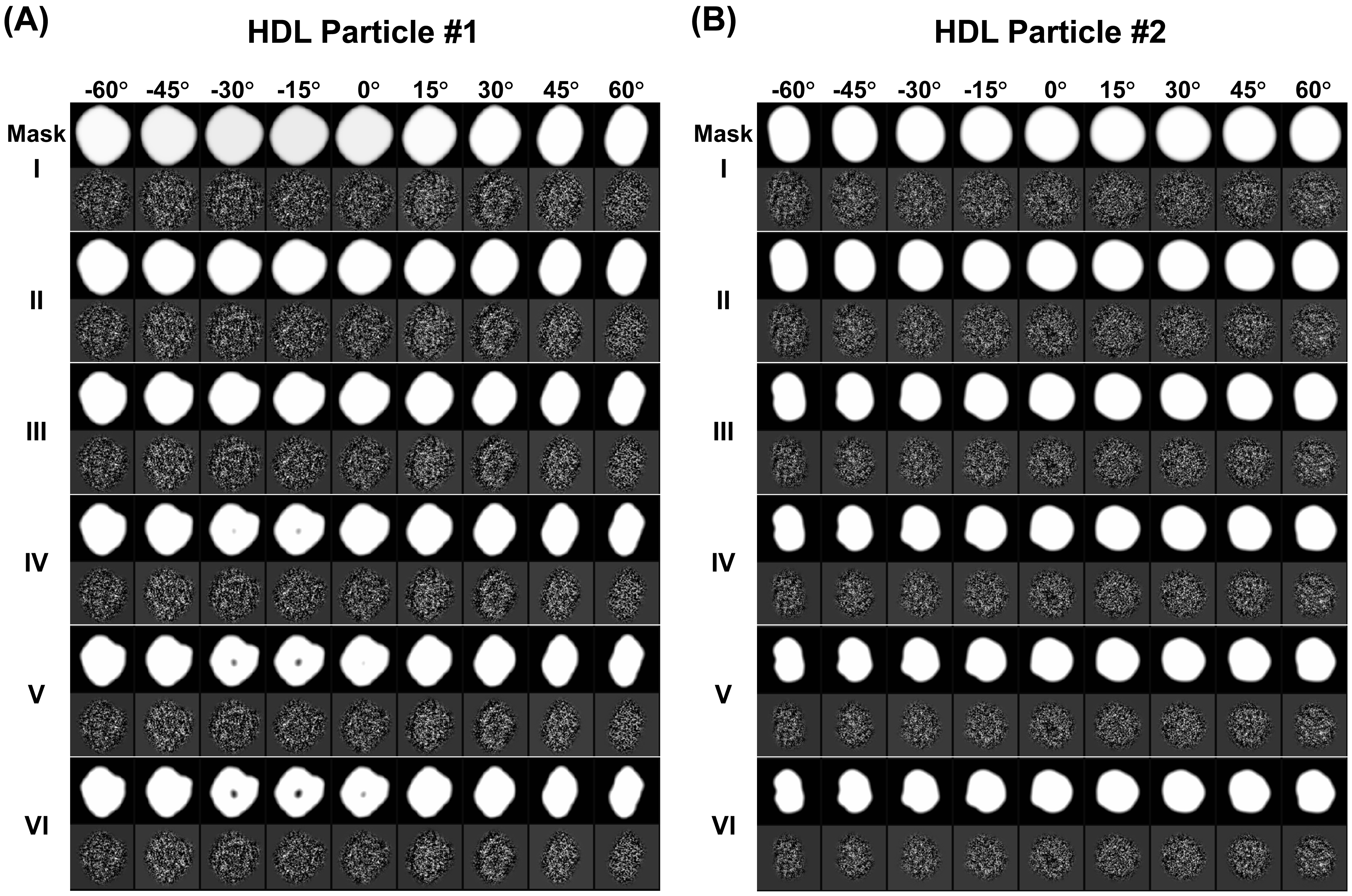

Supplement: Figure S16 — Monitoring the particle-shaped masks in the nascent HDL reconstruction by IPET method. To confirm that the signal of the targeted nascent HDL particle has not been eliminated or truncated during the second round of iterations, where a set particle-shaped masks were applied on the raw particle images to eliminate the noise contribution to the translational searching. The masks were monitored during the iterations. Six masks and masked particles were displayed, suggesting that no obvious portions of the particle were truncated in the IPET method for 3D reconstruction of nascent HDL number one (A) and two (B). (TIF) [file pone.0030249.s016.tif]

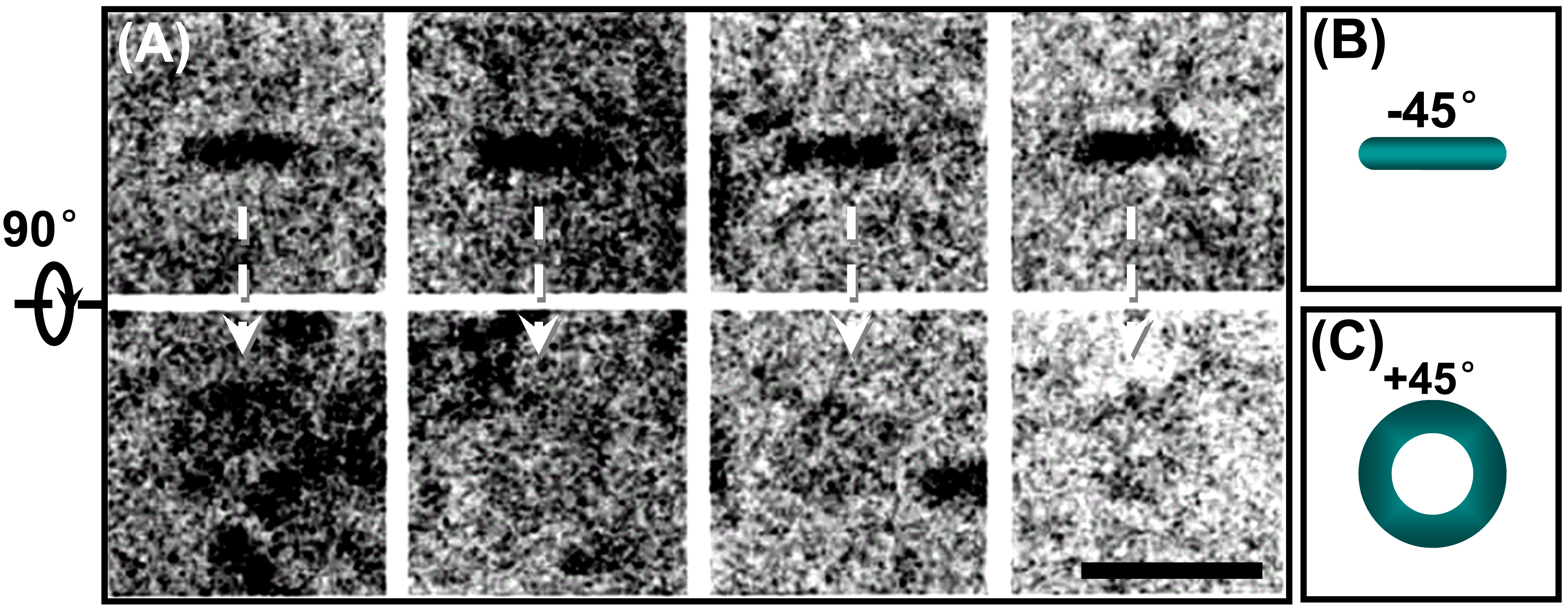

Supplement: Figure S17 — Discoidal shape of 17 nm nascent HDL particle reported by the conventional cryoEM imaged from two orthogonal tilt views. (A) van Antwerpen et al. investigated the 17 nm HDL particle shape by cryoEM. HDL particles were embedded in vitreous ice and imaged from two orthogonal tilt-viewing angles. Four selected particles (top panel) are represented with rod-shape (B), while their corresponding 90° tilted views present a circular shape (C). Thus, van Antwerpen et al. proposed a discoidal shape model for 17 nm HDL particles. (This research was originally published in Journal of Lipid Research. van Antwerpen R, Chen GC, Pullinger CR, Kane JP, LaBelle M, et al. Cryo-electron microscopy of low density lipoprotein and reconstituted discoidal high density lipoprotein: imaging of the apolipoprotein moiety. J. Lipid Res. 1997; 38: 659–669. © the American Society for Biochemistry and Molecular Biology). (TIF) [file pone.0030249.s017.tif]

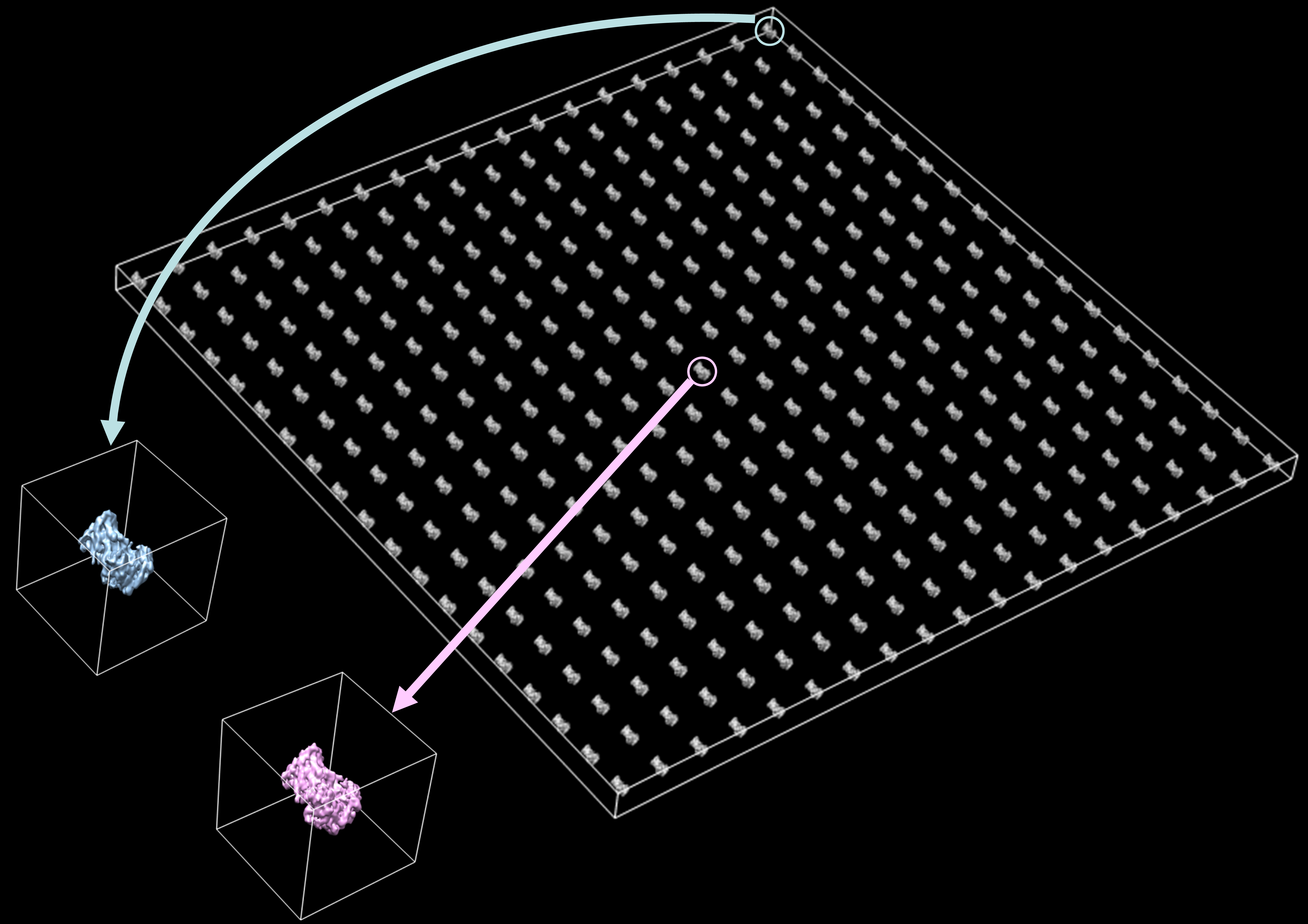

Supplement: Figure S18 — 3D reconstruction from a whole-micrograph-size tilt images that contain the tilt-errors. To demonstrate the effect of tilt-errors on the 3D reconstruction, we back-projected a set of 141 simulated micrograph-size noise-free images (4120×4120 pixels) containing tilt-axis and/or tilt-angle errors in a defined range such as ±0.5°. The particle-density maps (subvolumes) were windowed from different spatial location from the large micrograph reconstruction (4120×4120×160 voxels). (TIF) [file pone.0030249.s018.tif]

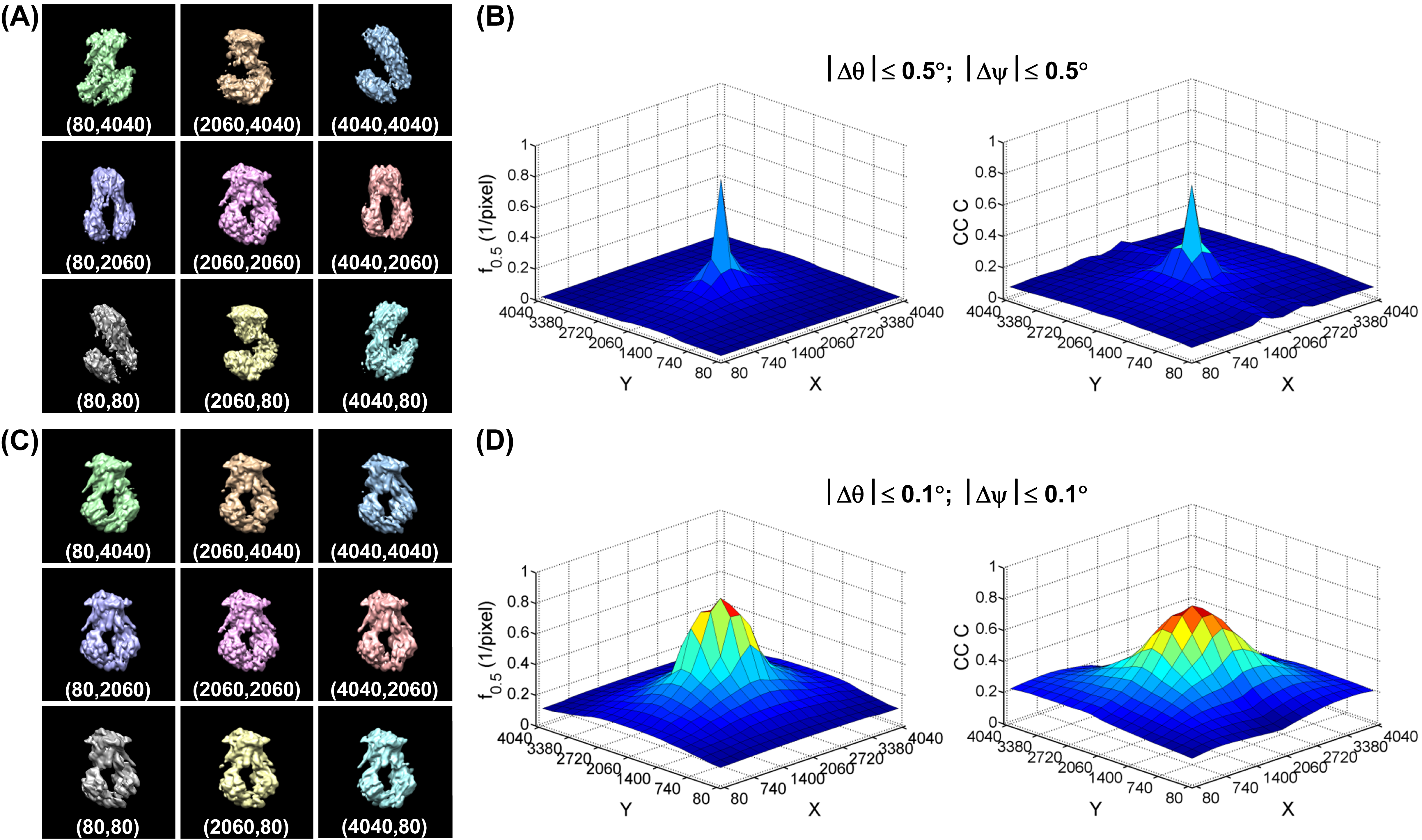

Supplement: Figure S19 — Effect of tilt-error (including both tilt-axis and tilt-angle errors) in whole-micrograph-size reconstruction. (A) To demonstrate the effects of tilt-error in the 3D reconstruction, both tilt-axis and tilt-angle were introduced with a random error within a range of ±0.5°. The particle density maps (subvolumes) were windowed from different spatial locations from the large-micrograph reconstruction (4120×4120×160 voxels, Figure S18). The quality of the 3D reconstructions of the objects were dependent on the positions of the objects. The selected particles/subvolumes showed that the reconstruction from the center contains more similarity to that from the edge. (B) To quantitatively evaluate the quality of each reconstructed particle against its spatial location, a Fourier shell correlation (FSC) curve and cross-correlation coefficient (CC C) value between each reconstructed particle and object was computed as shown. By plotting the f0.5 (left) and CC C (right) values of the particles against their in-plane locations, the topography of the f0.5 and CC C showed a peak near the center of the specimen, suggesting the highest quality of reconstruction was at the center. (C) To demonstrate the effect of ±0.1° tilt-errors on the 3D reconstruction, the particle-density maps were windowed from different spatial location from the large micrograph reconstruction. The selected particles/subvolumes showed that the reconstruction from the center contains the highest quality. (D) By plotting the f0.5 (left) and CC C (right) values of the particles against their in-plane locations, the topography of the f0.5 and CC C showed a peak near the center of the specimen, suggesting the highest quality of reconstruction was at the center. Both distributions showed a sharp peak at the center area, suggesting that only the particles near the center of reconstruction area had the highest degree of similarity to the object, and further suggesting the center subvolume can tolerate high degree of ti [file pone.0030249.s019.tif]

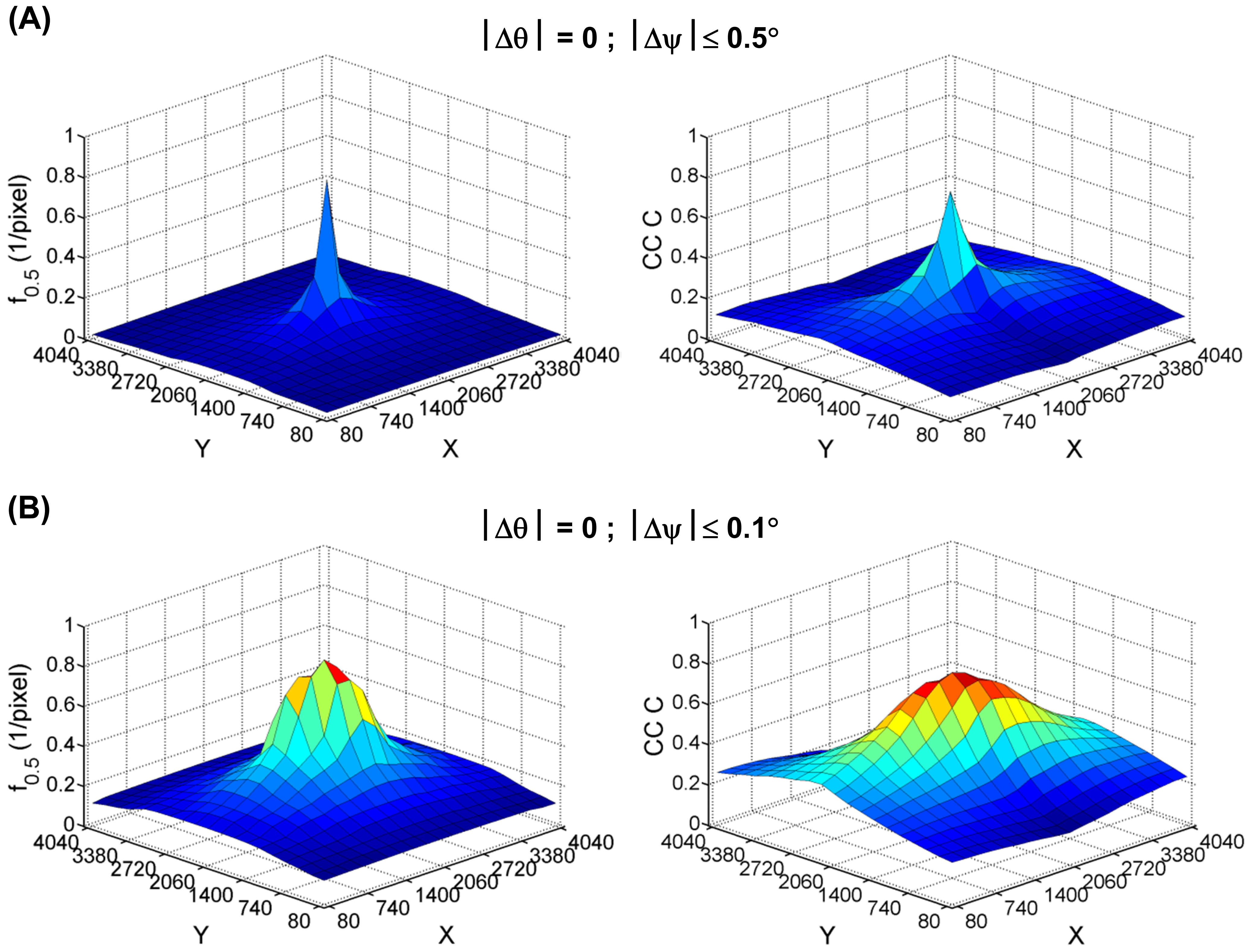

Supplement: Figure S20 — Effect of random tilt-axis error in the whole-micrograph-size reconstruction. To better understand the effect from random error of tilt-axis, we repeated above test by tilt-axis only containing a random errors in a range of (A) ±0.5° and (B) ±0.1°. The whole-micrograph reconstruction was analyzed by comparing each subvolume to the object to compute the FSC curves and CC values. The topographies of the f0.5 (left) and CC value (right) were displayed against their position in micrograph. Both tests showed that the particles/subvolumes near the micrograph central area still retained their best similarities to the object, while the particles/subvolumes near the corners consistently retained their least similarities to the object based on f0.5 and CC analyses. The distribution had a much narrow peak, but with similar height, in the larger tilt-axis errors (±0.5°) than the smaller errors (±0.1°), suggesting that the reconstruction near image center can tolerate a higher level of tilt-axis measurement error. (TIF) [file pone.0030249.s020.tif]

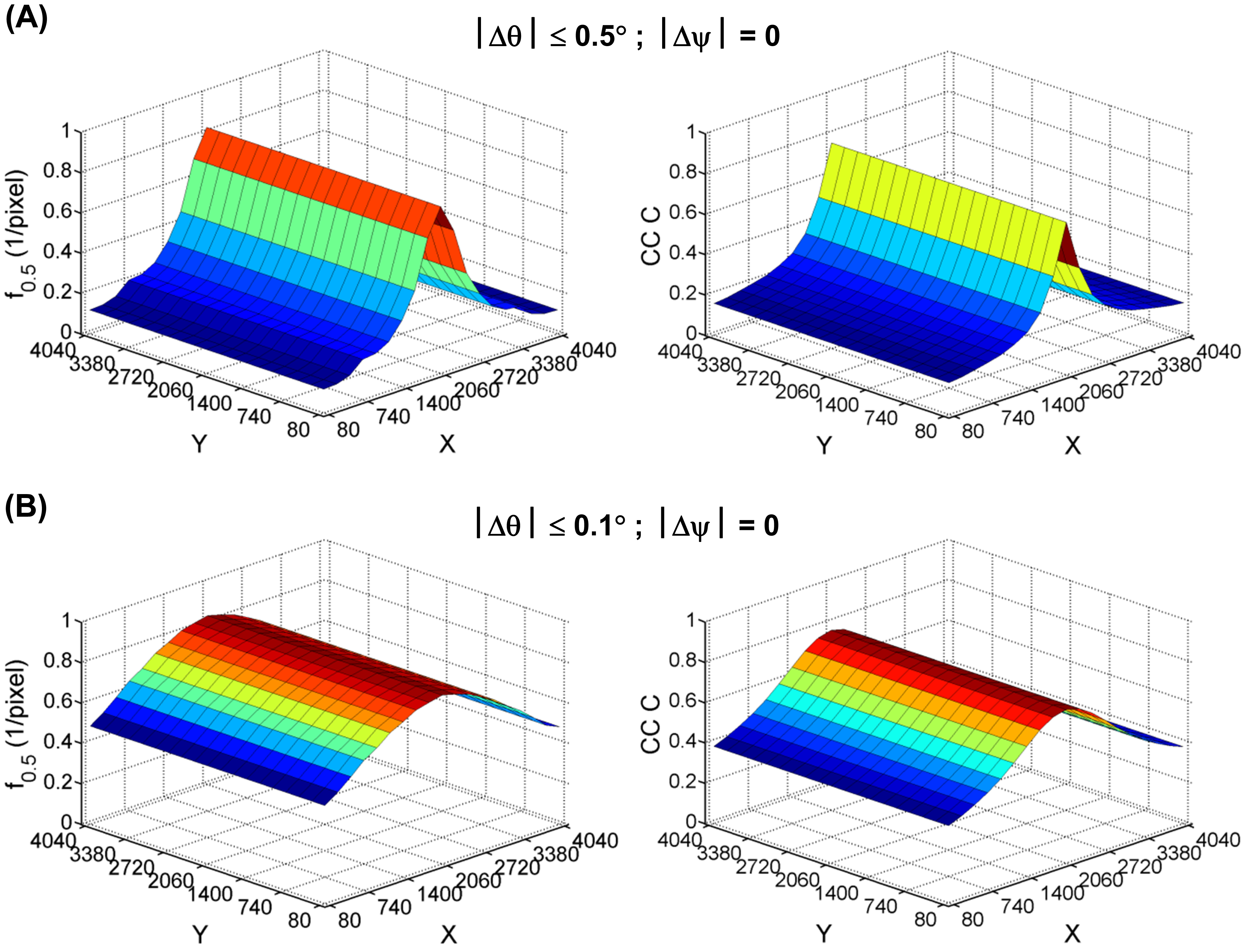

Supplement: Figure S21 — Effect of tilt-angle error in the whole-micrograph-size reconstruction. Containing only the tilt-angle random errors in a range of (A) ±0.5° and (B) ±0.1°, the whole-micrograph reconstruction was analyzed by comparing each subvolume to the object for computing the FSC curves and CC values. The topographies of the f0.5 (left) and CC value (right) were displayed against their position in micrograph. Both analyses showed that the particles/subvolumes near the tilt-axis area have the best similarity to the object, while the particles/subvolumes far from tilt-axis area have the least similarity to the object. The distribution had a much narrow ridge, but with similar height in the larger tilt-angle errors (±0.5°) than the smaller error (±0.1°), suggesting that the reconstruction near tilt-axis can tolerate a higher level of tilt-angle measurement error. (TIF) [file pone.0030249.s021.tif]

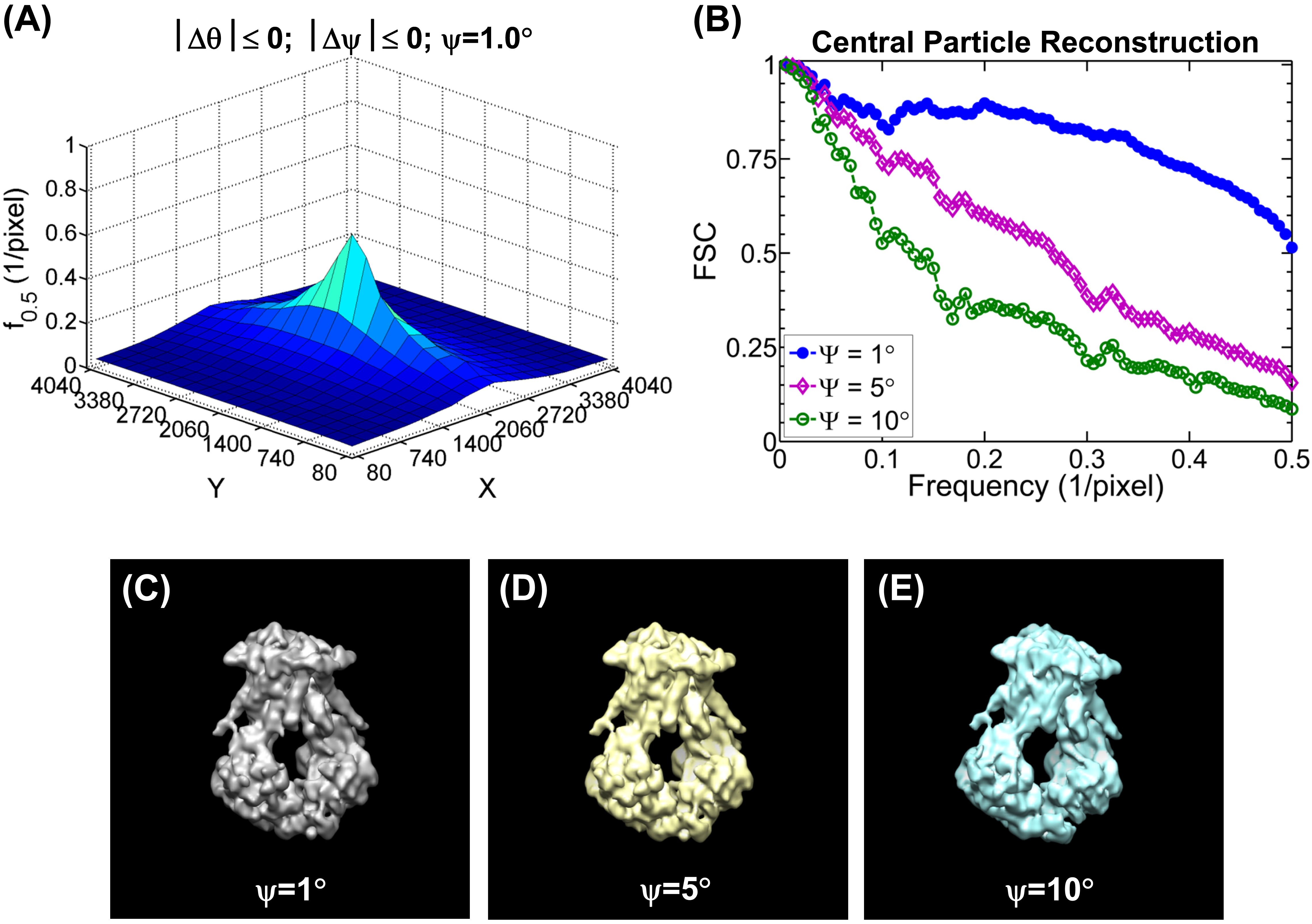

Supplement: Figure S22 — Effect of systematic tilt-axis error in the whole-micrograph-size reconstruction. A similar test was repeated by only introducing a fixed systemic tilt-axis error of 1.0° (no any other error). (A) The f0.5 distribution showed a center peak, suggesting the central subvolume retained its highest similarity to the model. The subvolumes near the tilt-axis are generally better than that far from tilt-axis. (B) By increasing the systemic tilt-axis error from 1.0° to 5.0° and 10.0°, the FSC curves calculated between the object and each central subvolume displayed the center subvolume retains its similarity to the object up to resolution of 10 Å. (C–E) After it was low-pass filtered for each central subvolume up to 8 Å, the three subvolumes displayed near identical similarity, except tilting, suggesting the central subvolume can tolerate a relative large tilt-axis symmetric error. (TIF) [file pone.0030249.s022.tif]

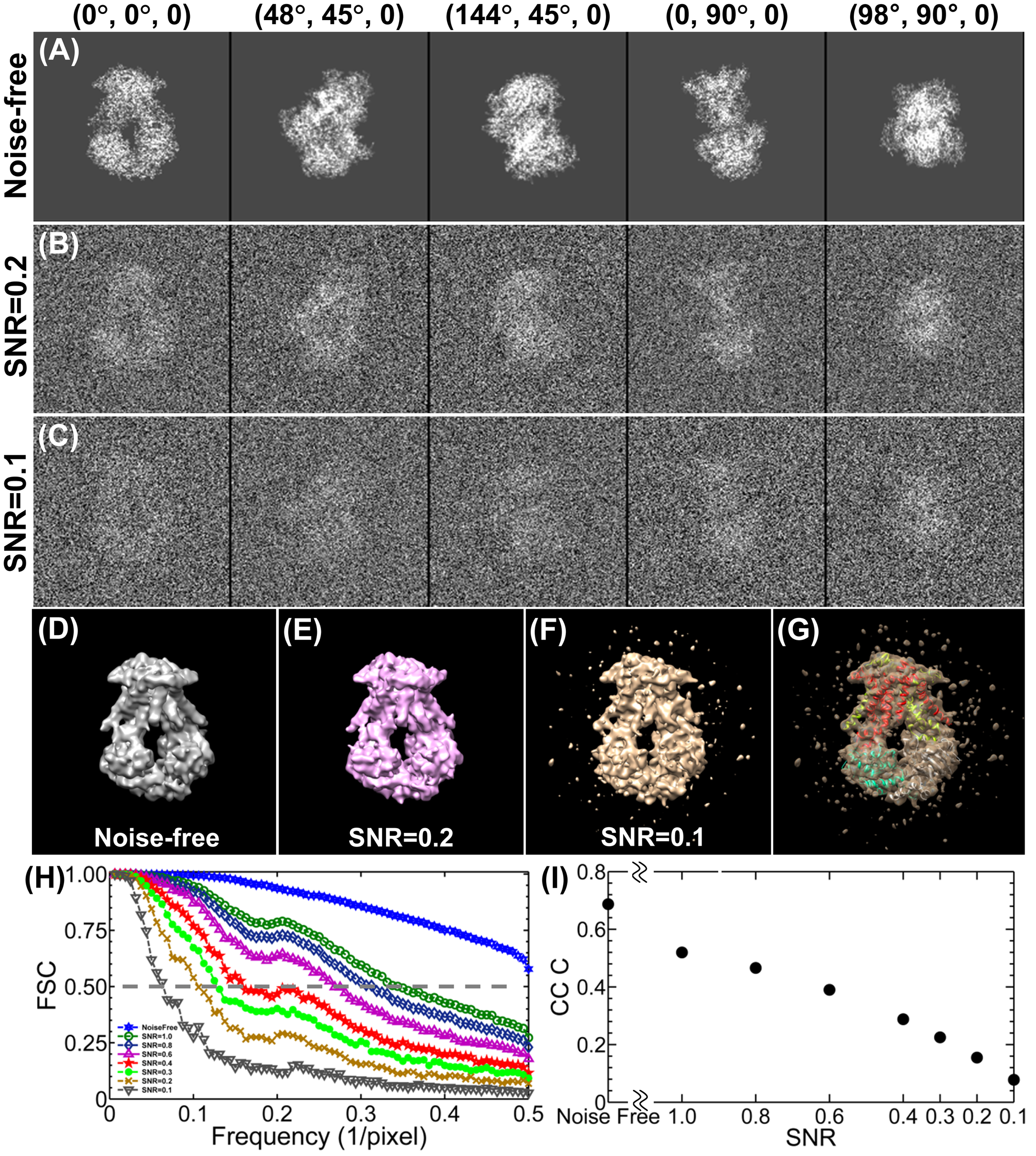

Supplement: Figure S23 — Noise effects in the single-particle 3D reconstructions. (A) 84 projections were generated by projecting the object based on a set of single-particle Euler angles, i.e. a sampling angle of 15°. Five represented projections were displayed. By adding eight different levels of noise to the projections, such as (B) SNR = 0.2 and (C) SNR = 0.1 that are similar to the noise level presented in cryoEM images, we back-projected each set of noisy images by following same Euler angles as for projection. The 3D reconstructions were then low-pass filtered to 8 Å. Three 3D reconstructions, (D) noise-free, (E) SNR = 0.2, and (F) SNR = 0.1 were displayed, in which, (G) the 3D reconstruction of SNR = 0.1 was docked with the model of crystal structure. The reconstructions showed high similarity to each other in the term of the detailed structure, such as α-helices. (H) Using the quantitative analyses of the 3D reconstructions, the FSC curves were computed by comparing each 3D reconstruction to the object. The FSC curves showed that all f0.5 values were close to or beyond 1/10 Å−1. (I) By the real space analyses, the cross-correlation coefficients (CC C) between the object and each reconstruction showed a similar trend of f0.5 values. (TIF) [file pone.0030249.s023.tif]

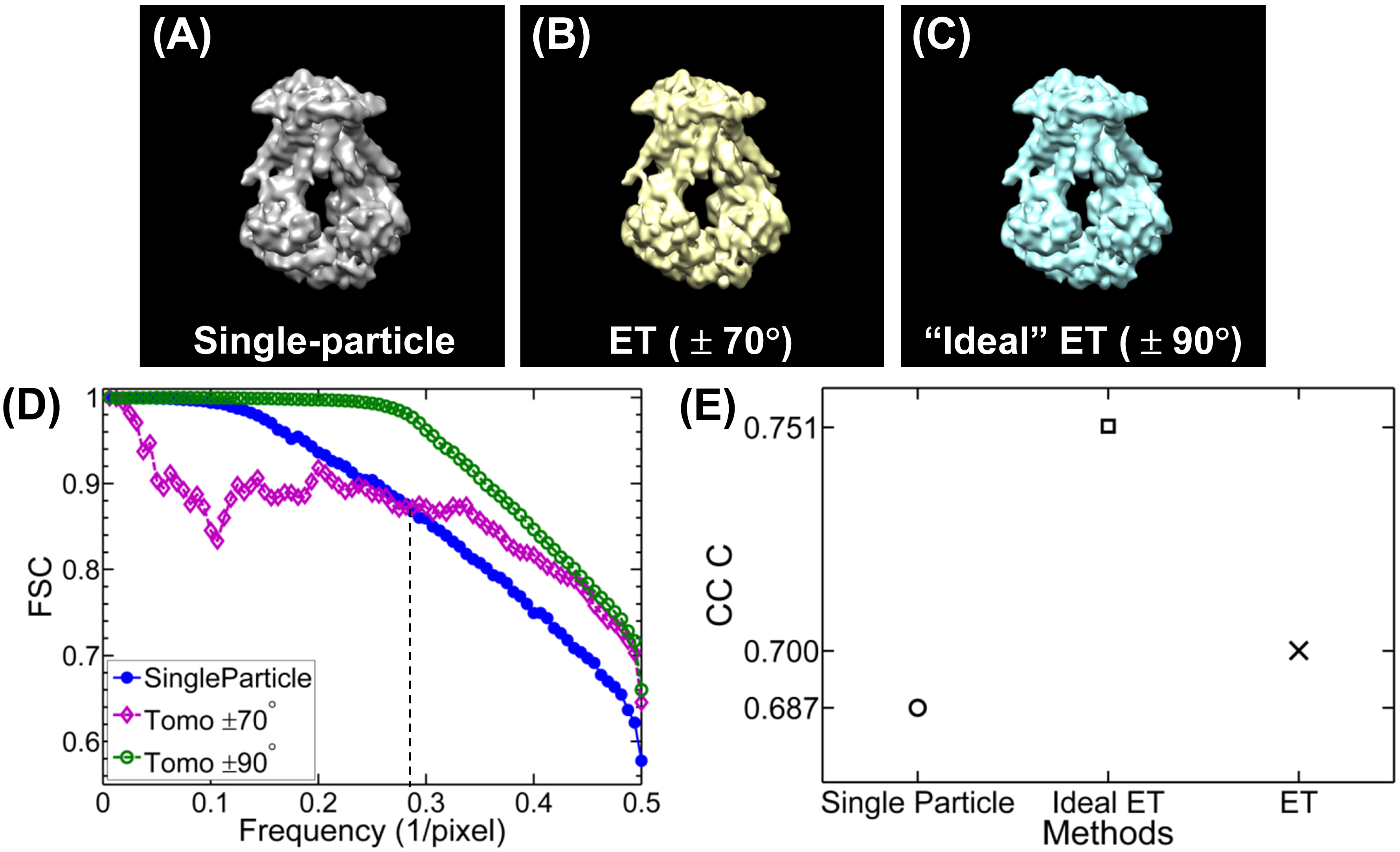

Supplement: Figure S24 — Missing-wedge effect in the 3D reconstruction of a thin and small protein. Three density maps, named single-particle map, ET map, and “ideal” ET map, were back-projected from three sets of 84 images that were projected from the same object, but using different sets of projection angles. (A) The single-particle map was reconstructed from single-particle projections based on a set of single-particle Euler angles (sampling angle of 15°). (B) The ET map was reconstructed from the ET projections and based on a set of ET Euler angles, tilt-angles were evenly distributed in the range from −70° to +70°. (C) The “ideal” ET map was same as the ET map, except the tilt-angles were evenly distributed in a range from −90° to +90°. After they were low-pass filtered to 8 Å, these three maps displayed no obvious differences. (D) To quantitatively analyze the quality of each map, FSC curves between the object and each map were calculated and plotted. All three curves were above 0.5 at the nyquist frequency (0.5 Å−1). In the relatively low-resolution zone (<∼0.28 Å−1), FSC curves showed the ET map had lower or reduced similarity to the object than single-particle map. However, in the relatively high-resolution zone (>∼0.28 Å−1), FSC analyses showed the ET map had increased similarity to the object than single-particle map. Overall, the “ideal” ET map remains as the best quality at any frequency. (E) Quantitative analyses of the quality of three maps were also performed in real space. By calculating the cross-correlation coefficient (CC C) between each map to the object, the “ideal” ET map retains its most similarity to the object, while the single-particle and ET maps have about equal similarity to the object, but EM map is slightly better than the single-particle map. (TIF) [file pone.0030249.s024.tif]
