# Supplementary material for: IPET and FETR: Experimental Approach for Studying Molecular Structure Dynamics by Cryo-Electron Tomography of a Single-Molecule Structure
Source: PLoS One. 2012 Jan 24;7(1):e30249. doi: 10.1371/journal.pone.0030249 (PMC3265479; doi:10.1371/journal.pone.0030249)
Supplement: Table S1 — Defocus-introduced image distortion. Tracking the movements of 70 particles (5 nm nanogold particles) that were imaged under the defocus changes from 0.0 µm to 10 µm in steps of 0.5 µm was used for quantitative determination of the defocus-introduced distortion under a non-parallel beam conditions. The coordinates of each particle imaged under the different defocus were fitted into a 2nd degree polynomial function, i.e. u = a0+a1x+a2y+a3x2+a4y2+a5xy, v = b0+b1x+b2y+b3x2+b4y2+b5xy. The fitting parameters were defined by a least squares fitting method using Matlab. (DOC) [file pone.0030249.s025.doc]

**Table S1. Defocus-introduced image distortion.**

| **DF (µm)** | ***a_0_*** | ***a_1_*** | ***a_2_*** | ***a_3_*** | ***a_4_*** | ***a_5_*** |
| --- | --- | --- | --- | --- | --- | --- |
| **0.0** | **5.12E-13** | **1** | **-2.20E-16** | **-1.25E-19** | **-1.93E-19** | **-1.35E-20** |
| **0.5** | **1.646337** | **0.996748** | **0.0004601** | **-1.68E-07** | **1.03E-07** | **-1.07E-07** |
| **1.0** | **2.656421** | **0.993289** | **0.0007029** | **-2.41E-07** | **-4.17E-08** | **-1.40E-07** |
| **1.5** | **3.043253** | **0.989827** | **0.0009369** | **-5.59E-08** | **9.59E-08** | **-1.66E-07** |
| **2.0** | **4.55616** | **0.986431** | **0.0013278** | **-1.90E-07** | **-4.72E-08** | **-1.67E-07** |
| **2.5** | **5.965206** | **0.982676** | **0.001565** | **-2.65E-07** | **-1.87E-07** | **-1.56E-07** |
| **3.0** | **7.052933** | **0.979042** | **0.0019554** | **-1.24E-07** | **-2.11E-07** | **-6.99E-08** |
| **3.5** | **7.4579** | **0.975522** | **0.0024233** | **2.98E-08** | **-1.72E-07** | **-2.16E-07** |
| **4.0** | **8.482748** | **0.97145** | **0.0027589** | **3.20E-08** | **-1.63E-07** | **-4.05E-07** |
| **4.5** | **10.05292** | **0.968299** | **0.0029616** | **-4.82E-07** | **-2.08E-07** | **-1.01E-07** |
| **5.0** | **11.44288** | **0.964665** | **0.0031491** | **-3.55E-07** | **-1.07E-07** | **-1.18E-07** |
| **5.5** | **12.82995** | **0.960932** | **0.0033634** | **-3.80E-07** | **-1.11E-07** | **-1.05E-07** |
| **6.0** | **13.52231** | **0.956743** | **0.0035872** | **-1.87E-07** | **-3.64E-08** | **-1.75E-07** |
| **6.5** | **15.82926** | **0.953212** | **0.003782** | **-4.17E-07** | **-3.03E-07** | **-2.61E-07** |
| **7.0** | **17.02334** | **0.949284** | **0.0037563** | **-2.26E-07** | **-1.05E-07** | **-2.84E-07** |
| **7.5** | **18.48325** | **0.945679** | **0.0041208** | **-4.60E-07** | **1.35E-07** | **-3.94E-07** |
| **8.0** | **20.28461** | **0.942331** | **0.0043534** | **-4.24E-07** | **-3.98E-07** | **-2.04E-07** |
| **8.5** | **21.39218** | **0.938384** | **0.0044145** | **-3.37E-07** | **6.35E-09** | **-1.24E-07** |
| **9.0** | **22.16908** | **0.934237** | **0.0048078** | **-9.03E-08** | **2.13E-07** | **-7.46E-08** |
| **9.5** | **23.85154** | **0.93033** | **0.0051242** | **1.54E-07** | **2.46E-07** | **-1.28E-07** |
| **10.0** | **25.88792** | **0.926305** | **0.0052077** | **-2.55E-08** | **4.40E-08** | **-4.38E-07** |
| **DF (µm)** | ***b_0_*** | ***b_1_*** | ***b_2_*** | ***b_3_*** | ***b_4_*** | ***b_5_*** |
| **0.0** | **7.02E-14** | **1.23E-16** | **1** | **-1.98E-20** | **-9.52E-22** | **6.78E-21** |
| **0.5** | **0.369406** | **0.0002735** | **0.996272** | **4.64E-08** | **-3.59E-08** | **-1.00E-07** |
| **1.0** | **0.033953** | **0.0003262** | **0.992444** | **-2.12E-08** | **-8.39E-08** | **-6.27E-08** |
| **1.5** | **-0.40756** | **0.0004037** | **0.988724** | **-1.11E-07** | **7.59E-08** | **-2.26E-07** |
| **2.0** | **-0.29858** | **0.0005389** | **0.98511** | **-1.59E-07** | **8.57E-09** | **-4.18E-07** |
| **2.5** | **-1.25611** | **0.0003346** | **0.981398** | **-1.37E-07** | **2.04E-07** | **-6.62E-07** |
| **3.0** | **-1.41381** | **0.0002162** | **0.977667** | **-1.49E-07** | **2.65E-07** | **-5.67E-07** |
| **3.5** | **-2.32527** | **0.0001067** | **0.974312** | **-1.29E-07** | **4.97E-07** | **-8.66E-07** |
| **4.0** | **-2.43049** | **-1.00E-06** | **0.970496** | **-2.60E-07** | **4.20E-07** | **-6.77E-07** |
| **4.5** | **-2.54883** | **-0.00044** | **0.967345** | **-1.97E-07** | **7.90E-07** | **-7.78E-07** |
| **5.0** | **-2.40529** | **-0.000537** | **0.963982** | **-3.55E-07** | **1.02E-06** | **-7.75E-07** |
| **5.5** | **-1.73446** | **-0.001155** | **0.960552** | **-3.15E-07** | **8.64E-07** | **-6.73E-07** |
| **6.0** | **-2.65163** | **-0.001518** | **0.956821** | **-2.26E-07** | **1.06E-06** | **-5.14E-07** |
| **6.5** | **-1.91267** | **-0.00154** | **0.953342** | **-3.20E-07** | **7.85E-07** | **-9.13E-07** |
| **7.0** | **-2.32387** | **-0.001831** | **0.949523** | **-7.56E-08** | **9.19E-07** | **-8.52E-07** |
| **7.5** | **-2.67529** | **-0.002057** | **0.945681** | **-5.84E-08** | **8.05E-07** | **-6.54E-07** |
| **8.0** | **-1.80849** | **-0.002104** | **0.942146** | **-1.32E-07** | **8.29E-07** | **-9.62E-07** |
| **8.5** | **-1.9478** | **-0.002706** | **0.937803** | **1.07E-07** | **8.69E-07** | **-7.80E-07** |
| **9.0** | **-1.58157** | **-0.003083** | **0.934396** | **-7.49E-08** | **6.82E-07** | **-9.22E-07** |
| **9.5** | **-0.8561** | **-0.003179** | **0.930418** | **-2.93E-07** | **3.64E-07** | **-7.60E-07** |
| **10.0** | **-2.10867** | **-0.003357** | **0.92662** | **4.68E-09** | **7.70E-07** | **-7.13E-07** |
