# Supplementary material for: IPET and FETR: Experimental Approach for Studying Molecular Structure Dynamics by Cryo-Electron Tomography of a Single-Molecule Structure
Source: PLoS One. 2012 Jan 24;7(1):e30249. doi: 10.1371/journal.pone.0030249 (PMC3265479; doi:10.1371/journal.pone.0030249)
Supplement: Information S1 — Effect of missing wedge on 3D reconstruction is limited to high-resolution ET reconstruction of small and thin objects. (DOC) [file pone.0030249.s029.doc]

**Information S1**

Since the specimen holder can be tilted up to ~70°, the tilt limitation leads to a wedge-shaped area of data absent during reconstruction. It is often believed that the effects from the missing wedge could be significant. However, we believe that the effect of this tilt limitation is limited with thin objects such as proteins. One piece of indirect evidence is that 3D reconstructions from 2D crystals with ~18% of missing-cone data resulting from tilt-angle limitations of ~55° can achieve atomic resolution . By comparison, ET 3D reconstruction has ~22% missing-wedge data resulting from tilt-angle limitations (up to ~70° tilt), which is fairly close to the ~18% missing-cone data in the 3D reconstruction of 2D crystals. Therefore, we believe that for thin and small objects, such as protein molecules, the effect of the tilt on 3D reconstruction is limited.

To further quantitatively analyze the effect of missing-wedge data on 3D reconstruction, we performed the following tests: Three sets of projections, each containing 84 projections, were generated from same object but from differing sets of tilt angles. One set of tilt angles was that used by the single-particle reconstruction method (a sampling angle of 15°). The other two sets of different angles were used by the ET reconstruction method, with the tilt angle ranging evenly from -70° to +70° and -90° to +90°, respectively. To analyze the effect of the different sets of tilt angles on the 3D reconstruction, a 3D reconstruction was generated for each set of angles (**Figure S24A-24C**) and would be referred to as the single-particle map, the ET map (from the -70° to +70° set), and the “ideal” ET map (the -90° to +90° set), respectively. Only the ET map contained a missing wedge, while the other two maps did not. In comparing each of the three maps with the object, each displayed nearly all the structural features of the object and was essentially similar to one another, with no obvious differences (**Figure S24A-S24C**). To quantitatively analyze the difference among the maps, the FSC curves and CC C values between the object and each map were computed. The FSC curves showed that the *f_0.5_* values (**Figure S24D**) were beyond the Nyquist frequency, suggesting that all three maps were similar to the actual object. For FSC values above 0.87, the ET map was the least similar to the object, while for FSC values between 0.58 and 0.87, for the single-particle map was the least similar (**Figure S24D**). The CC C values showed that all the maps had CC C values were above 0.68, suggesting a high degree of similarity between each reconstruction and the actual object (**Figure S24E**). This analysis suggests that the overall quality of the ET reconstruction is similar to that of the single-particle map, and this result is consistent with experimental results in 2D cryocrystallography. Similar discussion about the missing-wedge effects on the thin and small objects have been discussed by Klug and Crowther decades ago . Thus, we believe that the missing wedge is not a major obstacle to high-resolution ET reconstruction for small and thin objects.

**References**

1. Ren G, Reddy VS, Cheng A, Melnyk P, Mitra AK (2001) Visualization of a water-selective pore by electron crystallography in vitreous ice. Proc Natl Acad Sci U S A 98: 1398-1403.

2. Ren G, Cheng A, Reddy V, Melnyk P, Mitra AK (2000) Three-dimensional fold of the human AQP1 water channel determined at 4 A resolution by electron crystallography of two-dimensional crystals embedded in ice. J Mol Biol 301: 369-387.

3. Klug A, Crowther RA (1972) Three-dimensional Image Reconstruction from the Viewpoint of information Theory. Nature Structural and Molecular Biology 238: 435 - 440.
